# Author Correction: An all-to-all approach to the identification of sequence-specific readers for epigenetic DNA modifications on cytosine

**DOI:** 10.1038/s41467-021-21726-y

**Published:** 2021-02-23

**Authors:** Guang Song, Guohua Wang, Ximei Luo, Ying Cheng, Qifeng Song, Jun Wan, Cedric Moore, Hongjun Song, Peng Jin, Jiang Qian, Heng Zhu

**Affiliations:** 1 Department of Pharmacology and Molecular Sciences, Johns Hopkins University School of Medicine, Baltimore, MD USA; 2 School of Computer Science and Technology, Harbin Institute of Technology, Harbin, Heilongjiang China; 3 Department of Ophthalmology, Johns Hopkins University School of Medicine, Baltimore, MD USA; 4 Department of Human Genetics, Emory University School of Medicine, Atlanta, GA USA; 5 Institute of Biomedical Research, Yunnan University, Kunming, Yunnan China; 6 Department of Neuroscience and Mahoney Institute for Neurosciences, University of Pennsylvania, Philadelphia, PA USA

**Keywords:** Epigenetics analysis, High-throughput screening, Assay systems, Screening, Epigenetics

Correction to: *Nature Communications* 10.1038/s41467-021-20950-w, published online 04 February 2021.

In the original version of this Article, the “Methods” section “Genome-wide hmC profiling of human embryonic stem cell H1” incorrectly stated “"Human embryonic stem cell H1 was purchased from WiCell Research Institute (WiCell) and the ethics approval was obtained from the Robert-Koch Institute, Berlin, Germany.”. Ethical approval was not required for the use of hESC H1 cells purchased from WiCell Research Institute. The statement has been corrected to “Human embryonic stem cell H1 was purchased from WiCell Research Institute (WiCell).” This has been corrected in the HTML and PDF version of this Article.

