# Supplementary material for: An all-to-all approach to the identification of sequence-specific readers for epigenetic DNA modifications on cytosine
Source: Nat Commun. 2021 Feb 4;12:795. doi: 10.1038/s41467-021-20950-w (PMC7862700; doi:10.1038/s41467-021-20950-w)
Supplement: Supplementary file 1 — Supplementary information [file 41467_2021_20950_MOESM1_ESM.pdf]

Supplementary information

An all-to-all approach to the identification of sequence-specific readers for epigenetic DNA modifications on cytosine

Guang Song[#], Guohua Wang[#], Ximei Luo[#], Ying Cheng, Qifeng Song, Jun Wan, Cedric Moore, Hongjun Song, Peng Jin, Jiang Qian[*], Heng Zhu[*]

[#]These authors contributed equally to this work.

[*]These authors jointly supervised this work.

Correspondence should be addressed to H.Z. (email: hzhu4@jhmi.edu) or to J.Q. (email: jiang.qian@jhmi.edu)

This PDF file includes:

Supplementary Fig. 1 to 9

Supplementary Table 1

a Anchor oligo:
maleimide-5' TACGATCATAAACTTAGTTCACTTCAACAAGAATGATAT GATTTAGTTCAGGACGTAGG
Spacer Primer 1

b Barcode oligo:
5'-GACTCACGGTCTCC↓GACTGGNNNNNNNNACTATTAAAGGCCCTACGTCCTGAACTAAATC
BsaI UMI Barcode Primer 1 rev

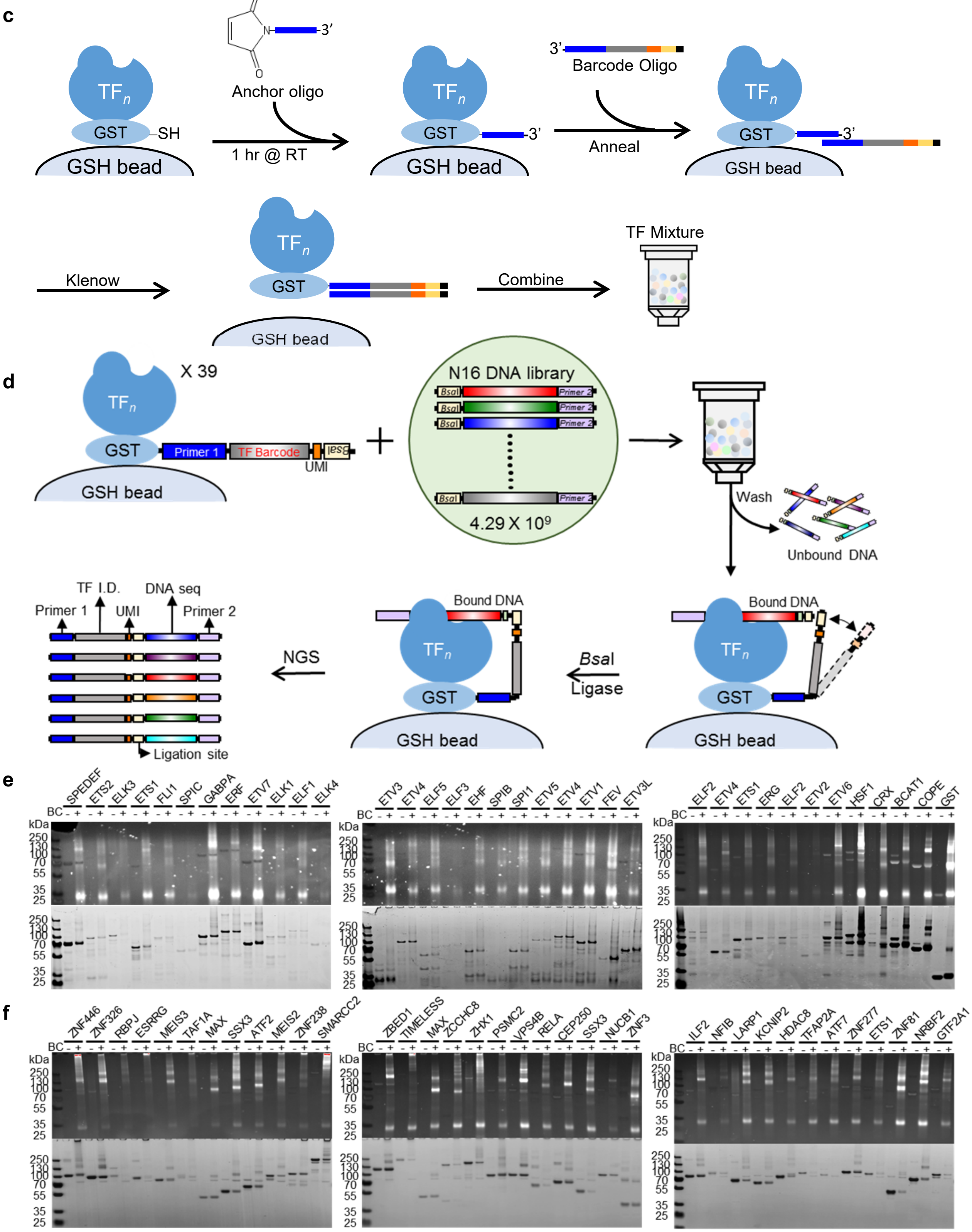

Supplementary Figure 1. DNA-barcoded human TF proteins and design of DAPPL.

a Design of the anchor oligo with a maleimide-2, 5-dimethylfuran moiety attached at the 5'-end. **b** Design of the barcode oligos annotated with *Bsa*I's recognition (yellow) and cutting site (arrow), a random 8-mer unique molecule identifier (UMI; red), a barcode sequence (7-11 nt in length), and a 20-nt consistent sequence complementary to Primer 1 in anchor oligo. **c** Procedure of barcoding TF proteins with dsDNA. **d** Schematics of identifying ETS reading sequences using a 16-mer randomized DNA libraries using the digital affinity profiling via proximity ligation (DAPPL) approach. The multicolored middle bricks of each DNA means various randomly synthesized DNA sequence species. **e** ETS family TFs and control proteins were examined for the efficiency of DNA-barcoding via Coomassie and EB co-staining in the same gel. **f** Examination of DNA-barcoded 36 randomly selected TFs as above in DAPPL to identify TF readers for epigenetic modifications.

a

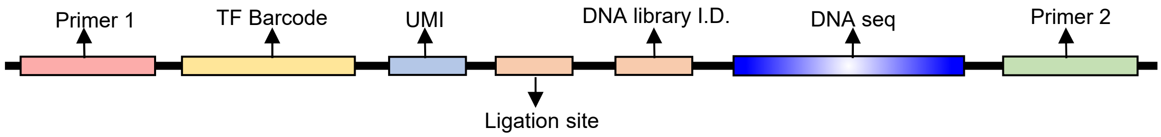

b

	Cycle1_Rep 1	Cycle1_Rep 2	Cycle1_Rep 3	Cycle 2	Total
**Primer 1**	91.13%	91.43%	90.89%	95.73%	91.93%
**TF Barcode**	60.87%	60.49%	67.52%	68.38%	63.68%
**Length of UMI**	76.96%	76.85%	84.81%	83.86%	80.02%
**Ligation site and Library I.D.**	72.84%	75.22%	66.01%	78.11%	72.66%
**Primer 2**	79.94%	89.04%	82.89%	79.82%	82.94%

Supplementary Figure 2. Processing statistics of raw sequencing data.

a The expected structure of the digital affinity profiling via proximity ligation (DAPPL) products. The multicolored bricks mean the different components of the DAPPL product . **b** Percentages of the expected elements of the DAPPL products. Note that the percentages in the table are not additive, because some DAAPL products removed from further analyses contained more than one error.

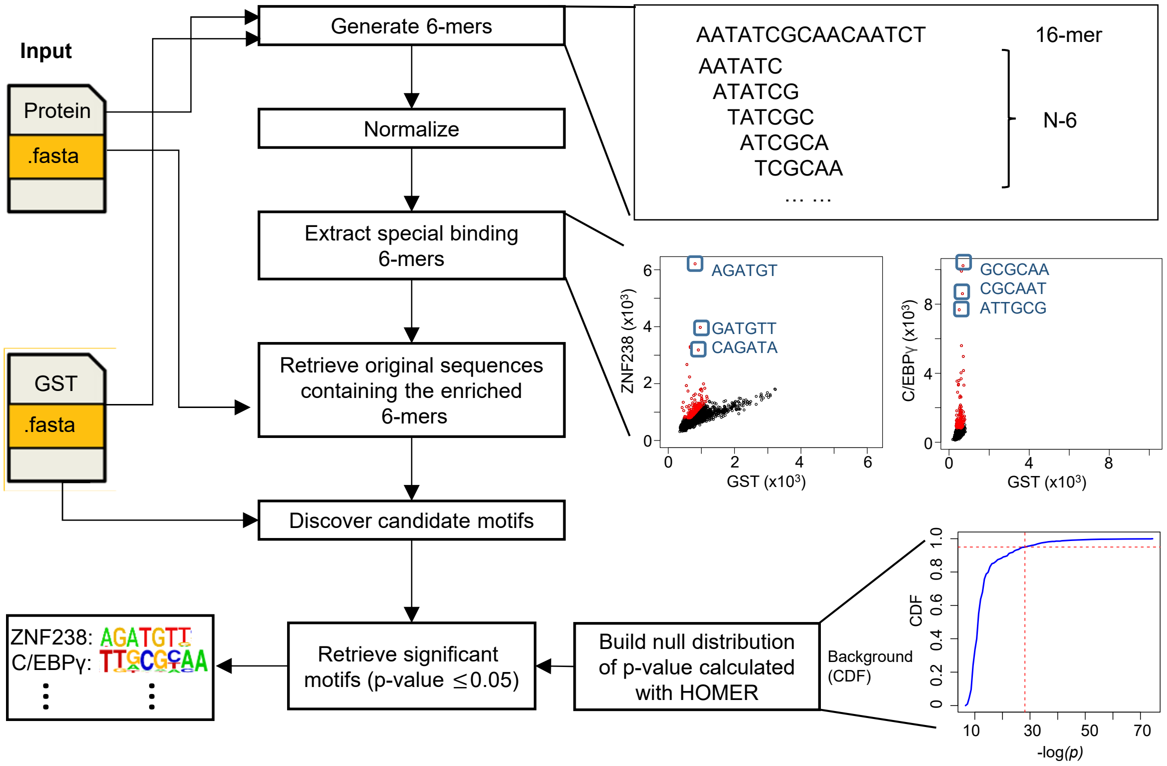

Supplementary Figure 3. Motif discovery pipeline and motif examples of TFs with multiple clones.

All of the binding sequences were extracted and assigned to each TF protein using the associated TF barcodes, followed by further partitioning using the corresponding DNA library barcodes. To identify the binding motifs for each TF, binding sequences were parsed into six base pair sequences (i.e. 6-mers). To exclude the contribution from GST, we compared the 6-mer frequencies between each TF and the GST counterparts. The 6-mers that were found enriched for the TFs were considered to be “seed” binding sequences (red spots), according to Formula I. The full-length sequences were retrieved for the enriched 6-mers and used to discover candidate motifs using HOMER. To determine the significance cutoff, we performed 400 random samplings of the GST-bound sequences with a sample size of 10,000 from each library. These sequences were used to identify binding motifs using HOMER and the corresponding 400 *P* values were used to construct a null distribution. The *P* value at 95 percentile in the distribution (a false discovery rate of 0.05) was used as the cutoff to determine the significant motifs.

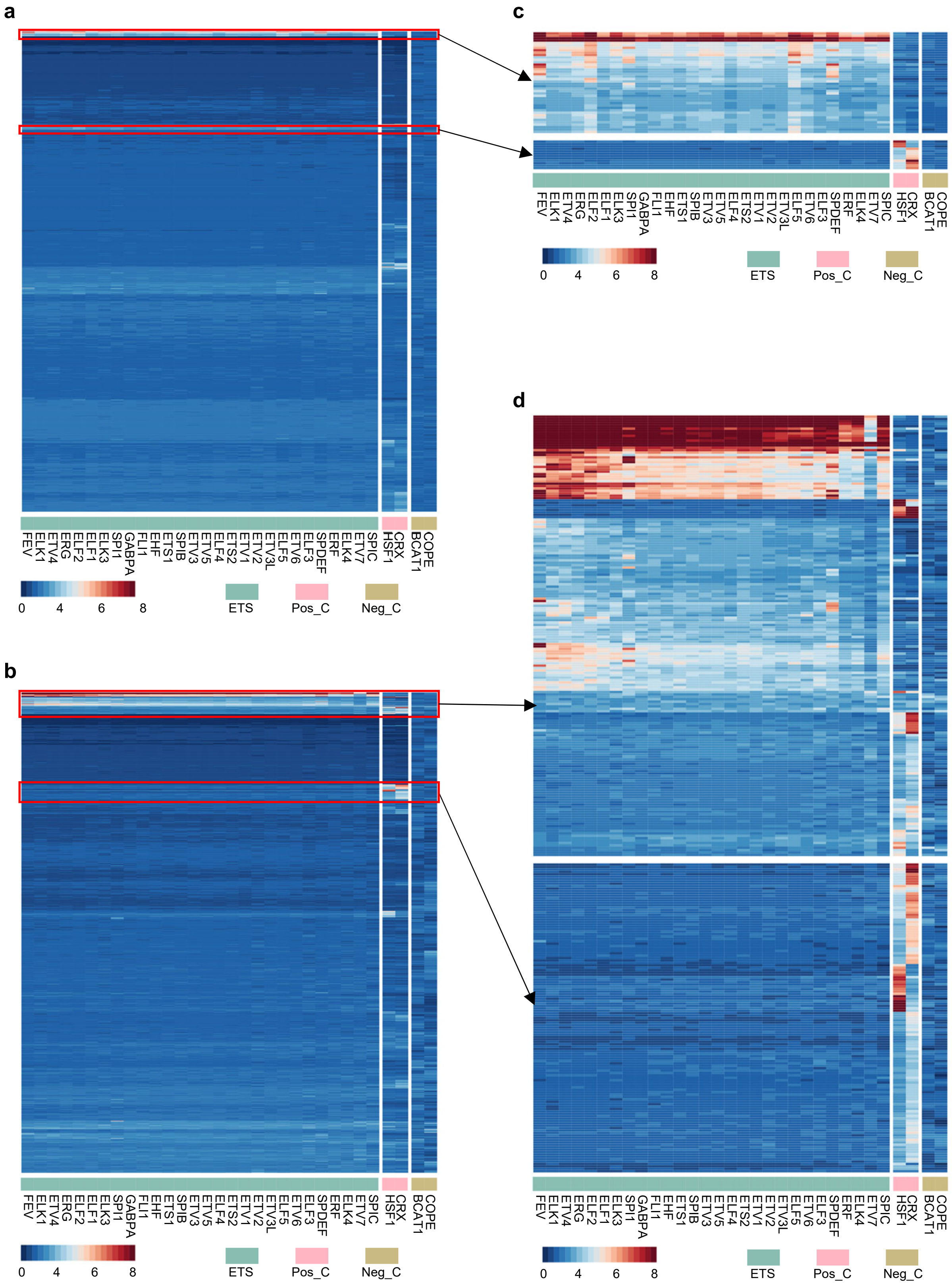

Supplementary Figure 4. Clustered heatmap density plots of 6-mers obtained with each TF.

a-b Heatmaps of 6-mers obtained with the 28 ETS, two positive controls (CRX and HSF1), and two negative controls (COPE and BCAT1) in cycle 1 (**a**) and cycle 2 (**b**) screenings. The scale bars represent the 6-mer frequency associated with a particular protein normalized by those obtained with GST. **c-d** Enlarged portions of the heatmaps where significantly enriched 6-mers were identified. Source data are provided as a Source Data file.

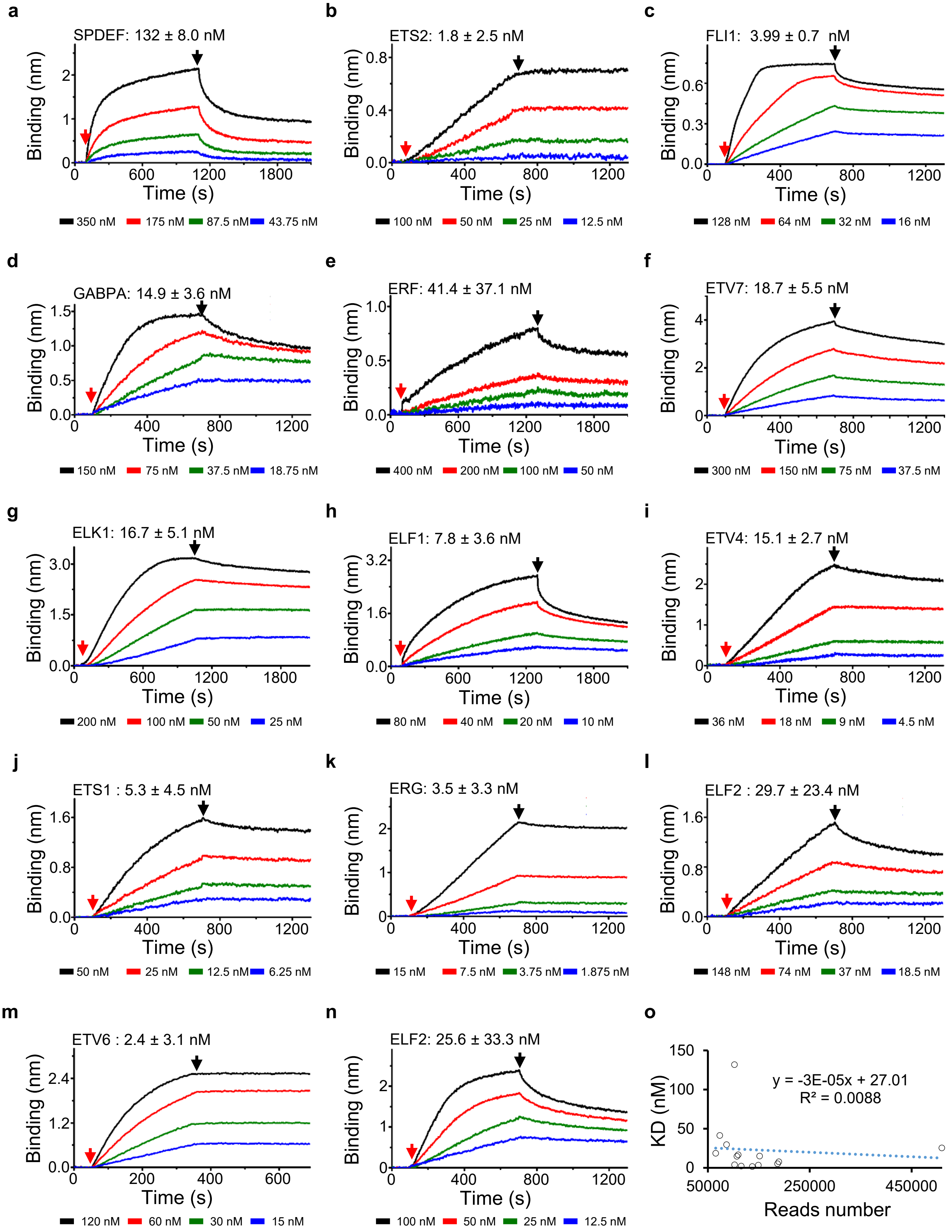
a
SPDEF: 132 ± 8.0 nM
Binding (nm)
Time (s)
350 nM 175 nM 87.5 nM 43.75 nM
b
ETS2: 1.8 ± 2.5 nM
Binding (nm)
Time (s)
100 nM 50 nM 25 nM 12.5 nM
c
FLI1: 3.99 ± 0.7 nM
Binding (nm)
Time (s)
128 nM 64 nM 32 nM 16 nM
d
GABPA: 14.9 ± 3.6 nM
Binding (nm)
Time (s)
150 nM 75 nM 37.5 nM 18.75 nM
e
ERF: 41.4 ± 37.1 nM
Binding (nm)
Time (s)
400 nM 200 nM 100 nM 50 nM
f
ETV7: 18.7 ± 5.5 nM
Binding (nm)
Time (s)
300 nM 150 nM 75 nM 37.5 nM
g
ELK1: 16.7 ± 5.1 nM
Binding (nm)
Time (s)
200 nM 100 nM 50 nM 25 nM
h
ELF1: 7.8 ± 3.6 nM
Binding (nm)
Time (s)
80 nM 40 nM 20 nM 10 nM
i
ETV4: 15.1 ± 2.7 nM
Binding (nm)
Time (s)
36 nM 18 nM 9 nM 4.5 nM
j
ETS1 : 5.3 ± 4.5 nM
Binding (nm)
Time (s)
50 nM 25 nM 12.5 nM 6.25 nM
k
ERG: 3.5 ± 3.3 nM
Binding (nm)
Time (s)
15 nM 7.5 nM 3.75 nM 1.875 nM
l
ELF2 : 29.7 ± 23.4 nM
Binding (nm)
Time (s)
148 nM 74 nM 37 nM 18.5 nM
m
ETV6 : 2.4 ± 3.1 nM
Binding (nm)
Time (s)
120 nM 60 nM 30 nM 15 nM
n
ELF2: 25.6 ± 33.3 nM
Binding (nm)
Time (s)
100 nM 50 nM 25 nM 12.5 nM
o
KD (nM)
y = -3E-05x + 27.01
R² = 0.0088
Reads number

Supplementary Figure 5. Binding affinity of ETS family.

a-n Binding kinetics and affinity studies of 14 random selected ETS proteins using OCTET. The red and black arrows indicate when the DNA probe sensors were dipped into the ETS protein solutions and wash buffer, respectively. Affinity values (K_D) are represented as mean ± SD and deduced from the K_{on} and K_{off} values obtained in four independent assays performed at four different concentrations (shown in different colors) of each ETS protein. The *y*-axis represents the nanometer (nm) shift used to define the biosensor surface changes. (o) Correlation of binding affinity (*y*-axis) versus the read number in the Nextgen sequencing (*x* axis).

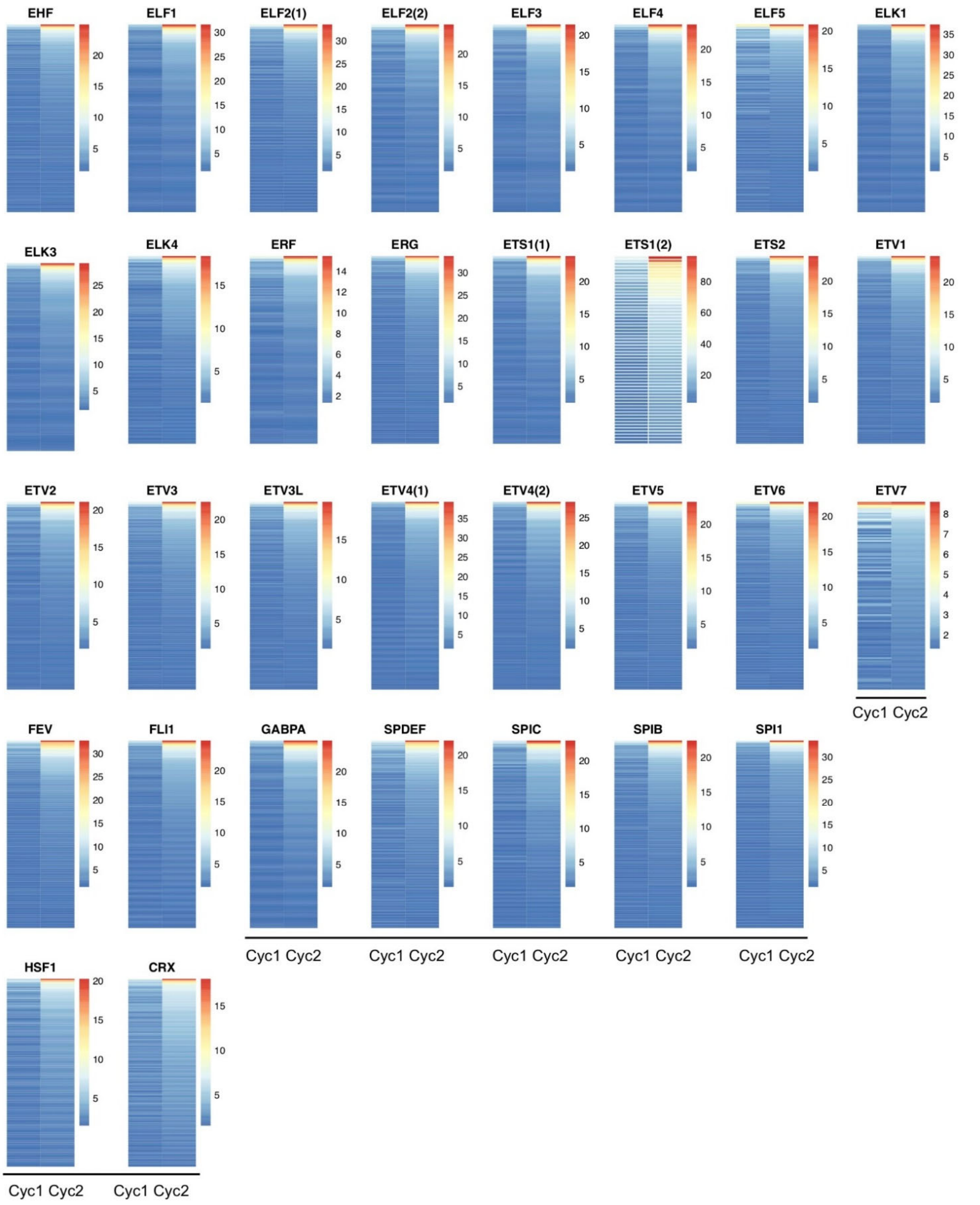

Supplementary Figure 6. Comparison of 6-mer frequency obtained between cycle 1 and cycle 2 of the DAPPL reactions.

Heatmap plots of the 6-mer frequencies were generated for the 28 ETS, HSF1 and CRX in cycle 1 (left column) and cycle 2 (right column) of the DAPPL reactions. Each row represents the 6-mer frequency associated with a particular protein normalized by those obtained with GST. The scale bars represent the 6-mer frequency associated with the TF normalized by those obtained with GST control.

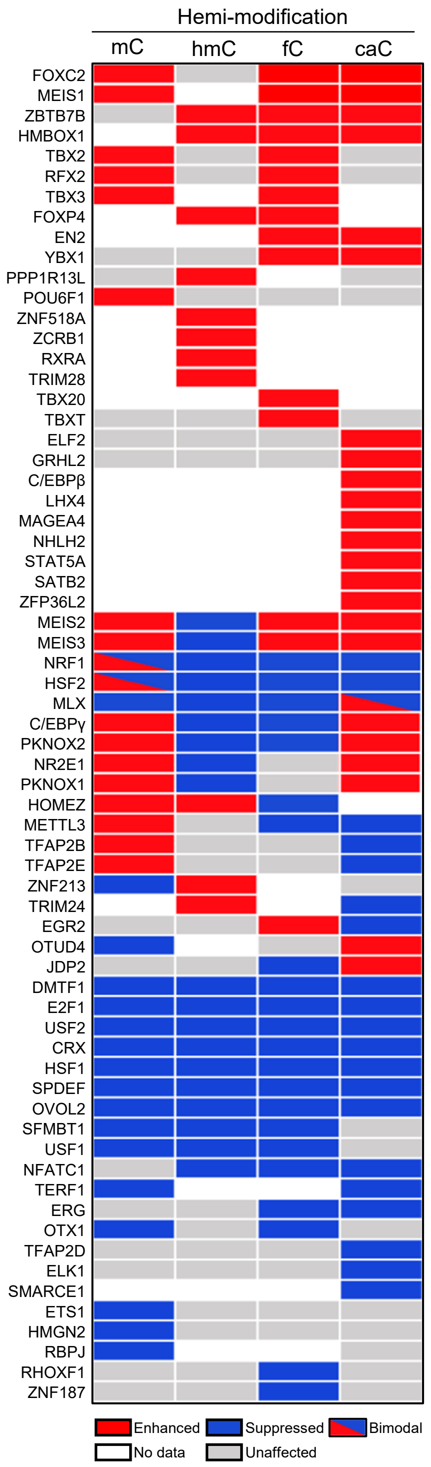

Supplementary Figure 7. Impact of hemi-modifications on TF-DNA interactions.
A given TF-DNA interaction can be enhanced (red bricks), suppressed (blue bricks), or unaffected (grey bricks) by any of the four hemi epigenetic modifications. Additionally, some modifications could enhance and suppress the binding strength of a particular TF depending on the sequence context (bimodal).

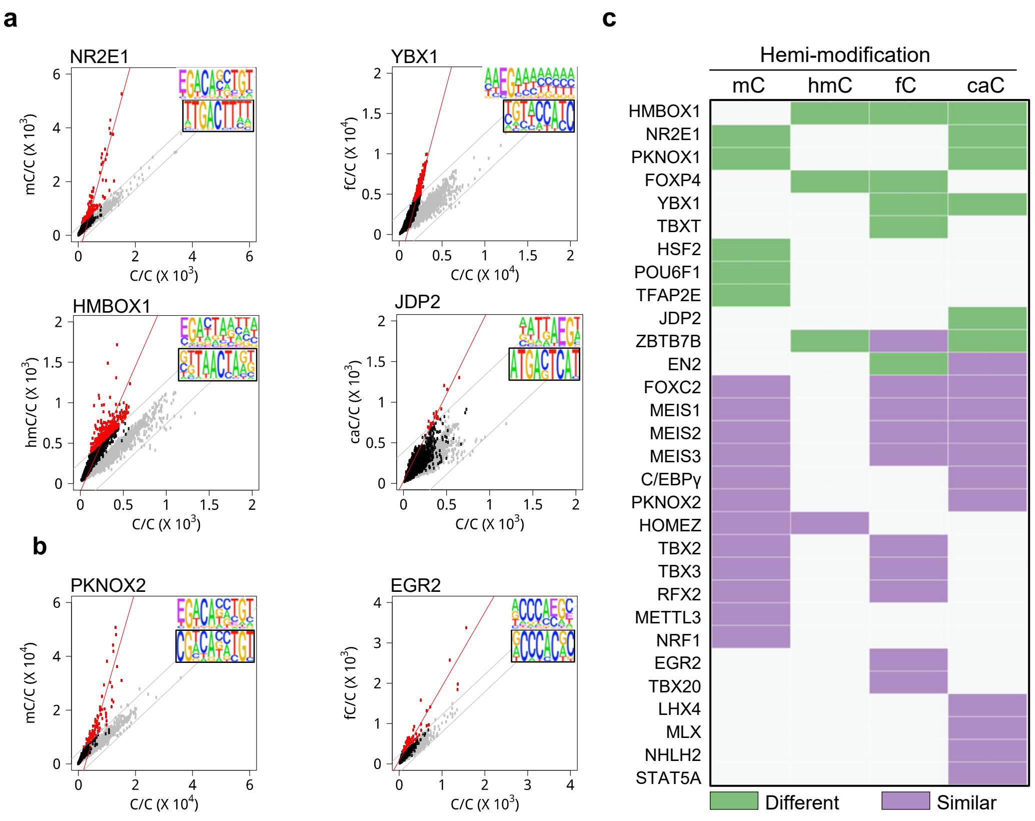

Supplementary Figure 8. Impact of hemi-modifications on TF-DNA binding specificity.

a Examples of deviated consensus sequences affected across all four epigenetic modifications. Known consensus sequences (boxed) in the absence of the modifications are compared with those carrying a modified cytosine. The X- and Y-axes represent the 6-mer frequency of the unmodified (e.g., C/C) and hemi-modified (e.g., hmC/C) DNA sequences captured by the TF, respectively. **b** Examples (i.e., PKNOX2 and EGR2) of TF binding specificity not affected by cytosine modifications, although with slightly enhanced binding affinity. **c** Summary of the impact of various hemi-modifications on the TF binding specificity. Green or purple bricks represent the consensus sequences generated from hemi-modified DNA libraries are different or similar with that from unmodified DNA libraries, respectively. Source data are provided as a Source Data file.

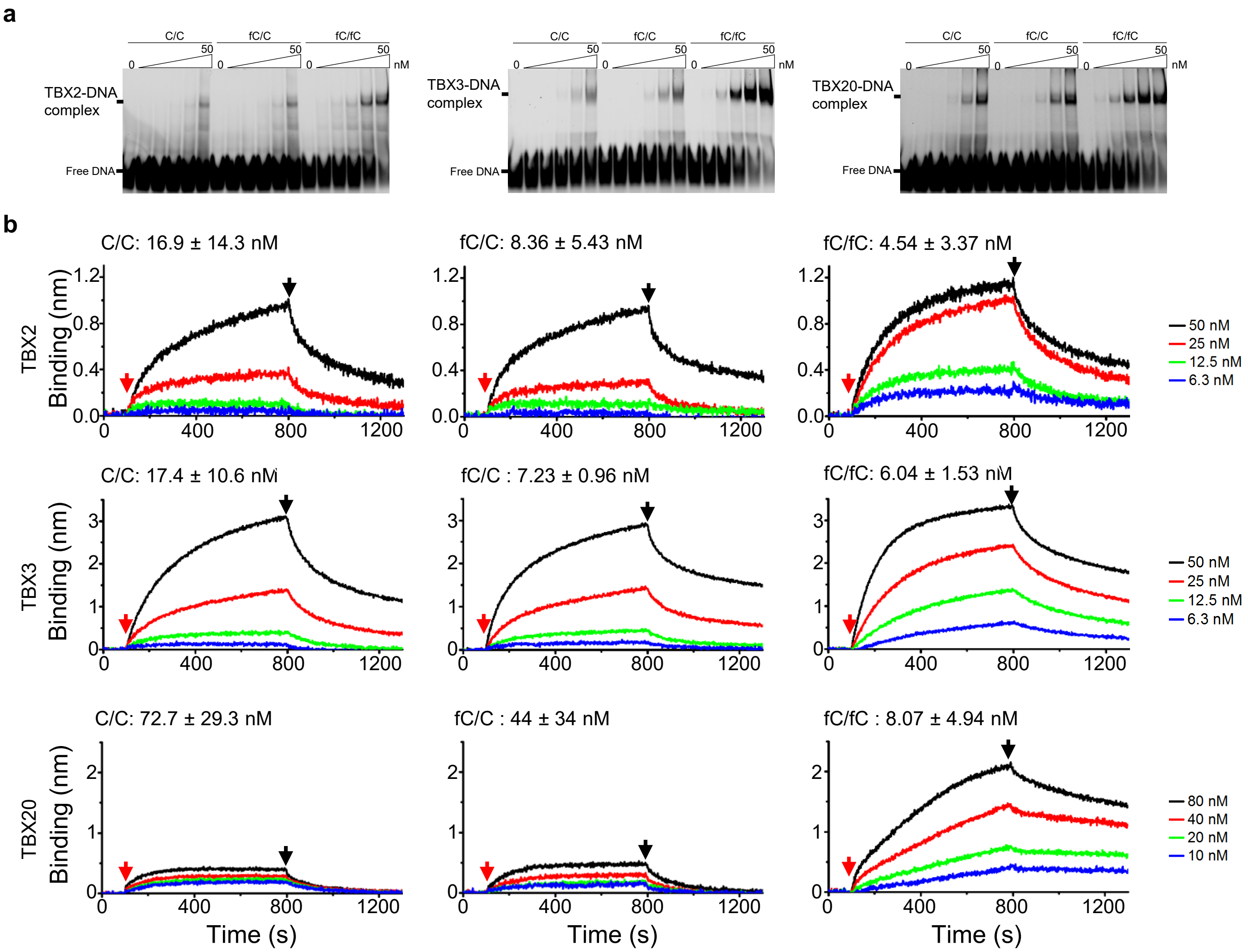

Supplementary Figure 9. Additional validation for TBX2, TBX3 and TBX20.

a Comparison of binding strength between three TBX paralogs and DNA probes using EMSA. The sequence of the probe used was 5'-T(A/C)AXGCCTC, where X indicates an unmodified cytosine or formylcytosine in either hemi or symmetric form. **b** Binding kinetics and affinity studies of the TBX proteins. The red and black arrows indicate when the DNA probe sensors were dipped into the TBX protein solutions and wash buffer, respectively. Affinity values (K_D) are represented as mean ± SD and deduced from the K_{on} and K_{off} values obtained in four independent assays performed at four different concentrations (shown in different colors) of each TBX protein. The y-axis represents the nanometer (nm) shift used to define the biosensor surface changes.

Supplementary Table

Supplementary Table 1. DNA oligo sequences used for the DAPPL and validation assays.

Oligo name	DNA oligo sequence	Notes
Anchor oligo	Maleimide-5'-AAACTTAGTTCACTTCAACAAGAATGATATGATTTAGTTCAGGACGTAGG	
Address oligos	5’-GACTCACGGTCTCCGACTGGNNNNNNNNACTATTAAAGGCCCTACGTCCTGAACTAAATC	
DNA16 lib	5’-GGGAGAAGGTCATCAAGAGG(N1:30302020)(N1)(N1)(N1)(N1)(N1)(N1)(N1)(N1)(N1)(N1)(N1)(N1)(N1)(N1)(N1)GGCATGCAGCCACTATAAGCTTCGAAGACTTGAGACCAT	
DNA16 lib rev	5’-ATGGTCTCAAGTCTTCGAAGCTTATAGTGGCTGCATGCC	
CpG	5’-CACATCCTTCACATTAATCC(N1:30302020)(N1)(N1)(N1)(N1)(N1)(N1)(N1)CG(N1)(N1)(N1)(N1)(N1)(N1)(N1)(N1)CATTCACTAACTAATCCGTACCTCGAGACTTGAGACCAT	Library ID: CGT
5mCpG	5’-CACATCCTTCACATTAATCC(N1:30302020)(N1)(N1)(N1)(N1)(N1)(N1)(N1)mCG(N1)(N1)(N1)(N1)(N1)(N1)(N1)(N1)CATTCACTAACTAATCAGCACCTCGAGACTTGAGACCAT	Library ID: AGC
5hmCpG	5’-CACATCCTTCACATTAATCC(N1:30302020)(N1)(N1)(N1)(N1)(N1)(N1)(N1)hmCG(N1)(N1)(N1)(N1)(N1)(N1)(N1)(N1)CATTCACTAACTAATCCTAACCTCGAGACTTGAGACCAT	Library ID: CTA
5fCpG	5’-CACATCCTTCACATTAATCC(N1:30302020)(N1)(N1)(N1)(N1)(N1)(N1)(N1)fCG(N1)(N1)(N1)(N1)(N1)(N1)(N1)(N1)CATTCACTAACTAATCAACACCTCGAGACTTGAGACCAT	Library ID: AAC
5caCpG	5’-CACATCCTTCACATTAATCC(N1:30302020)(N1)(N1)(N1)(N1)(N1)(N1)(N1)caCG(N1)(N1)(N1)(N1)(N1)(N1)(N1)(N1)CATTCACTAACTAATCATAACCTCGAGACTTGAGACCAT	Library ID: ATA
DNAlibrv	5’-ATGGTCTCAAGTCTCGAGG	
Bio-T7	5’-Biotin-TAATAGCACTCACTATA	
Cy5-T7	5’-Cy5-TAATAGCACTCACTATA	
T7	5’-TATAGTGAGTGCTATTA	
HOMEZ_probe oligo	5’-TCACTAAATATATXGATA(A/T)TATAGTGAGTGCTATTA	X = C, mC, hmC； For EMSA and OCTET validation experiments
STAT5A_probe oligo	5’-TCACTAAATATTXGNXGAATATAGTGAGTGCTATTA	X = C, caC; For EMSA and OCTET validation experiments
HMBOX1_probe oligo	5’-TCACTAAATAXGACTAATATAGTGAGTGCTATTA	X = C, mC, hmC, fC or caC; For EMSA validation experiments
OVOL2_probe oligo	5’-TCACTAAATACXGTTATATAGTGAGTGCTATTA	X = C, mC, hmC, fC or caC; For EMSA and OCTET validation experiments
TBX2/3/4_probe oligo	5’-TCACTAAATAT(A/C)AXGCCTCTATAGTGAGTGCTATTA	X = C, mC, hmC, fC or caC; For EMSA and OCTET validation experiments
DMTF1_probe oligo	5’-TCACTAAAGG(A/C/G)CATCCGTATAGTGAGTGCTATTA	For EMSA validation experiments
ETS family_probe oligo	5’-ACTTCCGGTTATAGTGAGTGCTATTA	For OCTET validation experiments
