# Supplementary material for: An all-to-all approach to the identification of sequence-specific readers for epigenetic DNA modifications on cytosine
Source: Nat Commun. 2021 Feb 4;12:795. doi: 10.1038/s41467-021-20950-w (PMC7862700; doi:10.1038/s41467-021-20950-w)
Supplement: Supplementary file 3 — Description of Additional Supplementary Files [file 41467_2021_20950_MOESM3_ESM.docx]

**Description of Supplementary Files**

**File Name: Supplementary Data 1**

**Description:** Barcode sequences designed for TFs and GST proteins.

**File Name: Supplementary Data 2**

**Description:** Consensus sequences identified by DAPPL. Consensus sequences identified for ETS and control transcription factors with the 16-mer DNA library using the DAPPL approach.

**File Name: Supplementary Data 3**

**Description:** Position probability matrix annotation of motifs identified using 16-mer dsDNA library.

**File Name: Supplementary Data 4**

**Description:** Identified readers for symmetric and hemi modifications.

**File Name: Supplementary Data 5**

**Description:** Position probability matrix annotation of motifs identified using symmetric and hemi modified DNA libraries.

**File Name: Supplementary Data 6**

**Description:** Clustered heatmap of 6-mers obtained with each TF in the DAPPL screenings for epigenetic readers. Heatmaps of 6-mers obtained with each TF in the DAPPL screenings for epigenetic readers. The scale bars represent the 6-mer frequency associated with a particular protein normalized by those obtained with GST.

**File Name: Supplementary Data 7**

**Description:** Impact of symmetric modifications to TF-DNA interactions.

Comparing the 6-mer frequencies obtained with each symmetrically modified library against the unmodified one for a given TF revealed that 14, 1, 7, and 28 TFs preferred methylation, hydroxymethylation, formylation, and carboxylation modifications, respectively; and that methylation, hydroxymethylation, formylation, and carboxylation suppressed binding activities of 21, 24, 25, and 25 TFs, respectively. We also observed that 9, 8, 6, and 2 TFs were insensitive to methylation, hydroxymethylation, formylation, or carboxylation, respectively.

**File Name: Supplementary Data 8**

**Description:** Impact of hemi modifications to TF-DNA interactions.

When the same analysis was applied to the results obtained with the four hemi modification libraries, we found that 18, 11, 15, and 24 TFs preferred methylation, hydroxymethylation, formylation, and carboxylation modifications, respectively; and that methylation, hydroxymethylation, formylation, and carboxylation suppressed binding activities of 19, 19, 22, and 21 TFs, respectively. We also observed that 14, 21, 12, and 14 TFs were insensitive to methylation, hydroxymethylation, formylation, or carboxylation, respectively.
