# Supplementary material for: An all-to-all approach to the identification of sequence-specific readers for epigenetic DNA modifications on cytosine
Source: Nat Commun. 2021 Feb 4;12:795. doi: 10.1038/s41467-021-20950-w (PMC7862700; doi:10.1038/s41467-021-20950-w)

## Slide 1
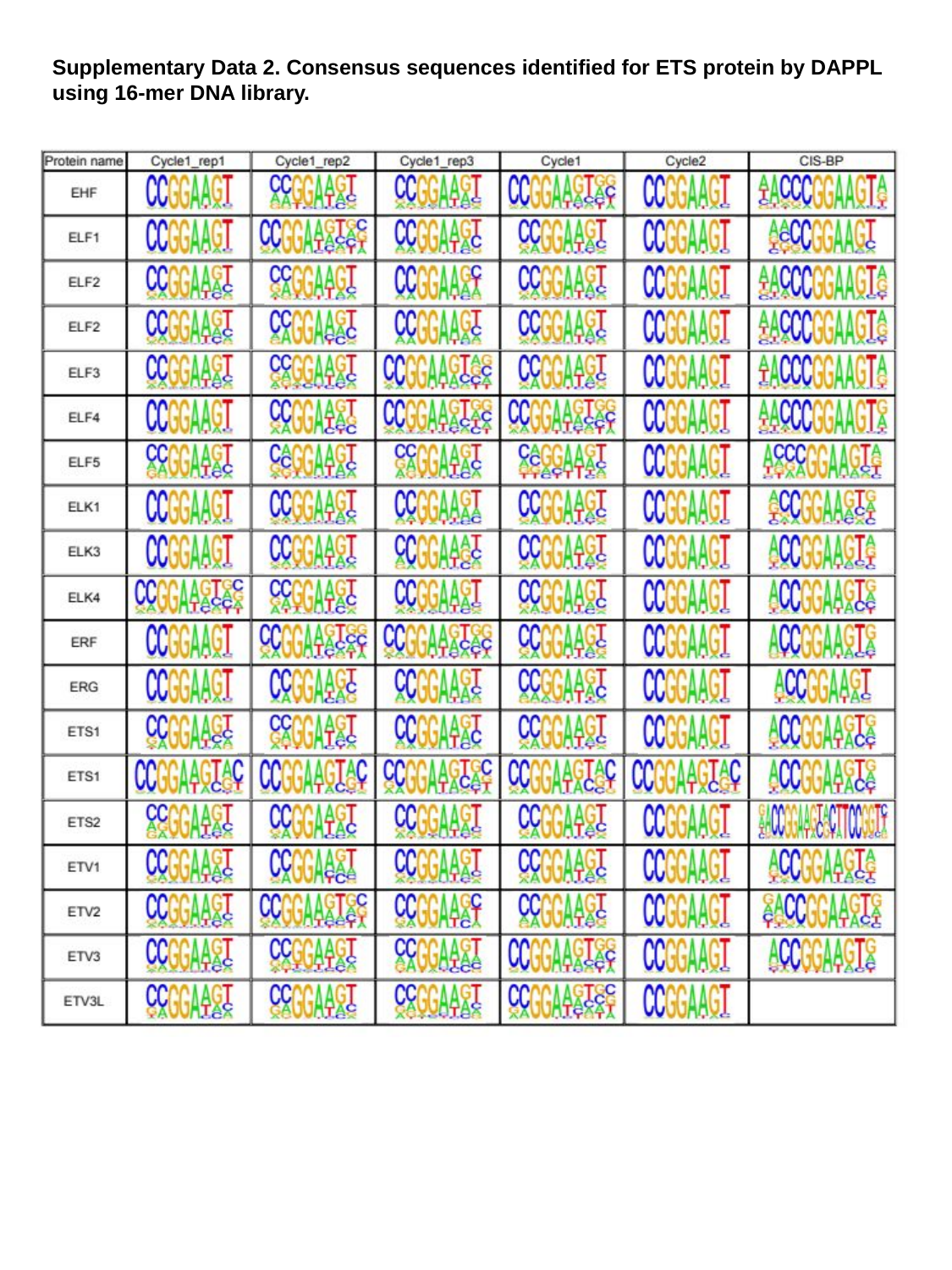

Supplementary Data 2. Consensus sequences identified for ETS protein by DAPPL using 16-mer DNA library.

## Slide 2
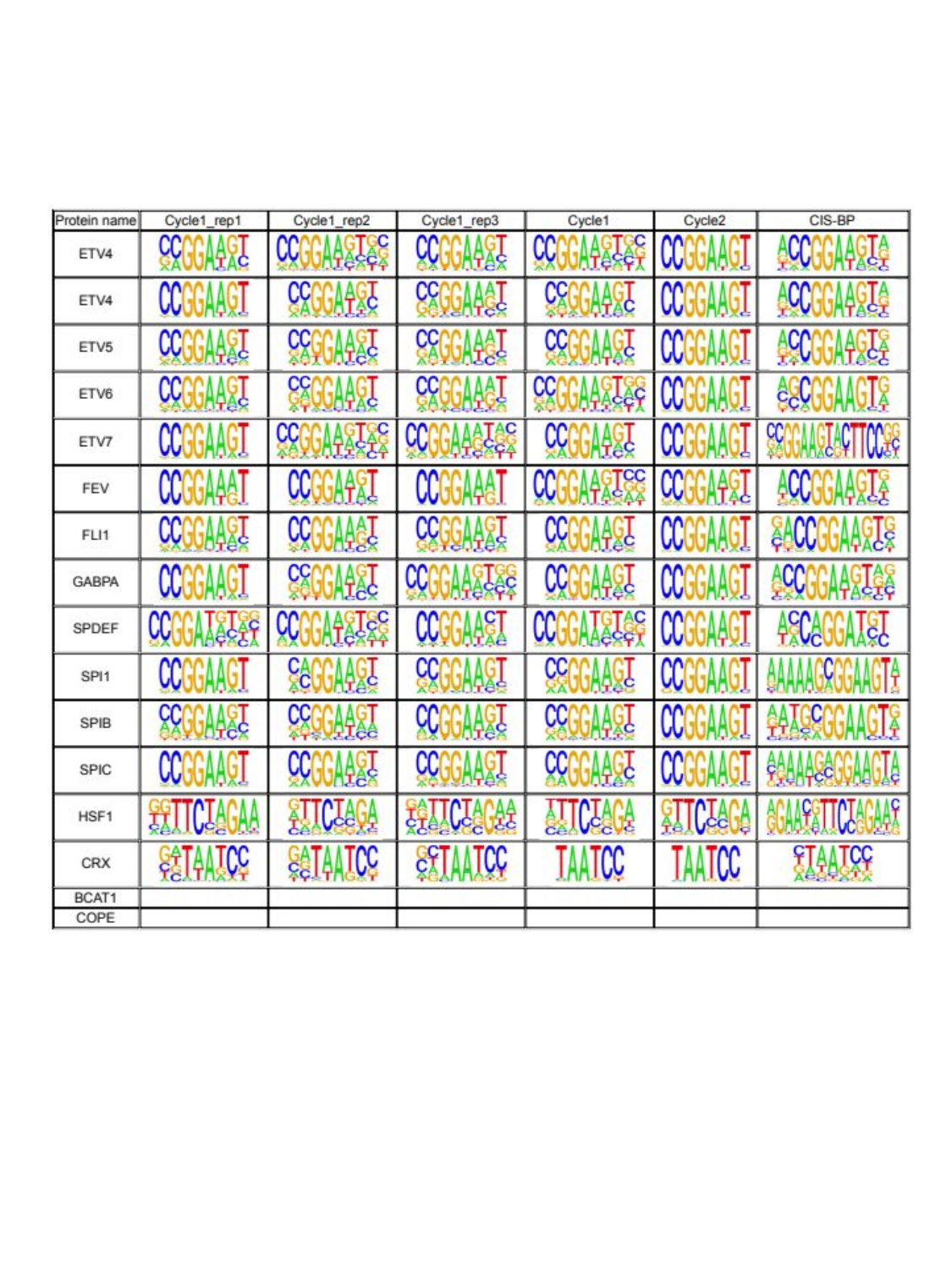

Supplement: Supplementary file 5 — Supplementary Data 2 [file 41467_2021_20950_MOESM5_ESM.pptx]
