# Supplementary material for: An all-to-all approach to the identification of sequence-specific readers for epigenetic DNA modifications on cytosine
Source: Nat Commun. 2021 Feb 4;12:795. doi: 10.1038/s41467-021-20950-w (PMC7862700; doi:10.1038/s41467-021-20950-w)
Supplement: Supplementary file 7 — Supplementary Data 4 [file 41467_2021_20950_MOESM7_ESM.pptx]

## Slide 1
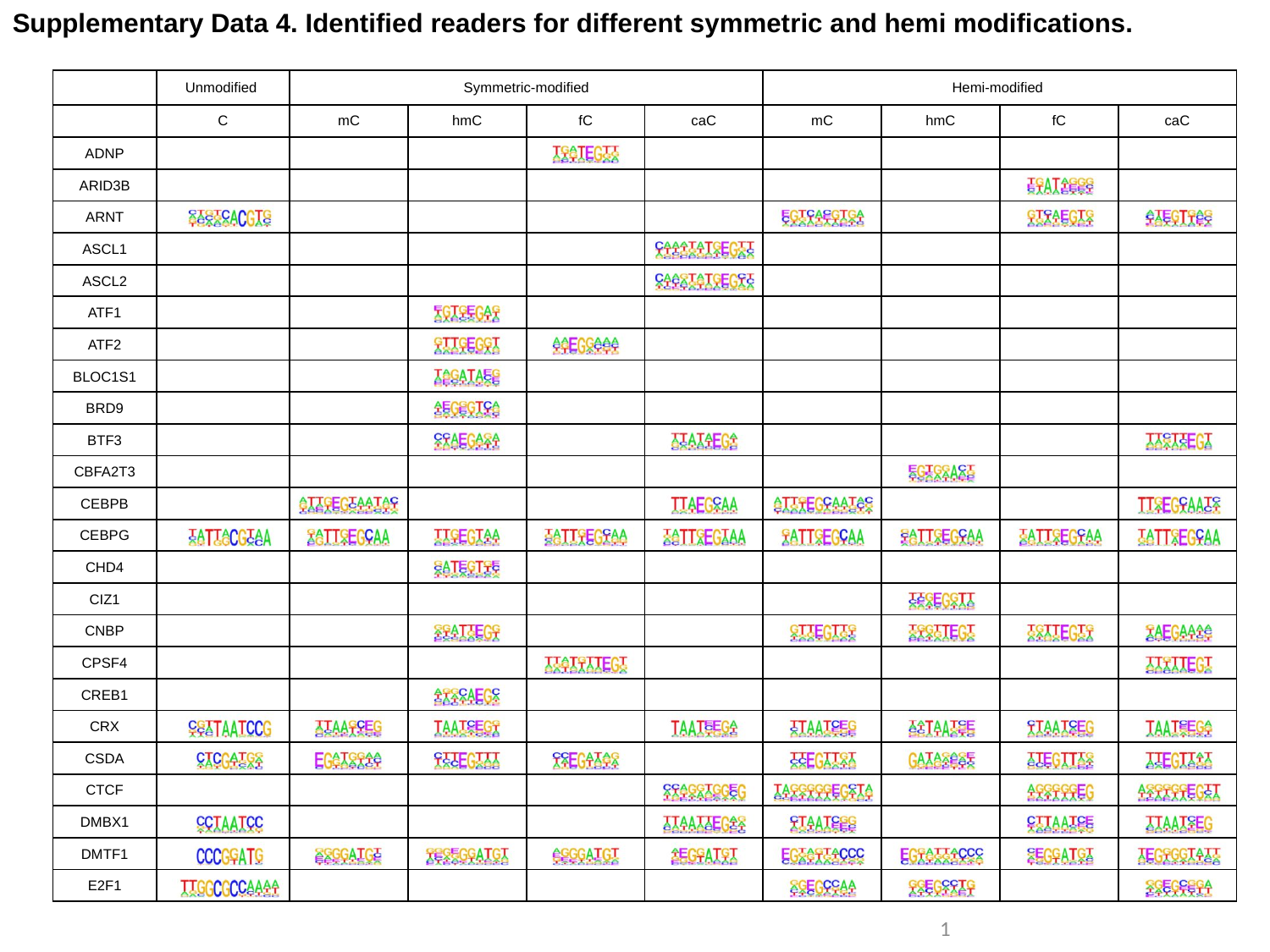

Supplementary Data 4. Identified readers for different symmetric and hemi modifications.
| | Unmodified | Symmetric-modified | | | | Hemi-modified | | | |
| --- | --- | --- | --- | --- | --- | --- | --- | --- | --- |
| | C | mC | hmC | fC | caC | mC | hmC | fC | caC |
| ADNP | | | | | | | | | |
| ARID3B | | | | | | | | | |
| ARNT | | | | | | | | | |
| ASCL1 | | | | | | | | | |
| ASCL2 | | | | | | | | | |
| ATF1 | | | | | | | | | |
| ATF2 | | | | | | | | | |
| BLOC1S1 | | | | | | | | | |
| BRD9 | | | | | | | | | |
| BTF3 | | | | | | | | | |
| CBFA2T3 | | | | | | | | | |
| CEBPB | | | | | | | | | |
| CEBPG | | | | | | | | | |
| CHD4 | | | | | | | | | |
| CIZ1 | | | | | | | | | |
| CNBP | | | | | | | | | |
| CPSF4 | | | | | | | | | |
| CREB1 | | | | | | | | | |
| CRX | | | | | | | | | |
| CSDA | | | | | | | | | |
| CTCF | | | | | | | | | |
| DMBX1 | | | | | | | | | |
| DMTF1 | | | | | | | | | |
| E2F1 | | | | | | | | | |
1

## Slide 2
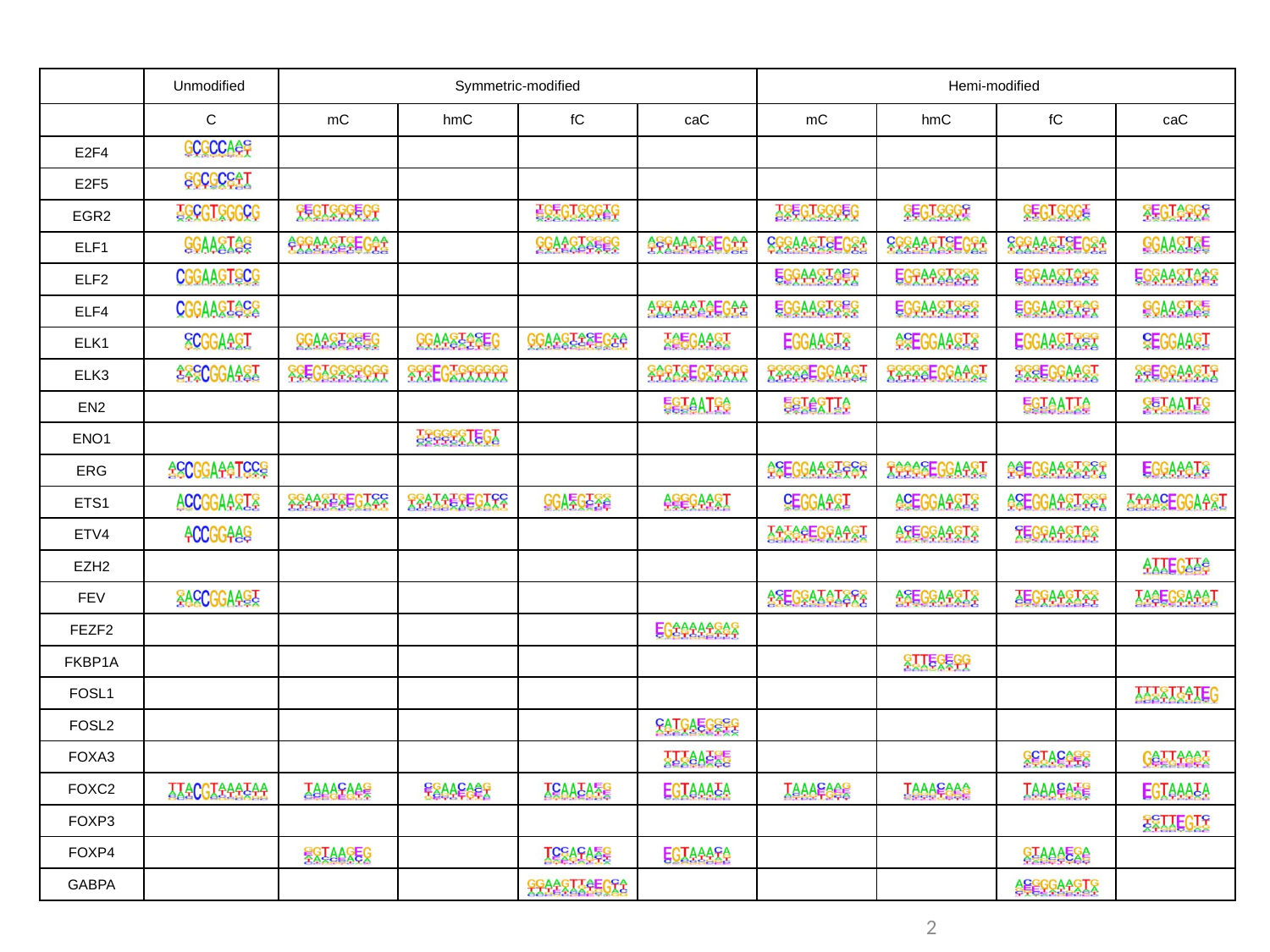

| | Unmodified | Symmetric-modified | | | | Hemi-modified | | | |
| --- | --- | --- | --- | --- | --- | --- | --- | --- | --- |
| | C | mC | hmC | fC | caC | mC | hmC | fC | caC |
| E2F4 | | | | | | | | | |
| E2F5 | | | | | | | | | |
| EGR2 | | | | | | | | | |
| ELF1 | | | | | | | | | |
| ELF2 | | | | | | | | | |
| ELF4 | | | | | | | | | |
| ELK1 | | | | | | | | | |
| ELK3 | | | | | | | | | |
| EN2 | | | | | | | | | |
| ENO1 | | | | | | | | | |
| ERG | | | | | | | | | |
| ETS1 | | | | | | | | | |
| ETV4 | | | | | | | | | |
| EZH2 | | | | | | | | | |
| FEV | | | | | | | | | |
| FEZF2 | | | | | | | | | |
| FKBP1A | | | | | | | | | |
| FOSL1 | | | | | | | | | |
| FOSL2 | | | | | | | | | |
| FOXA3 | | | | | | | | | |
| FOXC2 | | | | | | | | | |
| FOXP3 | | | | | | | | | |
| FOXP4 | | | | | | | | | |
| GABPA | | | | | | | | | |
2

## Slide 3
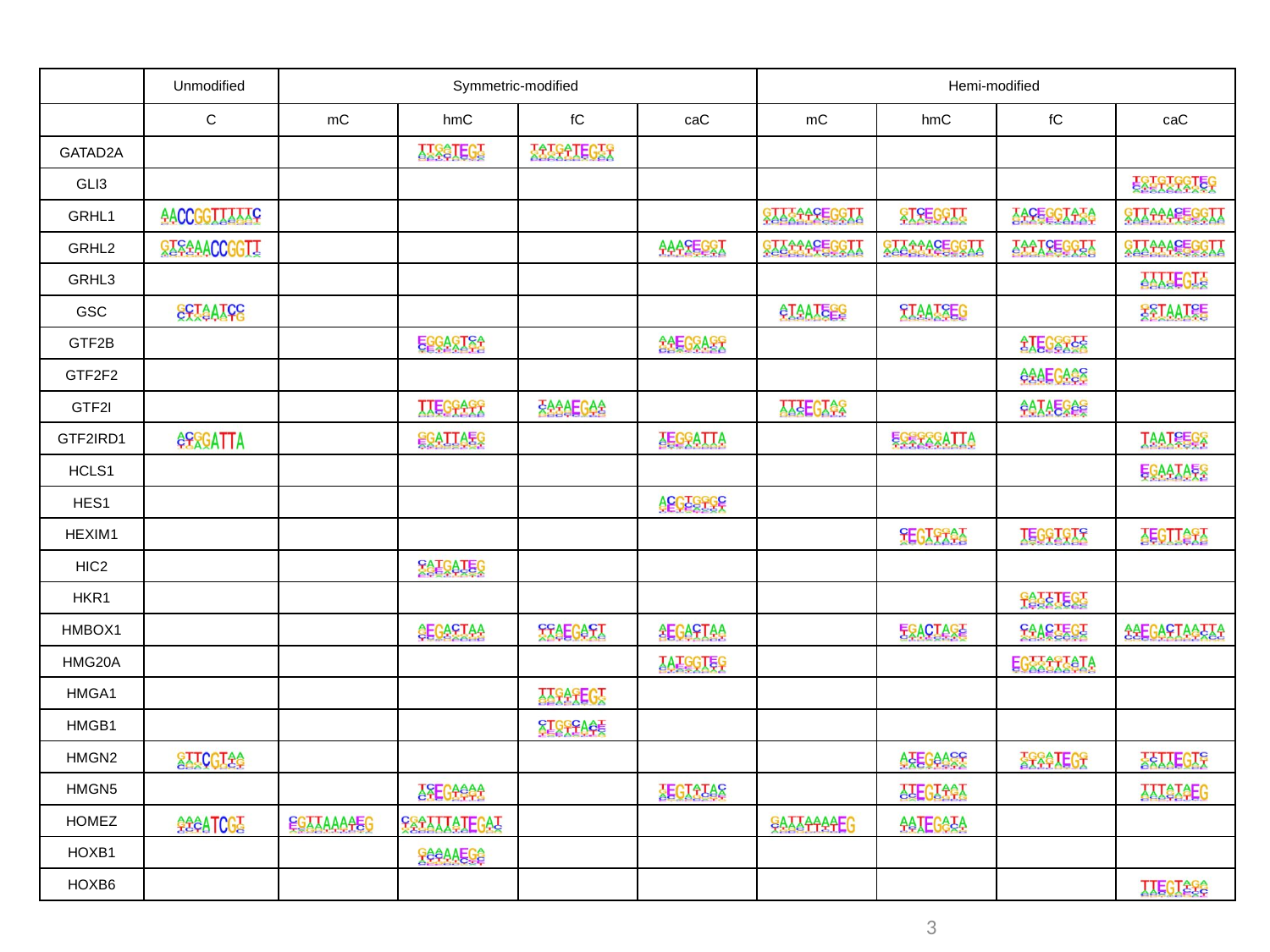

| | Unmodified | Symmetric-modified | | | | Hemi-modified | | | |
| --- | --- | --- | --- | --- | --- | --- | --- | --- | --- |
| | C | mC | hmC | fC | caC | mC | hmC | fC | caC |
| GATAD2A | | | | | | | | | |
| GLI3 | | | | | | | | | |
| GRHL1 | | | | | | | | | |
| GRHL2 | | | | | | | | | |
| GRHL3 | | | | | | | | | |
| GSC | | | | | | | | | |
| GTF2B | | | | | | | | | |
| GTF2F2 | | | | | | | | | |
| GTF2I | | | | | | | | | |
| GTF2IRD1 | | | | | | | | | |
| HCLS1 | | | | | | | | | |
| HES1 | | | | | | | | | |
| HEXIM1 | | | | | | | | | |
| HIC2 | | | | | | | | | |
| HKR1 | | | | | | | | | |
| HMBOX1 | | | | | | | | | |
| HMG20A | | | | | | | | | |
| HMGA1 | | | | | | | | | |
| HMGB1 | | | | | | | | | |
| HMGN2 | | | | | | | | | |
| HMGN5 | | | | | | | | | |
| HOMEZ | | | | | | | | | |
| HOXB1 | | | | | | | | | |
| HOXB6 | | | | | | | | | |
3

## Slide 4
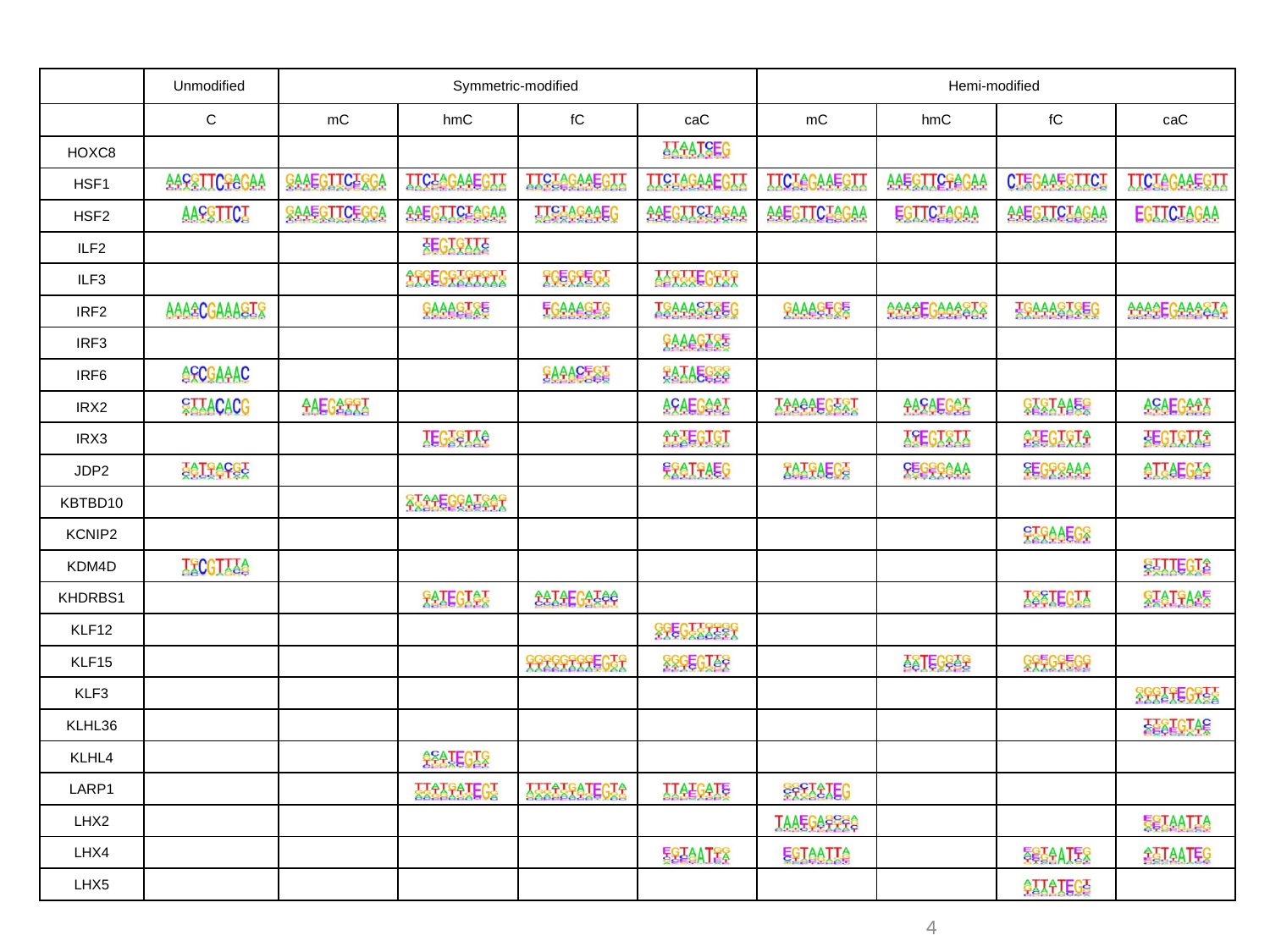

| | Unmodified | Symmetric-modified | | | | Hemi-modified | | | |
| --- | --- | --- | --- | --- | --- | --- | --- | --- | --- |
| | C | mC | hmC | fC | caC | mC | hmC | fC | caC |
| HOXC8 | | | | | | | | | |
| HSF1 | | | | | | | | | |
| HSF2 | | | | | | | | | |
| ILF2 | | | | | | | | | |
| ILF3 | | | | | | | | | |
| IRF2 | | | | | | | | | |
| IRF3 | | | | | | | | | |
| IRF6 | | | | | | | | | |
| IRX2 | | | | | | | | | |
| IRX3 | | | | | | | | | |
| JDP2 | | | | | | | | | |
| KBTBD10 | | | | | | | | | |
| KCNIP2 | | | | | | | | | |
| KDM4D | | | | | | | | | |
| KHDRBS1 | | | | | | | | | |
| KLF12 | | | | | | | | | |
| KLF15 | | | | | | | | | |
| KLF3 | | | | | | | | | |
| KLHL36 | | | | | | | | | |
| KLHL4 | | | | | | | | | |
| LARP1 | | | | | | | | | |
| LHX2 | | | | | | | | | |
| LHX4 | | | | | | | | | |
| LHX5 | | | | | | | | | |
4

## Slide 5
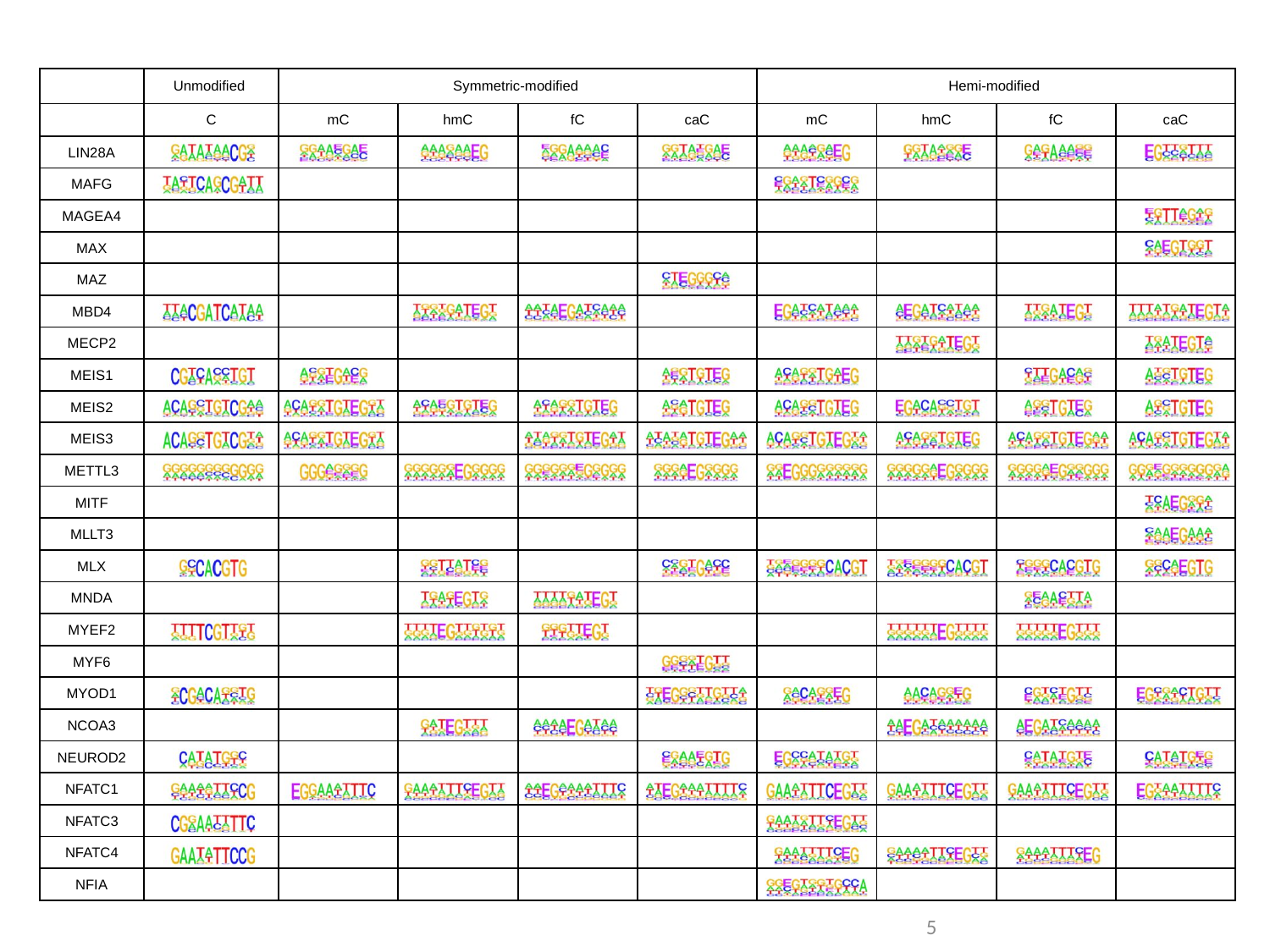

| | Unmodified | Symmetric-modified | | | | Hemi-modified | | | |
| --- | --- | --- | --- | --- | --- | --- | --- | --- | --- |
| | C | mC | hmC | fC | caC | mC | hmC | fC | caC |
| LIN28A | | | | | | | | | |
| MAFG | | | | | | | | | |
| MAGEA4 | | | | | | | | | |
| MAX | | | | | | | | | |
| MAZ | | | | | | | | | |
| MBD4 | | | | | | | | | |
| MECP2 | | | | | | | | | |
| MEIS1 | | | | | | | | | |
| MEIS2 | | | | | | | | | |
| MEIS3 | | | | | | | | | |
| METTL3 | | | | | | | | | |
| MITF | | | | | | | | | |
| MLLT3 | | | | | | | | | |
| MLX | | | | | | | | | |
| MNDA | | | | | | | | | |
| MYEF2 | | | | | | | | | |
| MYF6 | | | | | | | | | |
| MYOD1 | | | | | | | | | |
| NCOA3 | | | | | | | | | |
| NEUROD2 | | | | | | | | | |
| NFATC1 | | | | | | | | | |
| NFATC3 | | | | | | | | | |
| NFATC4 | | | | | | | | | |
| NFIA | | | | | | | | | |
5

## Slide 6
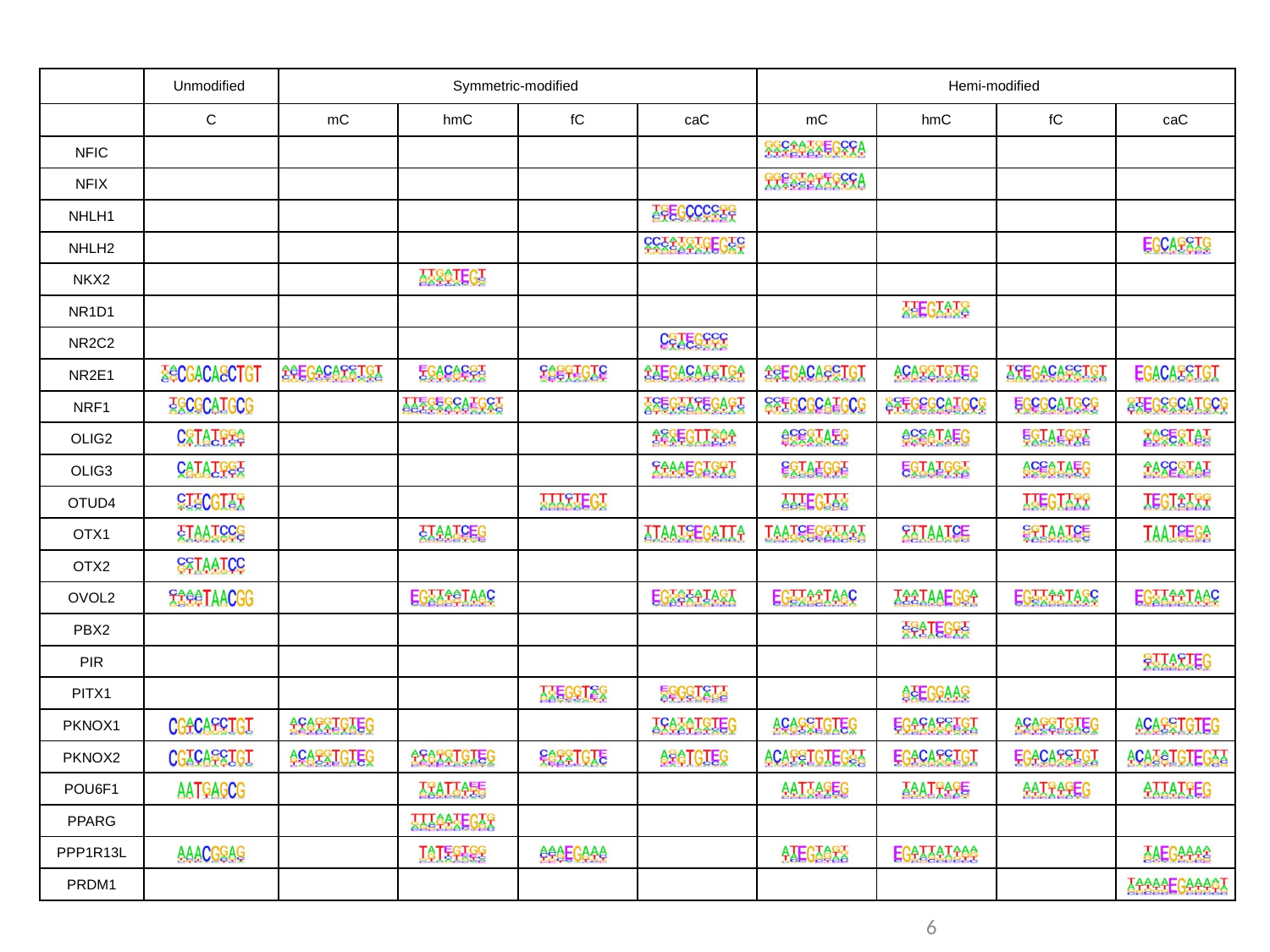

| | Unmodified | Symmetric-modified | | | | Hemi-modified | | | |
| --- | --- | --- | --- | --- | --- | --- | --- | --- | --- |
| | C | mC | hmC | fC | caC | mC | hmC | fC | caC |
| NFIC | | | | | | | | | |
| NFIX | | | | | | | | | |
| NHLH1 | | | | | | | | | |
| NHLH2 | | | | | | | | | |
| NKX2 | | | | | | | | | |
| NR1D1 | | | | | | | | | |
| NR2C2 | | | | | | | | | |
| NR2E1 | | | | | | | | | |
| NRF1 | | | | | | | | | |
| OLIG2 | | | | | | | | | |
| OLIG3 | | | | | | | | | |
| OTUD4 | | | | | | | | | |
| OTX1 | | | | | | | | | |
| OTX2 | | | | | | | | | |
| OVOL2 | | | | | | | | | |
| PBX2 | | | | | | | | | |
| PIR | | | | | | | | | |
| PITX1 | | | | | | | | | |
| PKNOX1 | | | | | | | | | |
| PKNOX2 | | | | | | | | | |
| POU6F1 | | | | | | | | | |
| PPARG | | | | | | | | | |
| PPP1R13L | | | | | | | | | |
| PRDM1 | | | | | | | | | |
6

## Slide 7
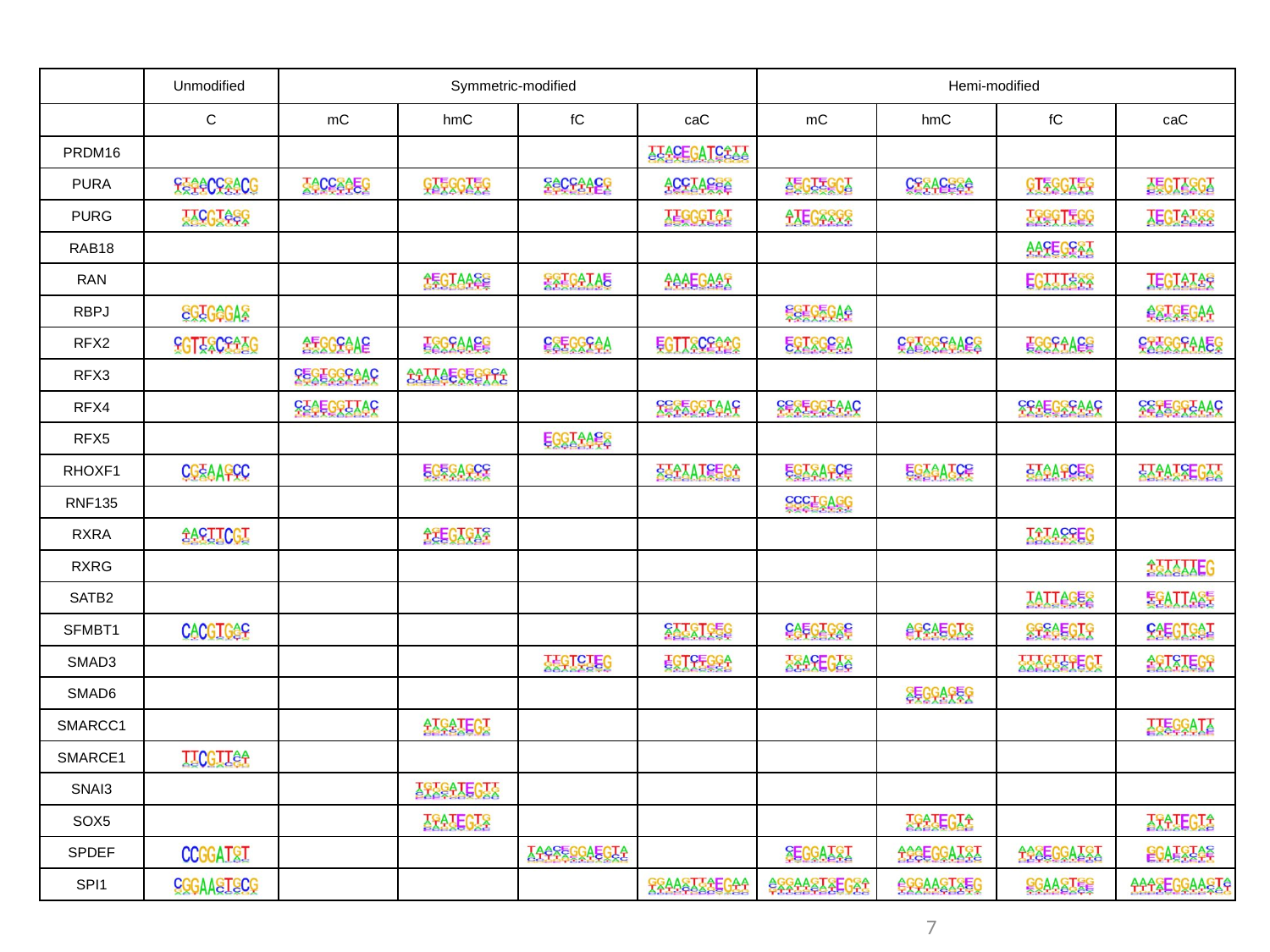

| | Unmodified | Symmetric-modified | | | | Hemi-modified | | | |
| --- | --- | --- | --- | --- | --- | --- | --- | --- | --- |
| | C | mC | hmC | fC | caC | mC | hmC | fC | caC |
| PRDM16 | | | | | | | | | |
| PURA | | | | | | | | | |
| PURG | | | | | | | | | |
| RAB18 | | | | | | | | | |
| RAN | | | | | | | | | |
| RBPJ | | | | | | | | | |
| RFX2 | | | | | | | | | |
| RFX3 | | | | | | | | | |
| RFX4 | | | | | | | | | |
| RFX5 | | | | | | | | | |
| RHOXF1 | | | | | | | | | |
| RNF135 | | | | | | | | | |
| RXRA | | | | | | | | | |
| RXRG | | | | | | | | | |
| SATB2 | | | | | | | | | |
| SFMBT1 | | | | | | | | | |
| SMAD3 | | | | | | | | | |
| SMAD6 | | | | | | | | | |
| SMARCC1 | | | | | | | | | |
| SMARCE1 | | | | | | | | | |
| SNAI3 | | | | | | | | | |
| SOX5 | | | | | | | | | |
| SPDEF | | | | | | | | | |
| SPI1 | | | | | | | | | |
7

## Slide 8
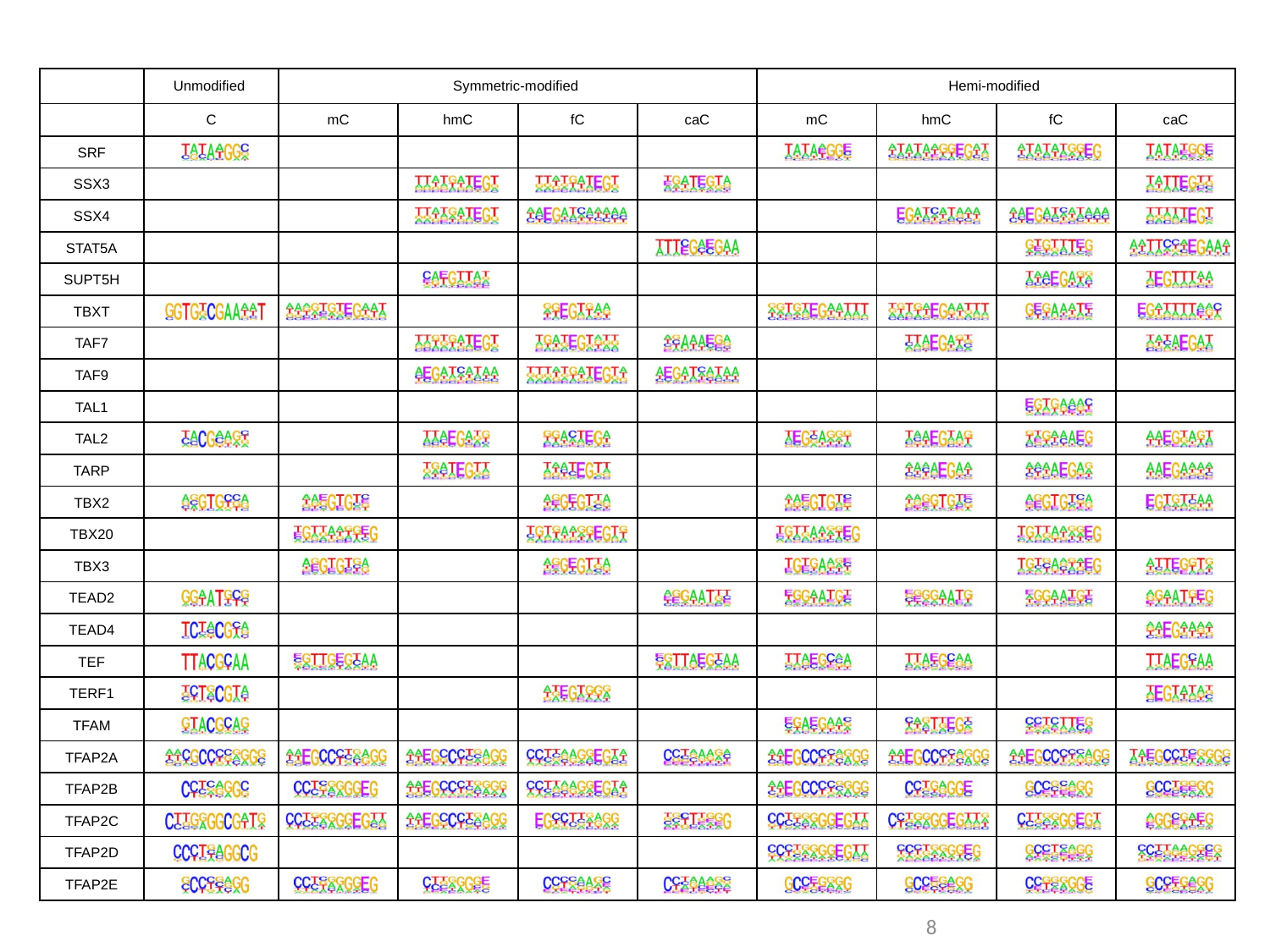

| | Unmodified | Symmetric-modified | | | | Hemi-modified | | | |
| --- | --- | --- | --- | --- | --- | --- | --- | --- | --- |
| | C | mC | hmC | fC | caC | mC | hmC | fC | caC |
| SRF | | | | | | | | | |
| SSX3 | | | | | | | | | |
| SSX4 | | | | | | | | | |
| STAT5A | | | | | | | | | |
| SUPT5H | | | | | | | | | |
| TBXT | | | | | | | | | |
| TAF7 | | | | | | | | | |
| TAF9 | | | | | | | | | |
| TAL1 | | | | | | | | | |
| TAL2 | | | | | | | | | |
| TARP | | | | | | | | | |
| TBX2 | | | | | | | | | |
| TBX20 | | | | | | | | | |
| TBX3 | | | | | | | | | |
| TEAD2 | | | | | | | | | |
| TEAD4 | | | | | | | | | |
| TEF | | | | | | | | | |
| TERF1 | | | | | | | | | |
| TFAM | | | | | | | | | |
| TFAP2A | | | | | | | | | |
| TFAP2B | | | | | | | | | |
| TFAP2C | | | | | | | | | |
| TFAP2D | | | | | | | | | |
| TFAP2E | | | | | | | | | |
8

## Slide 9
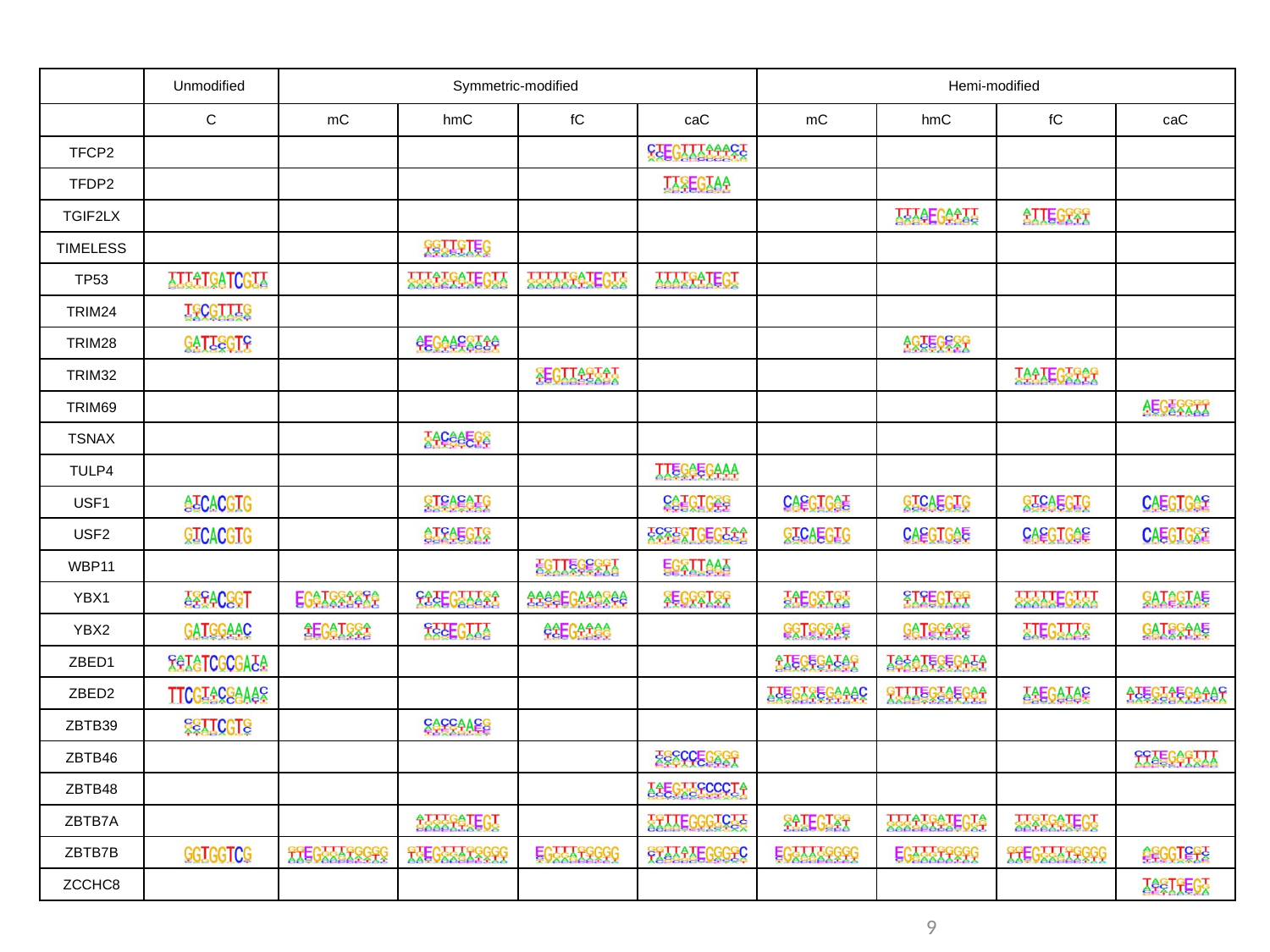

| | Unmodified | Symmetric-modified | | | | Hemi-modified | | | |
| --- | --- | --- | --- | --- | --- | --- | --- | --- | --- |
| | C | mC | hmC | fC | caC | mC | hmC | fC | caC |
| TFCP2 | | | | | | | | | |
| TFDP2 | | | | | | | | | |
| TGIF2LX | | | | | | | | | |
| TIMELESS | | | | | | | | | |
| TP53 | | | | | | | | | |
| TRIM24 | | | | | | | | | |
| TRIM28 | | | | | | | | | |
| TRIM32 | | | | | | | | | |
| TRIM69 | | | | | | | | | |
| TSNAX | | | | | | | | | |
| TULP4 | | | | | | | | | |
| USF1 | | | | | | | | | |
| USF2 | | | | | | | | | |
| WBP11 | | | | | | | | | |
| YBX1 | | | | | | | | | |
| YBX2 | | | | | | | | | |
| ZBED1 | | | | | | | | | |
| ZBED2 | | | | | | | | | |
| ZBTB39 | | | | | | | | | |
| ZBTB46 | | | | | | | | | |
| ZBTB48 | | | | | | | | | |
| ZBTB7A | | | | | | | | | |
| ZBTB7B | | | | | | | | | |
| ZCCHC8 | | | | | | | | | |
9

## Slide 10
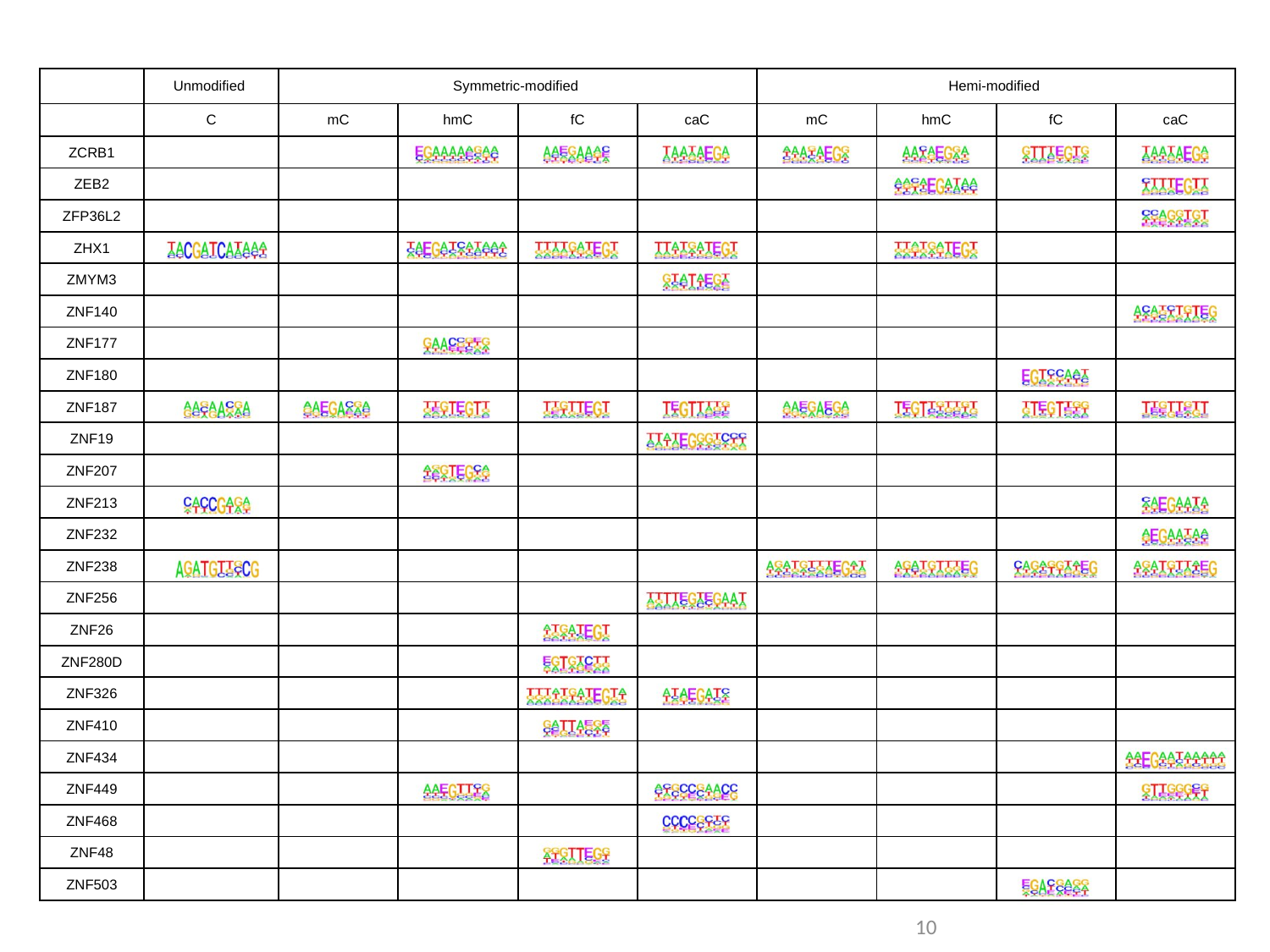

| | Unmodified | Symmetric-modified | | | | Hemi-modified | | | |
| --- | --- | --- | --- | --- | --- | --- | --- | --- | --- |
| | C | mC | hmC | fC | caC | mC | hmC | fC | caC |
| ZCRB1 | | | | | | | | | |
| ZEB2 | | | | | | | | | |
| ZFP36L2 | | | | | | | | | |
| ZHX1 | | | | | | | | | |
| ZMYM3 | | | | | | | | | |
| ZNF140 | | | | | | | | | |
| ZNF177 | | | | | | | | | |
| ZNF180 | | | | | | | | | |
| ZNF187 | | | | | | | | | |
| ZNF19 | | | | | | | | | |
| ZNF207 | | | | | | | | | |
| ZNF213 | | | | | | | | | |
| ZNF232 | | | | | | | | | |
| ZNF238 | | | | | | | | | |
| ZNF256 | | | | | | | | | |
| ZNF26 | | | | | | | | | |
| ZNF280D | | | | | | | | | |
| ZNF326 | | | | | | | | | |
| ZNF410 | | | | | | | | | |
| ZNF434 | | | | | | | | | |
| ZNF449 | | | | | | | | | |
| ZNF468 | | | | | | | | | |
| ZNF48 | | | | | | | | | |
| ZNF503 | | | | | | | | | |
10

## Slide 11
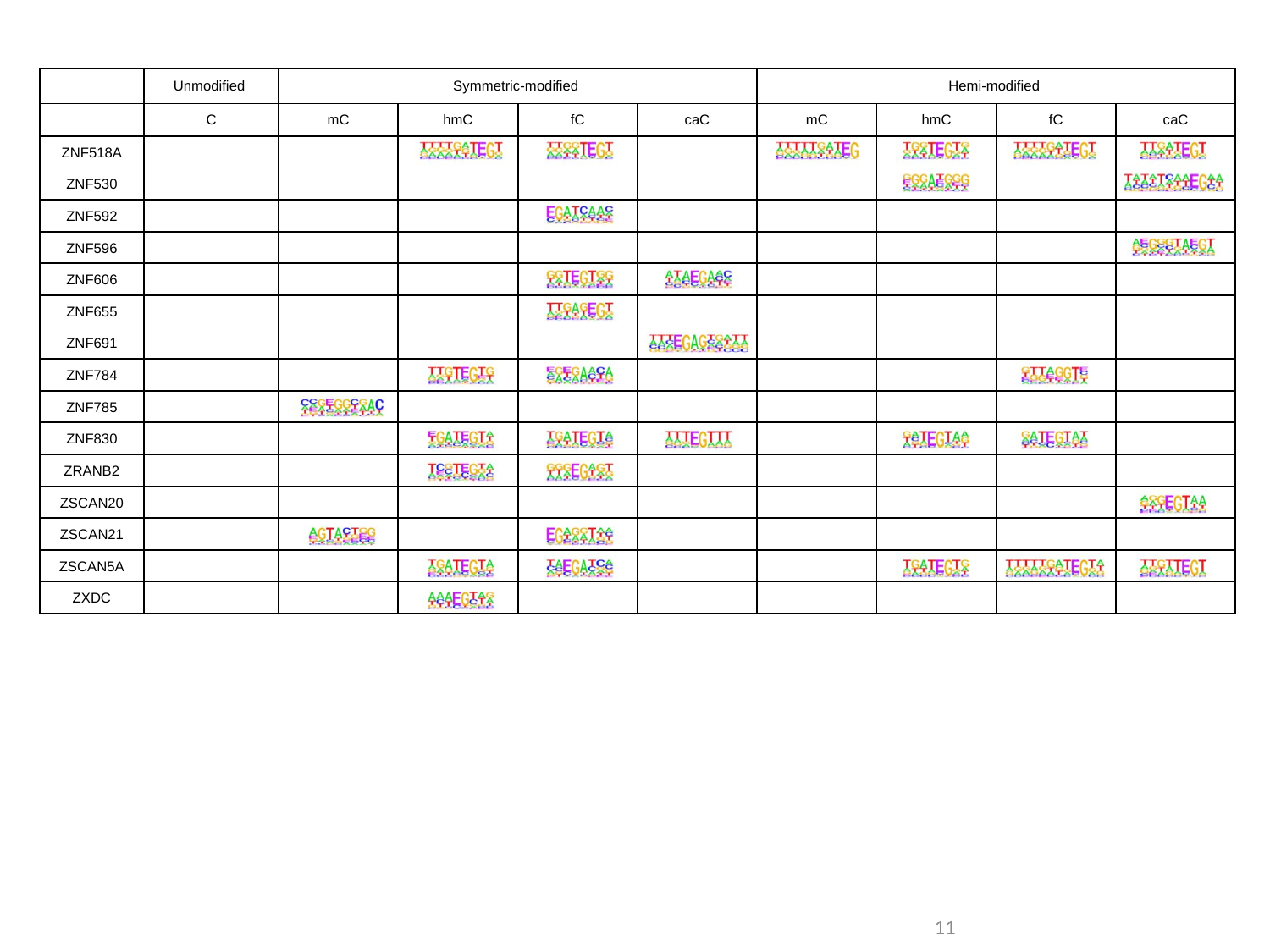

| | Unmodified | Symmetric-modified | | | | Hemi-modified | | | |
| --- | --- | --- | --- | --- | --- | --- | --- | --- | --- |
| | C | mC | hmC | fC | caC | mC | hmC | fC | caC |
| ZNF518A | | | | | | | | | |
| ZNF530 | | | | | | | | | |
| ZNF592 | | | | | | | | | |
| ZNF596 | | | | | | | | | |
| ZNF606 | | | | | | | | | |
| ZNF655 | | | | | | | | | |
| ZNF691 | | | | | | | | | |
| ZNF784 | | | | | | | | | |
| ZNF785 | | | | | | | | | |
| ZNF830 | | | | | | | | | |
| ZRANB2 | | | | | | | | | |
| ZSCAN20 | | | | | | | | | |
| ZSCAN21 | | | | | | | | | |
| ZSCAN5A | | | | | | | | | |
| ZXDC | | | | | | | | | |
11
