# Supplementary material for: An all-to-all approach to the identification of sequence-specific readers for epigenetic DNA modifications on cytosine
Source: Nat Commun. 2021 Feb 4;12:795. doi: 10.1038/s41467-021-20950-w (PMC7862700; doi:10.1038/s41467-021-20950-w)

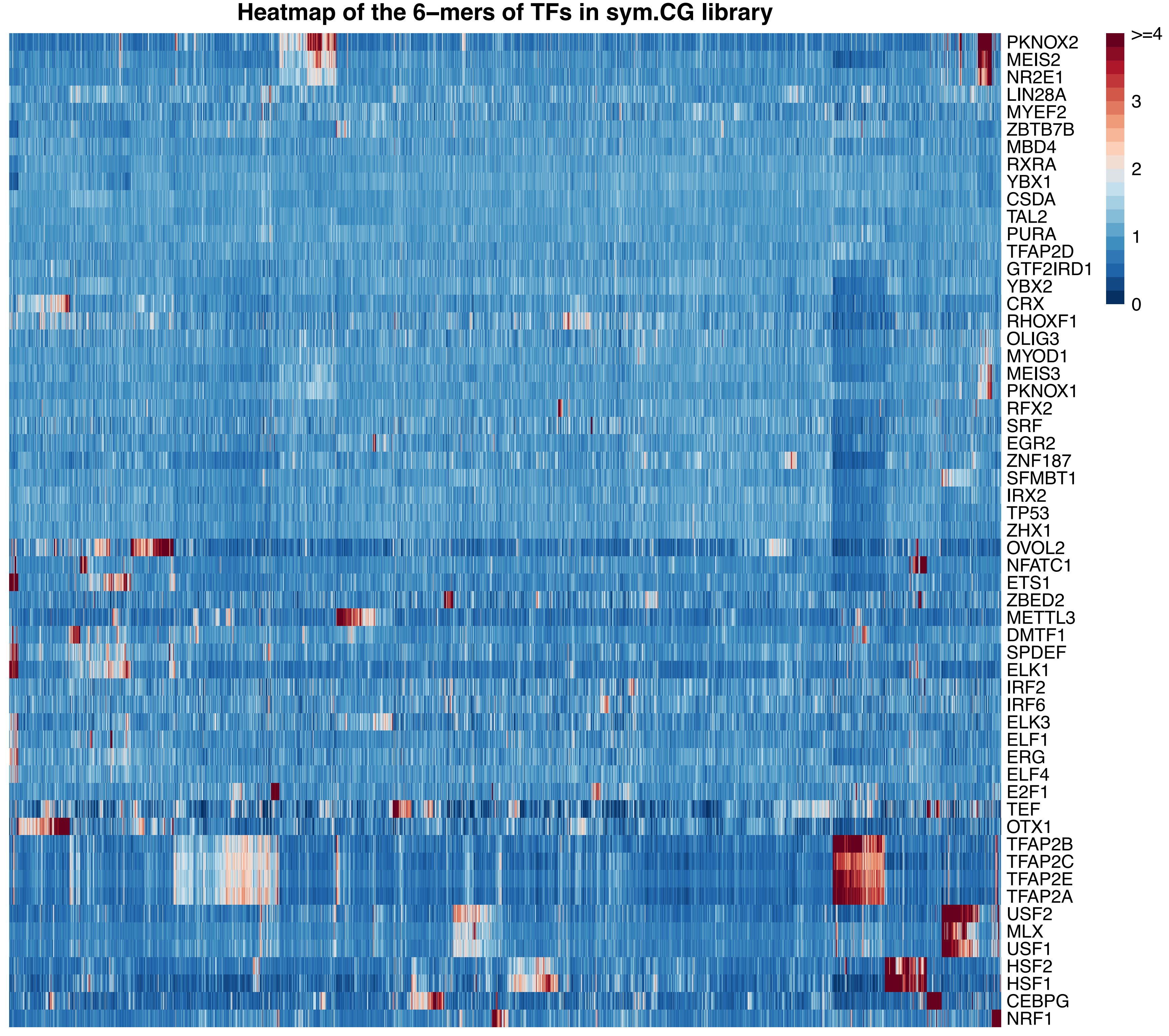
Heatmap of the 6–mers of TFs in sym.CG library
PKNOX2
MEIS2
NR2E1
LIN28A
MYEF2
ZBTB7B
MBD4
RXRA
YBX1
CSDA
TAL2
PURA
TFAP2D
GTF2IRD1
YBX2
CRX
RHOXF1
OLIG3
MYOD1
MEIS3
PKNOX1
RFX2
SRF
EGR2
ZNF187
SFMBT1
IRX2
TP53
ZHX1
OVOL2
NFATC1
ETS1
ZBED2
METTL3
DMTF1
SPDEF
ELK1
IRF2
IRF6
ELK3
ELF1
ERG
ELF4
E2F1
TEF
OTX1
TFAP2B
TFAP2C
TFAP2E
TFAP2A
USF2
MLX
USF1
HSF2
HSF1
CEBPG
NRF1
>=4
3
2
1
0

Heatmap of the 6−mers of TFs in sym.mCG library

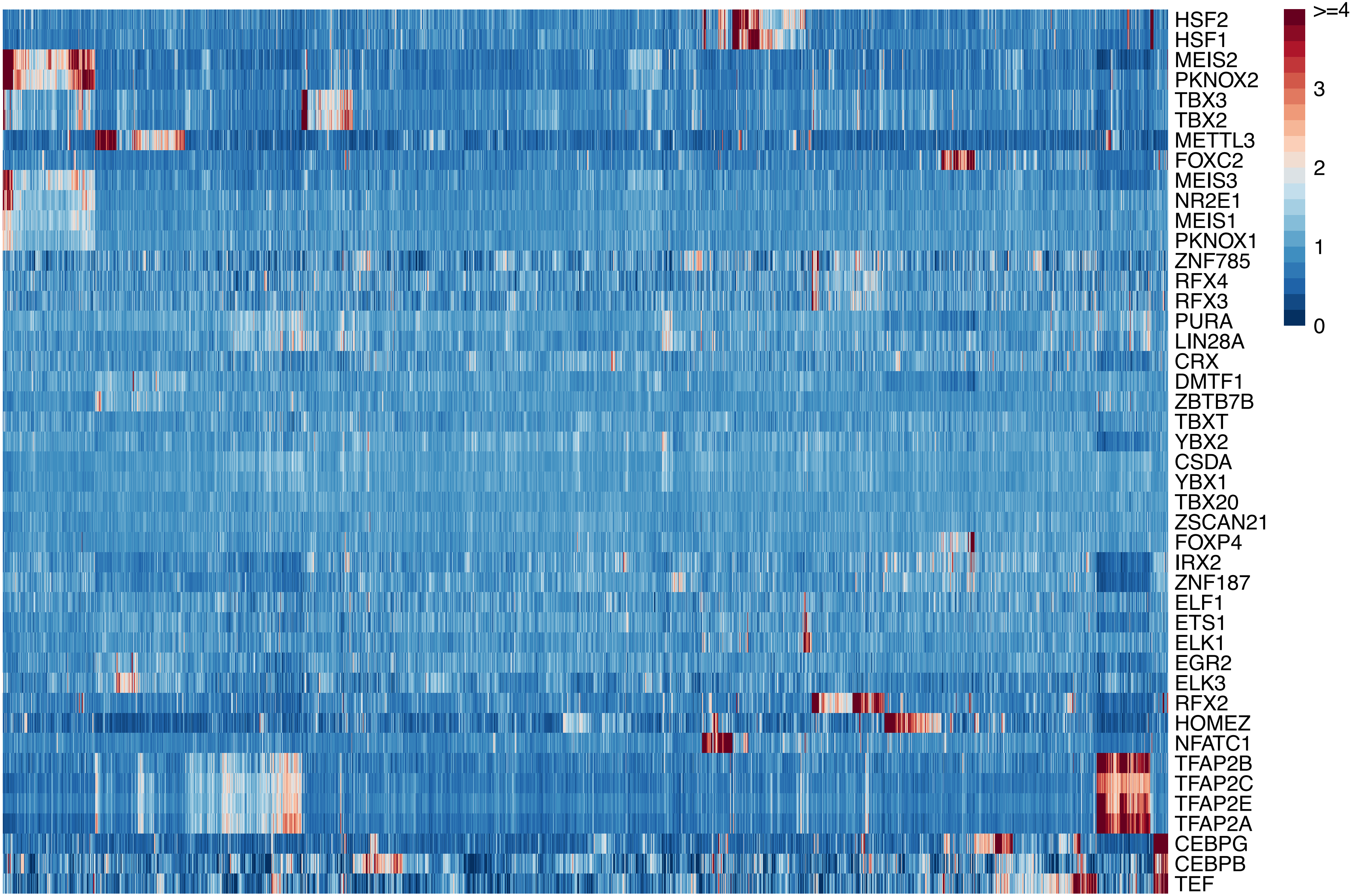

Heatmap of the 6−mers of TFs in sym.hmCG library

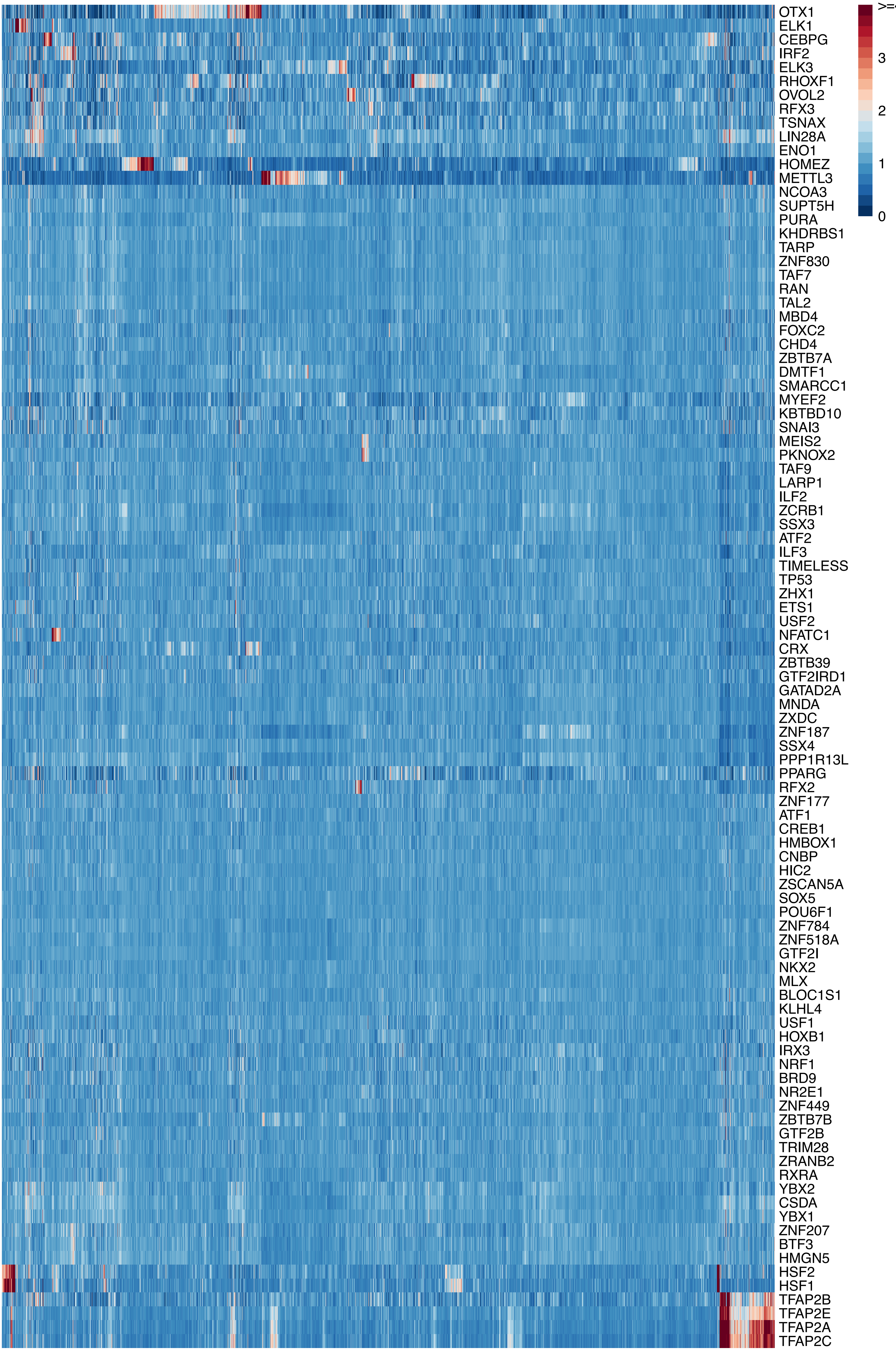

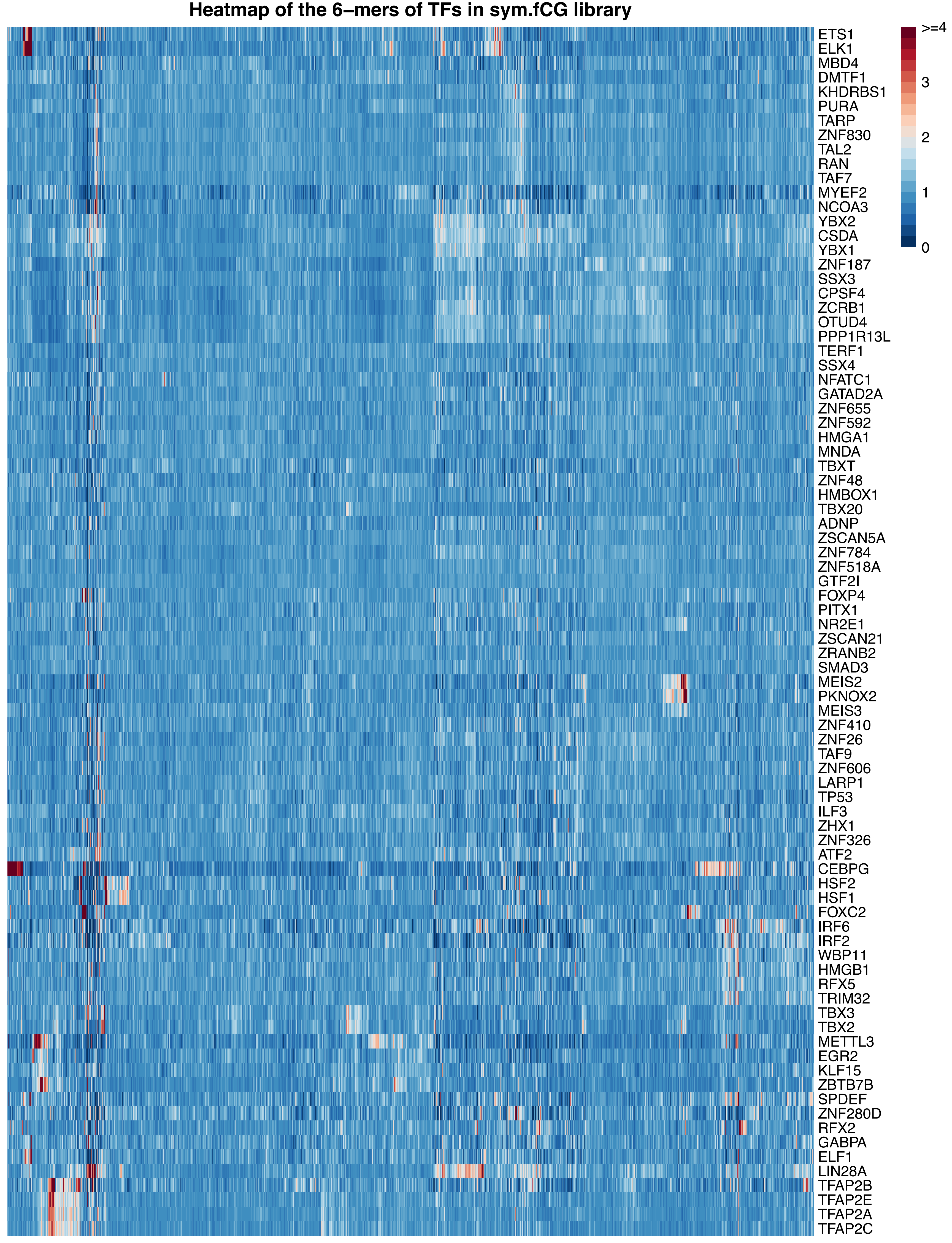
Heatmap of the 6−mers of TFs in sym.fCG library
ETS1
ELK1
MBD4
DMTF1
KHDRBS1
PURA
TARP
ZNF830
TAL2
RAN
TAF7
MYEF2
NCOA3
YBX2
CSDA
YBX1
ZNF187
SSX3
CPSF4
ZCRB1
OTUD4
PPP1R13L
TERF1
SSX4
NFATC1
GATAD2A
ZNF655
ZNF592
HMGA1
MNDA
TBXT
ZNF48
HMBOX1
TBX20
ADNP
ZSCAN5A
ZNF784
ZNF518A
GTF2I
FOXP4
PITX1
NR2E1
ZSCAN21
ZRANB2
SMAD3
MEIS2
PKNOX2
MEIS3
ZNF410
ZNF26
TAF9
ZNF606
LARP1
TP53
ILF3
ZHX1
ZNF326
ATF2
CEBPG
HSF2
HSF1
FOXC2
IRF6
IRF2
WBP11
HMGB1
RFX5
TRIM32
TBX3
TBX2
METTL3
EGR2
KLF15
ZBTB7B
SPDEF
ZNF280D
RFX2
GABPA
ELF1
LIN28A
TFAP2B
TFAP2E
TFAP2A
TFAP2C
>=4
3
2
1
0

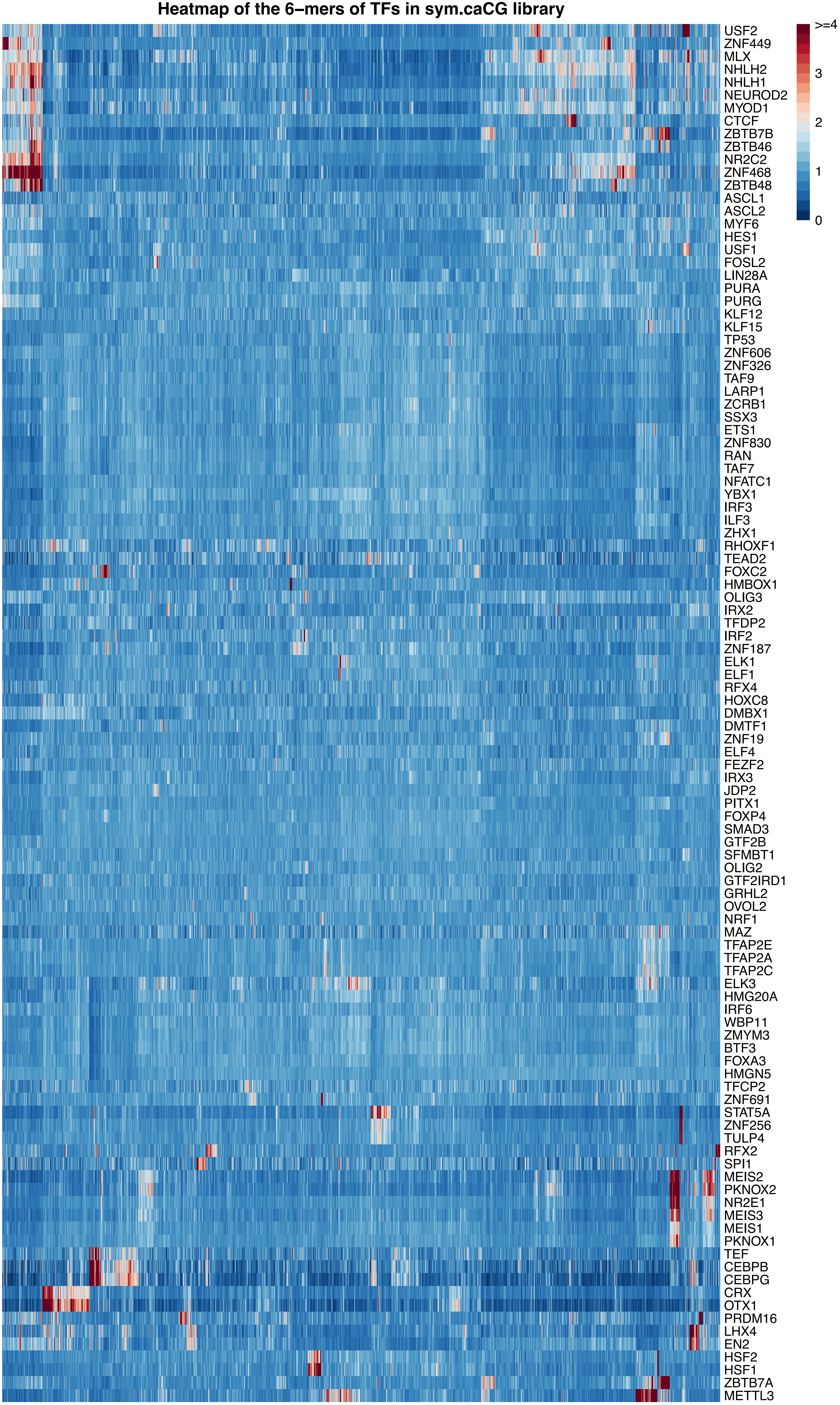
Heatmap of the 6−mers of TFs in sym.caCG library
>=4
3
2
1
0
USF2
ZNF449
MLX
NHLH2
NHLH1
NEUROD2
MYOD1
CTCF
ZBTB7B
ZBTB46
NR2C2
ZNF468
ZBTB48
ASCL1
ASCL2
MYF6
HES1
USF1
FOSL2
LIN28A
PURA
PURG
KLF12
KLF15
TP53
ZNF606
ZNF326
TAF9
LARP1
ZCRB1
SSX3
ETS1
ZNF830
RAN
TAF7
NFATC1
YBX1
IRF3
ILF3
ZHX1
RHOXF1
TEAD2
FOXC2
HMBOX1
OLIG3
IRX2
TFDP2
IRF2
ZNF187
ELK1
ELF1
RFX4
HOXC8
DMBX1
DMTF1
ZNF19
ELF4
FEZF2
IRX3
JDP2
PITX1
FOXP4
SMAD3
GTF2B
SFMBT1
OLIG2
GTF2IRD1
GRHL2
OVOL2
NRF1
MAZ
TFAP2E
TFAP2A
TFAP2C
ELK3
HMG20A
IRF6
WBP11
ZMYM3
BTF3
FOXA3
HMGN5
TFCP2
ZNF691
STAT5A
ZNF256
TULP4
RFX2
SPI1
MEIS2
PKNOX2
NR2E1
MEIS3
MEIS1
PKNOX1
TEF
CEBPB
CEBPG
CRX
OTX1
PRDM16
LHX4
EN2
HSF2
HSF1
ZBTB7A
METTL3

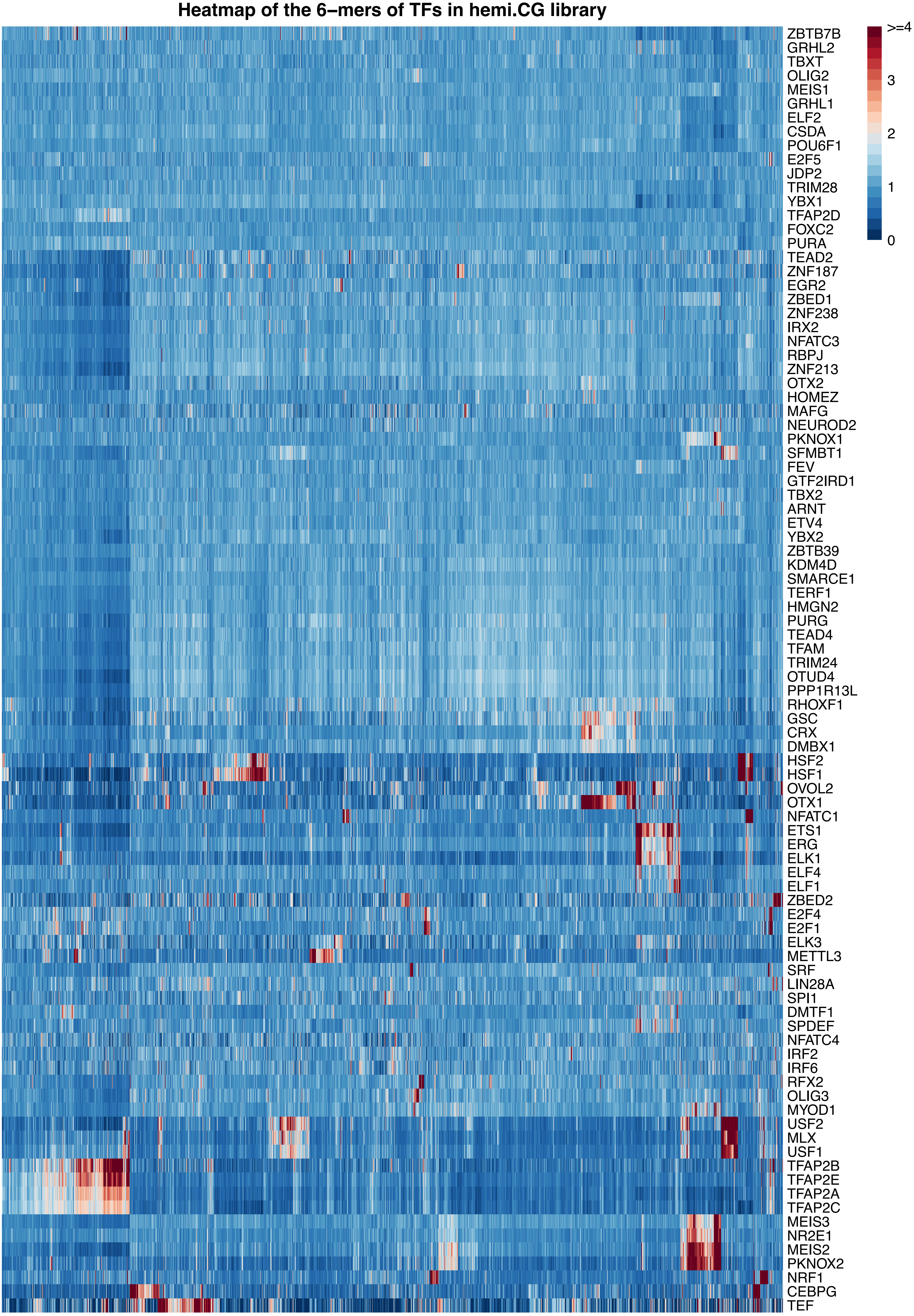
Heatmap of the 6−mers of TFs in hemi.CG library
>=4
3
2
1
0
ZBTB7B
GRHL2
TBXT
OLIG2
MEIS1
GRHL1
ELF2
CSDA
POU6F1
E2F5
JDP2
TRIM28
YBX1
TFAP2D
FOXC2
PURA
TEAD2
ZNF187
EGR2
ZBED1
ZNF238
IRX2
NFATC3
RBPJ
ZNF213
OTX2
HOMEZ
MAFG
NEUROD2
PKNOX1
SFMBT1
FEV
GTF2IRD1
TBX2
ARNT
ETV4
YBX2
ZBTB39
KDM4D
SMARCE1
TERF1
HMGN2
PURG
TEAD4
TFAM
TRIM24
OTUD4
PPP1R13L
RHOXF1
GSC
CRX
DMBX1
HSF2
HSF1
OVOL2
OTX1
NFATC1
ETS1
ERG
ELK1
ELF4
ELF1
ZBED2
E2F4
E2F1
ELK3
METTL3
SRF
LIN28A
SPI1
DMTF1
SPDEF
NFATC4
IRF2
IRF6
RFX2
OLIG3
MYOD1
USF2
MLX
USF1
TFAP2B
TFAP2E
TFAP2A
TFAP2C
MEIS3
NR2E1
MEIS2
PKNOX2
NRF1
CEBPG
TEF

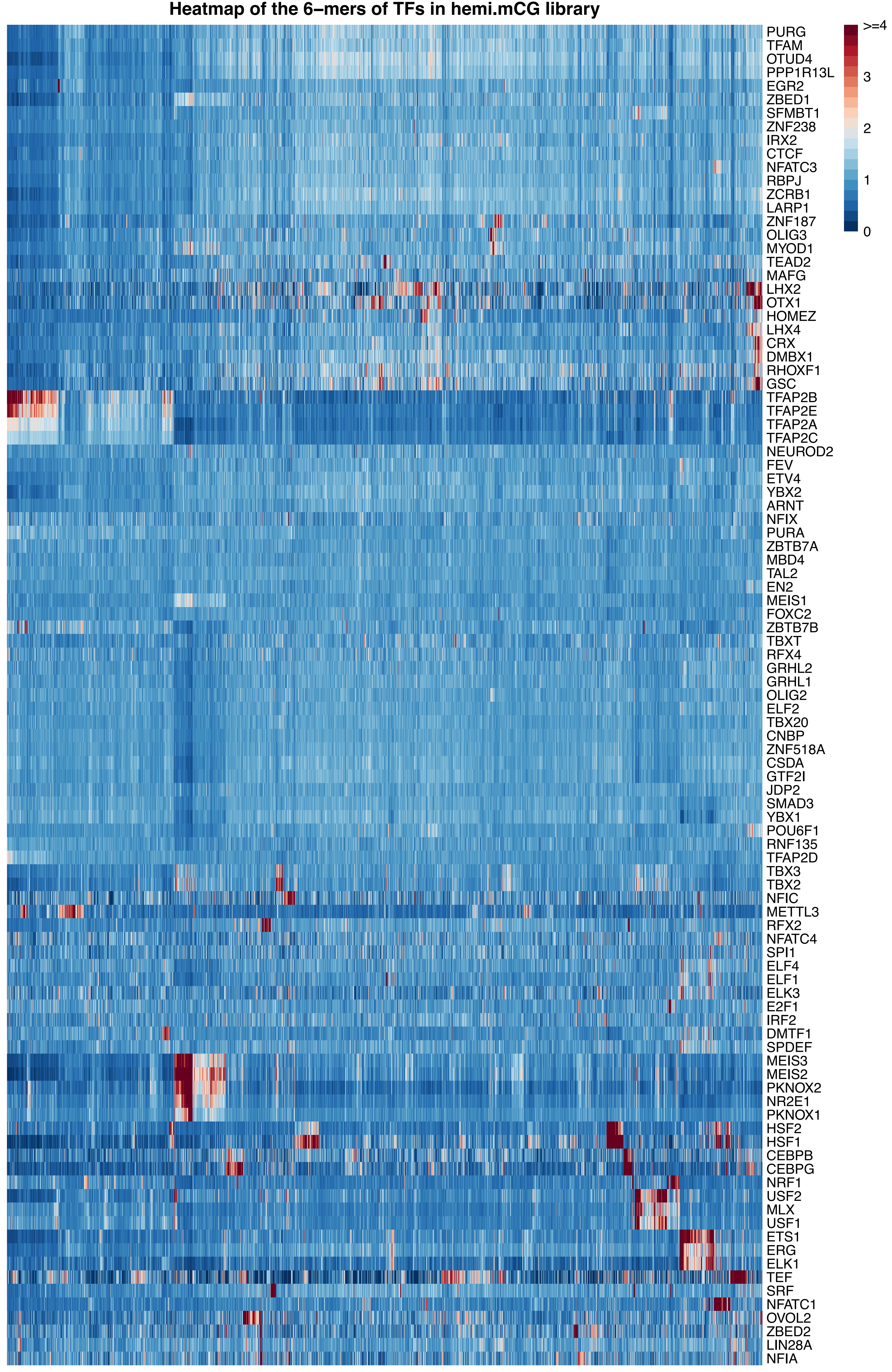
Heatmap of the 6−mers of TFs in hemi.mCG library
>=4
3
2
1
0
PURG
TFAM
OTUD4
PPP1R13L
EGR2
ZBED1
SFMBT1
ZNF238
IRX2
CTCF
NFATC3
RBPJ
ZCRB1
LARP1
ZNF187
OLIG3
MYOD1
TEAD2
MAFG
LHX2
OTX1
HOMEZ
LHX4
CRX
DMBX1
RHOXF1
GSC
TFAP2B
TFAP2E
TFAP2A
TFAP2C
NEUROD2
FEV
ETV4
YBX2
ARNT
NFIX
PURA
ZBTB7A
MBD4
TAL2
EN2
MEIS1
FOXC2
ZBTB7B
TBXT
RFX4
GRHL2
GRHL1
OLIG2
ELF2
TBX20
CNBP
ZNF518A
CSDA
GTF2I
JDP2
SMAD3
YBX1
POU6F1
RNF135
TFAP2D
TBX3
TBX2
NFIC
METTL3
RFX2
NFATC4
SPI1
ELF4
ELF1
ELK3
E2F1
IRF2
DMTF1
SPDEF
MEIS3
MEIS2
PKNOX2
NR2E1
PKNOX1
HSF2
HSF1
CEBPB
CEBPG
NRF1
USF2
MLX
USF1
ETS1
ERG
ELK1
TEF
SRF
NFATC1
OVOL2
ZBED2
LIN28A
NFIA

Heatmap of the 6−mers of TFs in hemi.hmCG library

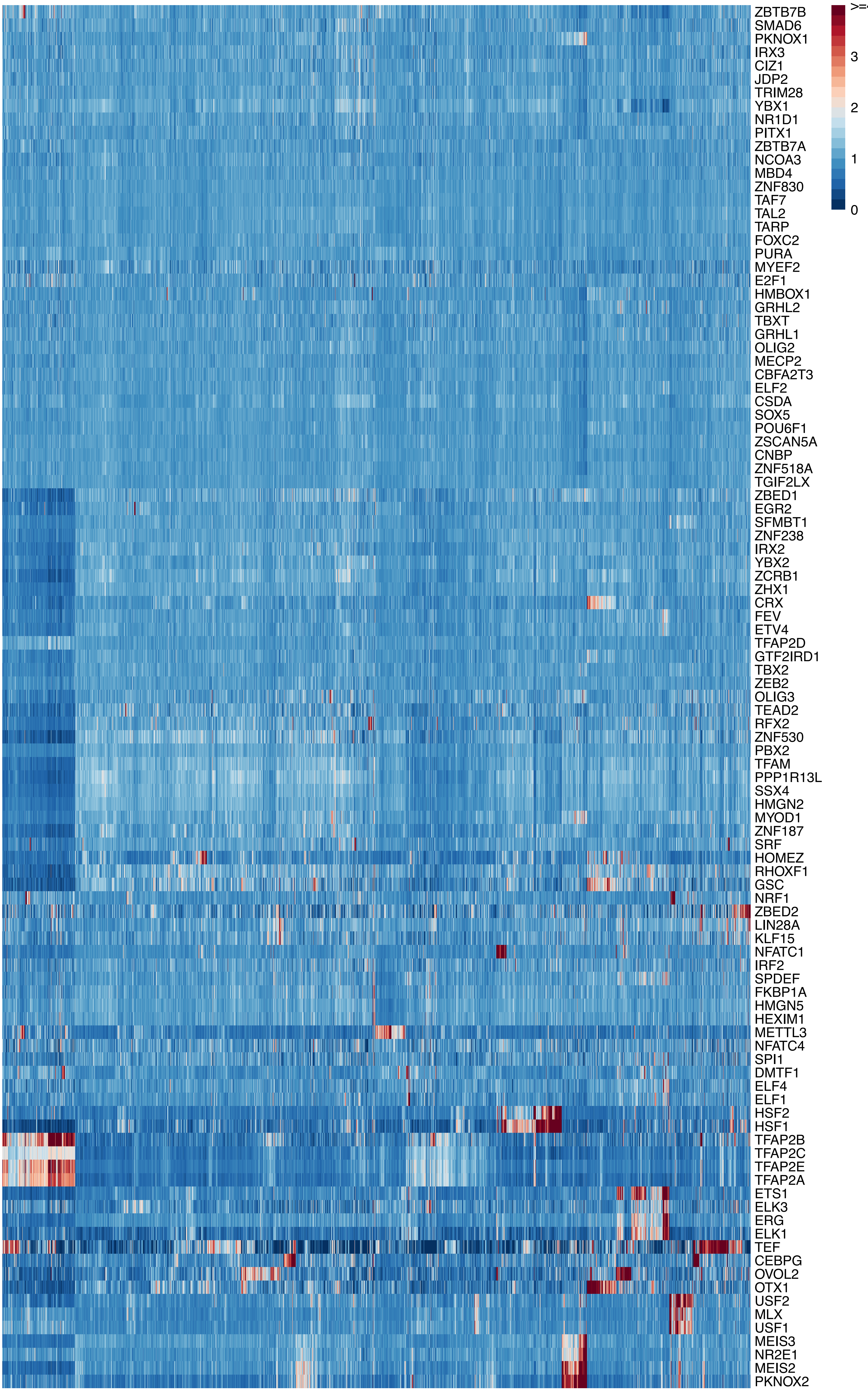

Heatmap of the 6−mers of TFs in hemi.fCG library

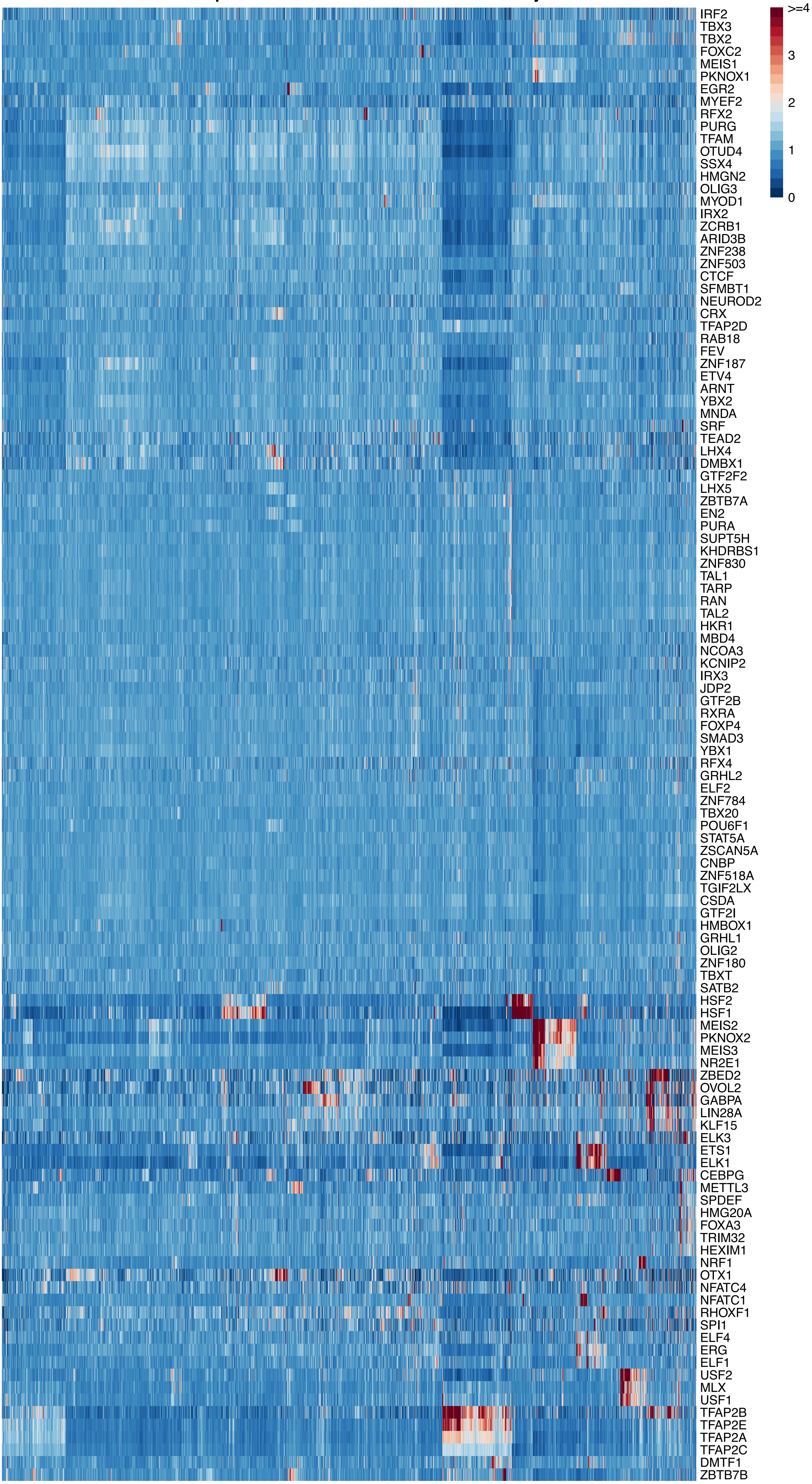

Heatmap of the 6–mers of TFs in hemi.caCG library

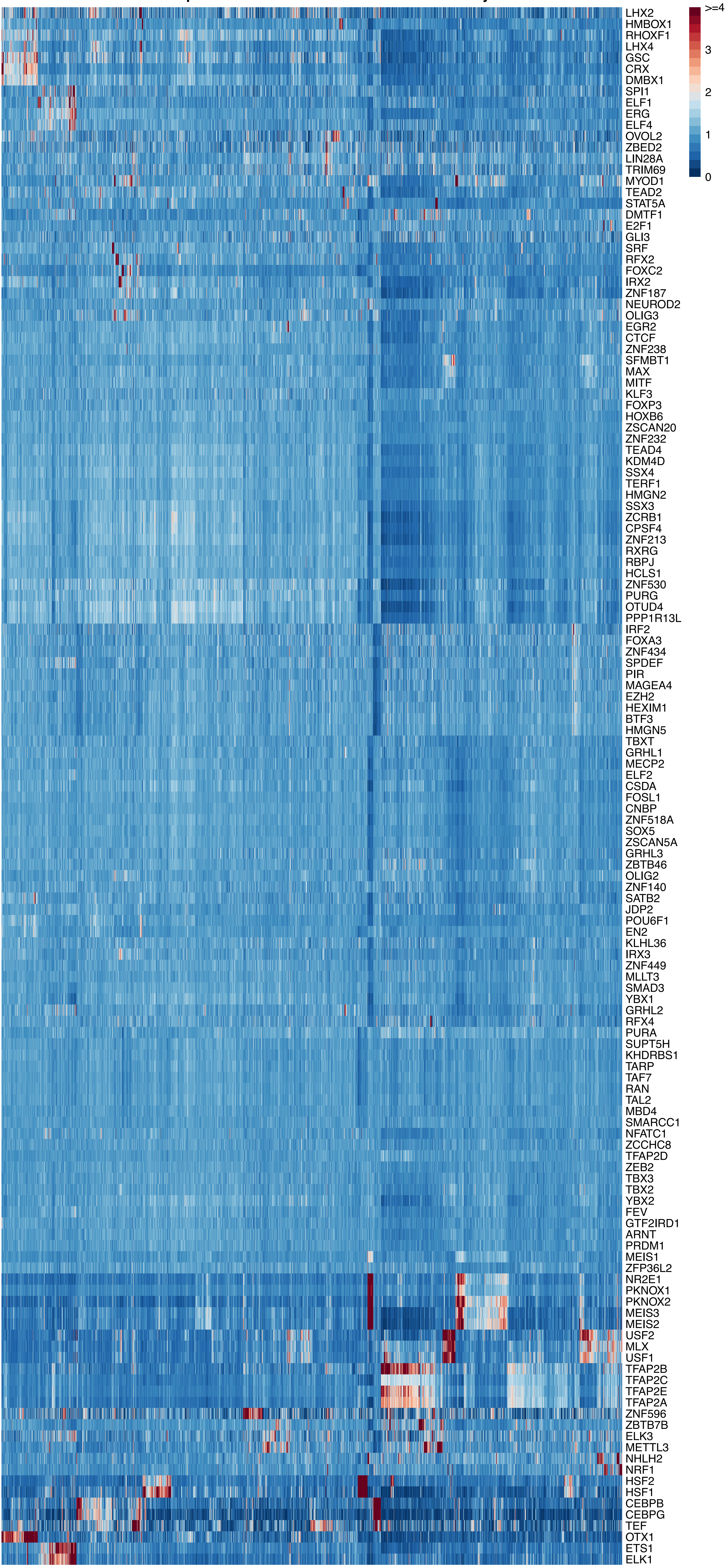

Supplement: Supplementary file 9 — Supplementary Data 6 [file 41467_2021_20950_MOESM9_ESM.pdf]
