# Supplementary material for: An all-to-all approach to the identification of sequence-specific readers for epigenetic DNA modifications on cytosine
Source: Nat Commun. 2021 Feb 4;12:795. doi: 10.1038/s41467-021-20950-w (PMC7862700; doi:10.1038/s41467-021-20950-w)
Supplement: Supplementary file 10 — Supplementary Data 7 [file 41467_2021_20950_MOESM10_ESM.pptx]

## Slide 1
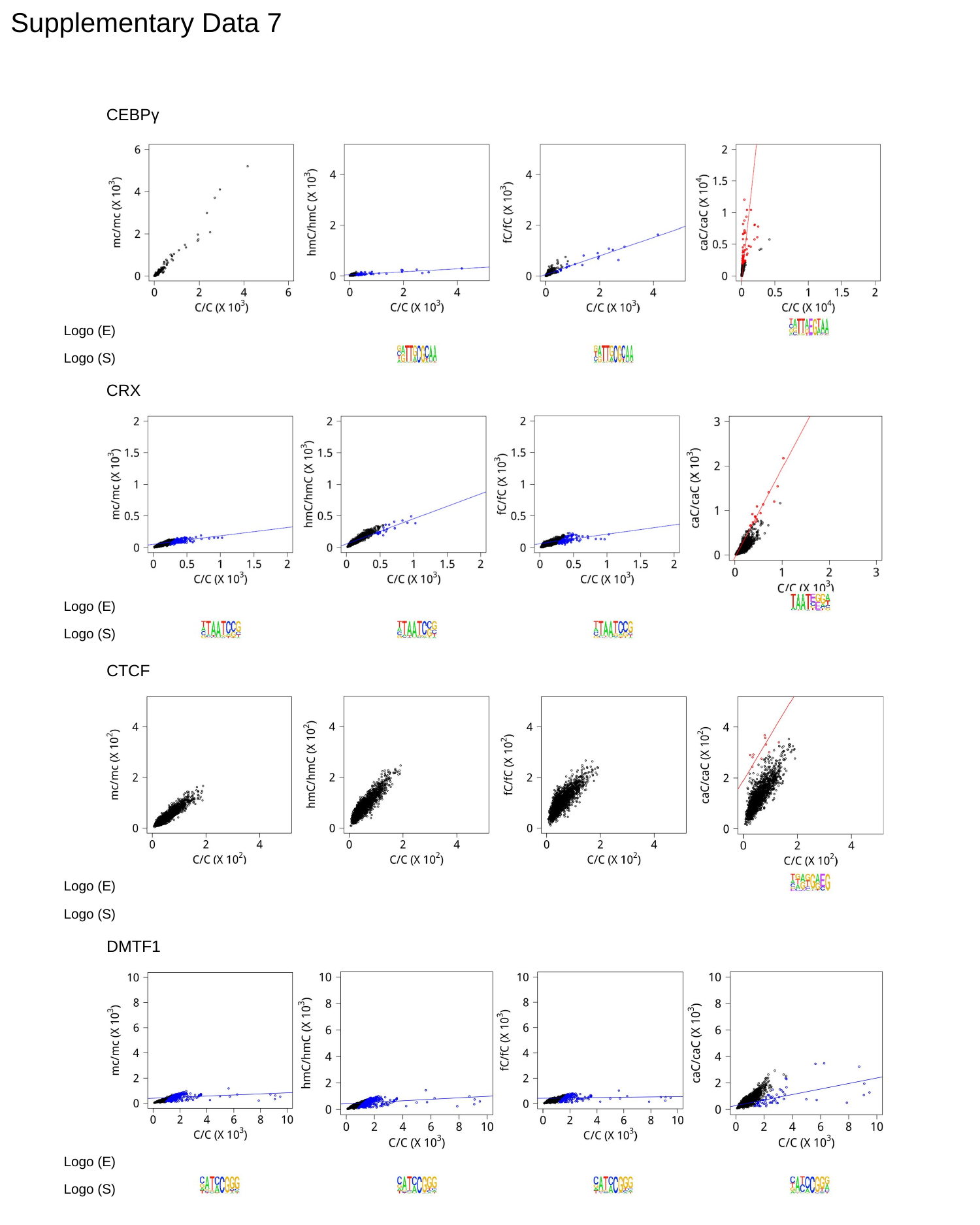

Supplementary Data 7
CEBPγ
Logo (E)
Logo (S)
CRX
Logo (E)
Logo (S)
CTCF
Logo (E)
Logo (S)
DMTF1
Logo (E)
Logo (S)

## Slide 2
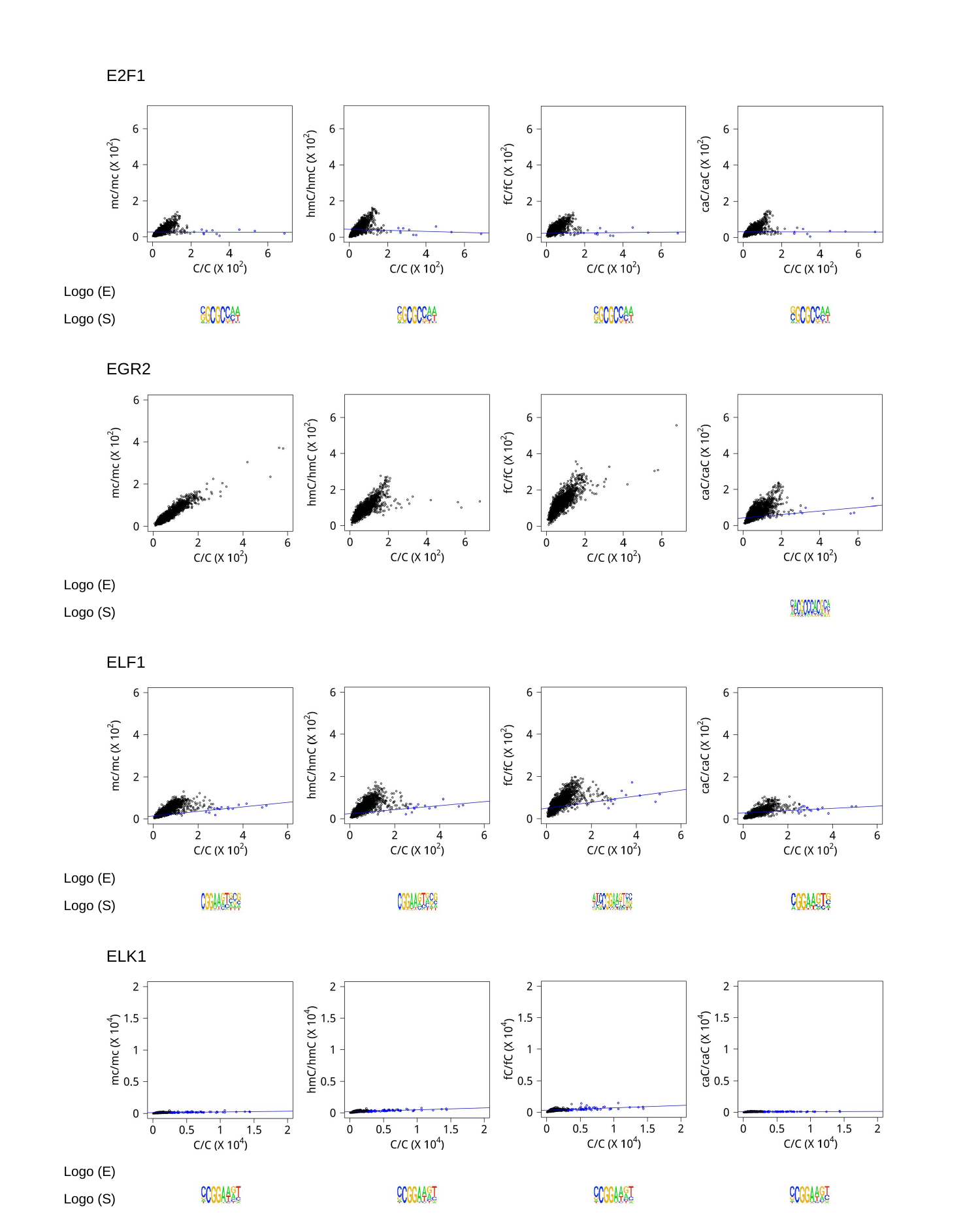

E2F1
Logo (E)
Logo (S)
EGR2
Logo (E)
Logo (S)
ELF1
Logo (E)
Logo (S)
ELK1
Logo (E)
Logo (S)

## Slide 3
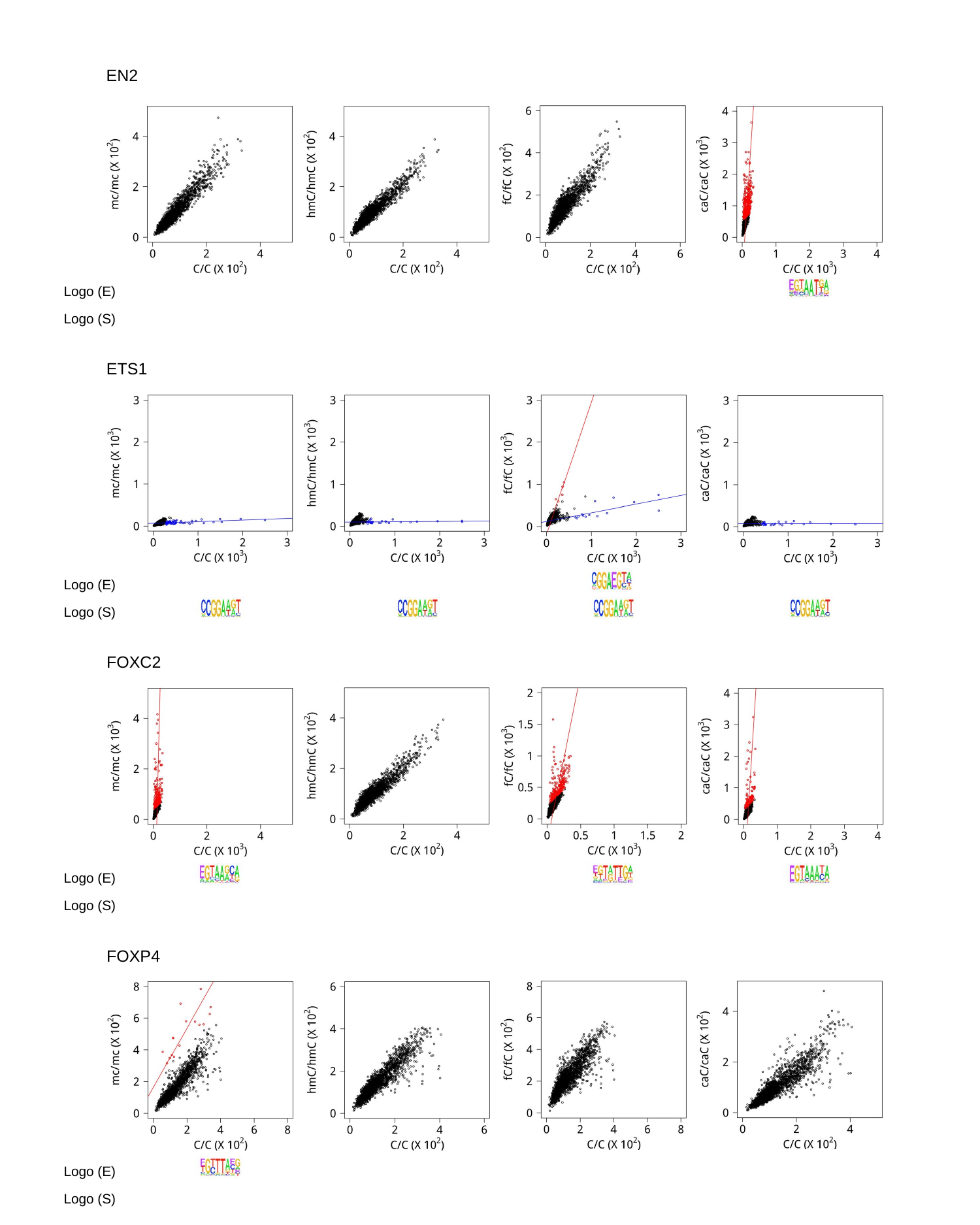

EN2
Logo (E)
Logo (S)
ETS1
Logo (E)
Logo (S)
FOXC2
Logo (E)
Logo (S)
FOXP4
Logo (E)
Logo (S)

## Slide 4
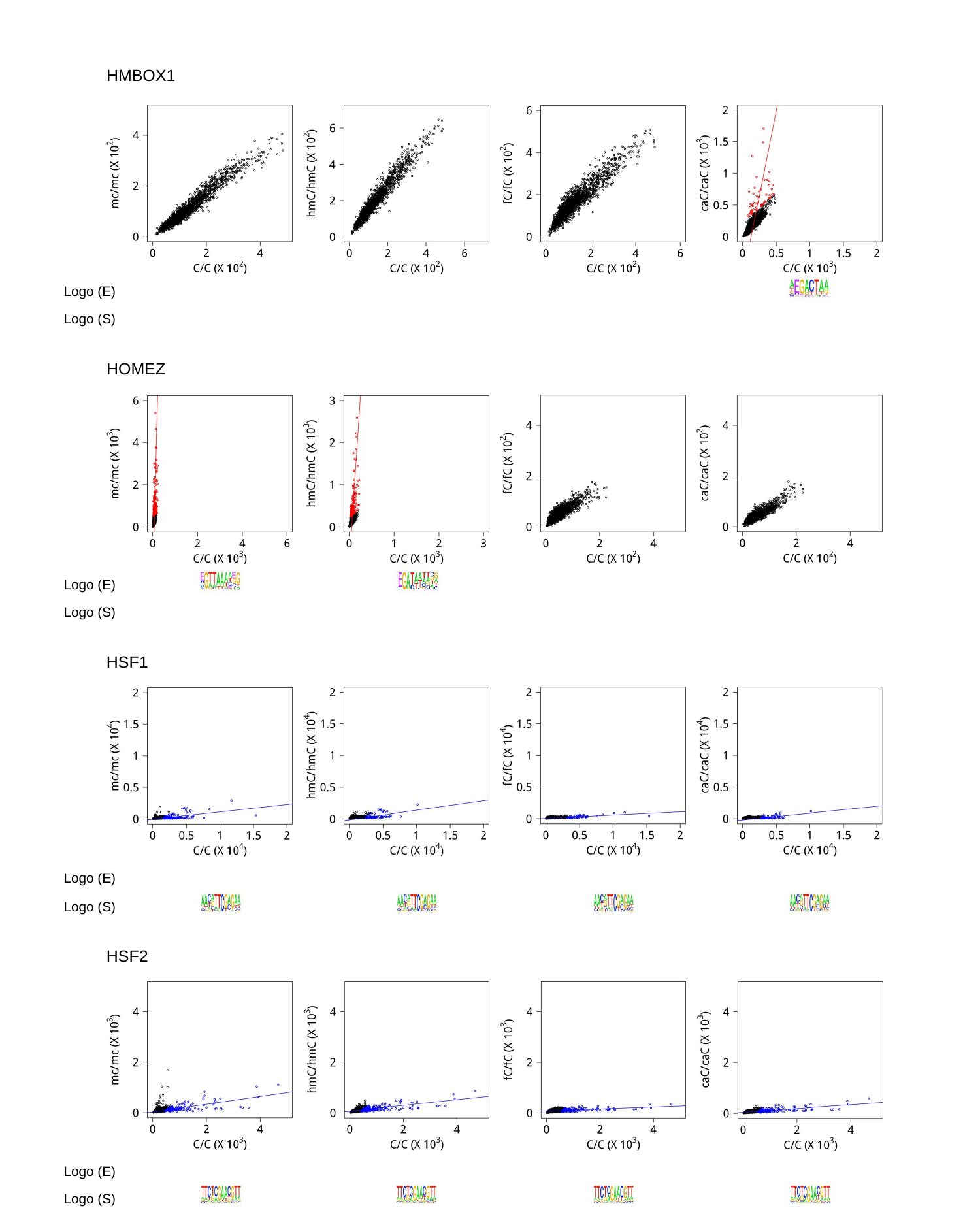

HMBOX1
Logo (E)
Logo (S)
HOMEZ
Logo (E)
Logo (S)
HSF1
Logo (E)
Logo (S)
HSF2
Logo (E)
Logo (S)

## Slide 5
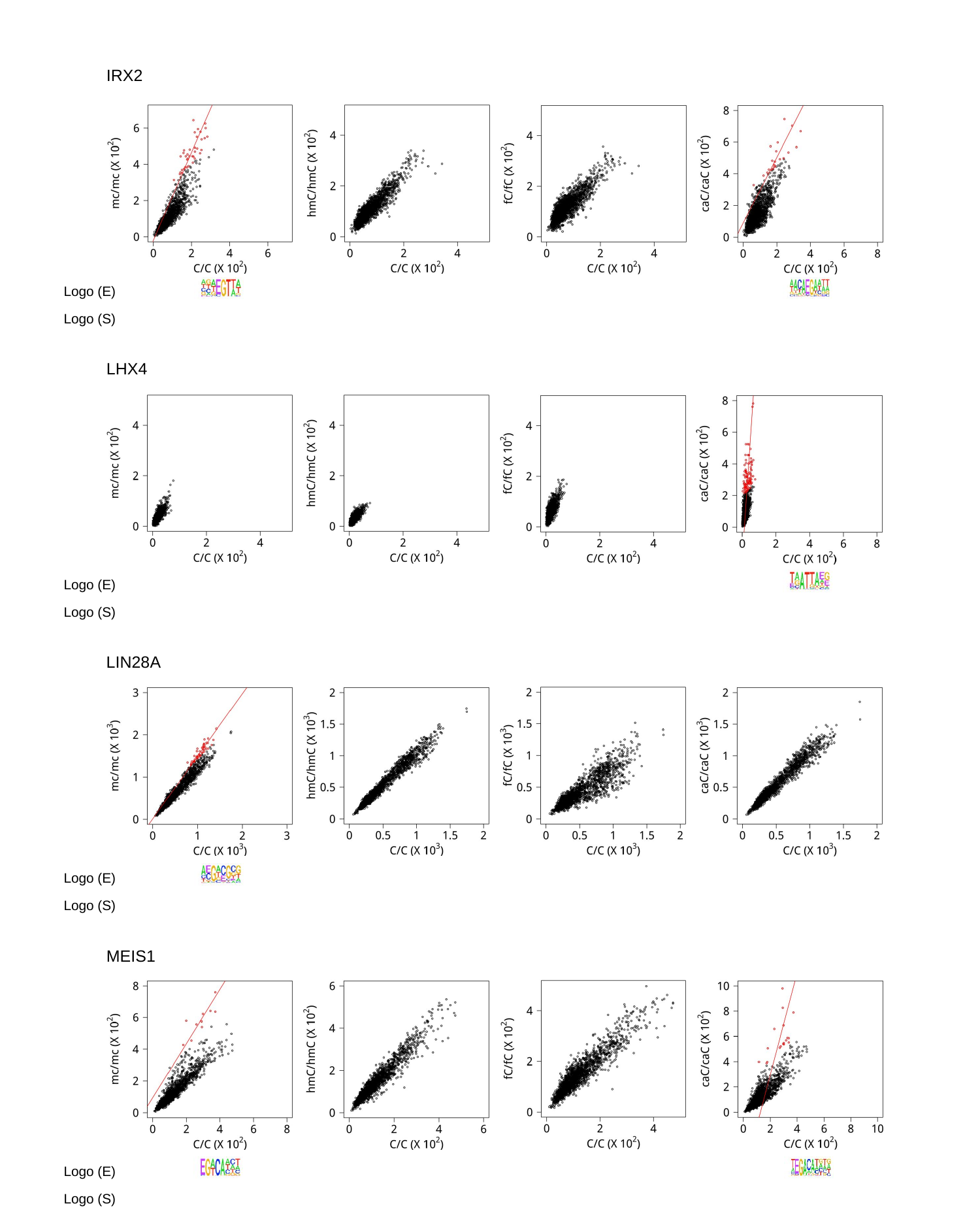

IRX2
Logo (E)
Logo (S)
LHX4
Logo (E)
Logo (S)
LIN28A
Logo (E)
Logo (S)
MEIS1
Logo (E)
Logo (S)

## Slide 6
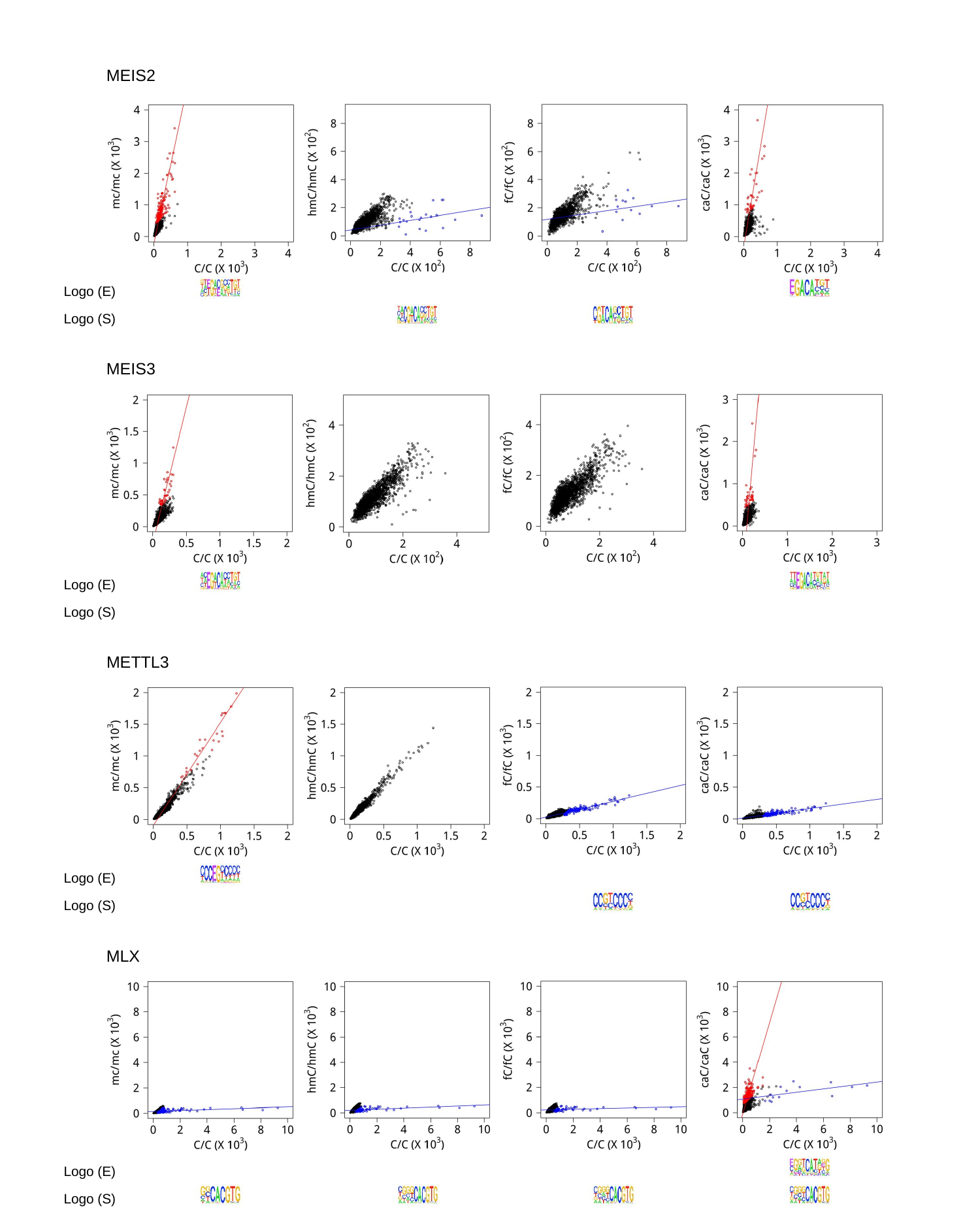

MEIS2
Logo (E)
Logo (S)
MEIS3
Logo (E)
Logo (S)
METTL3
Logo (E)
Logo (S)
MLX
Logo (E)
Logo (S)

## Slide 7
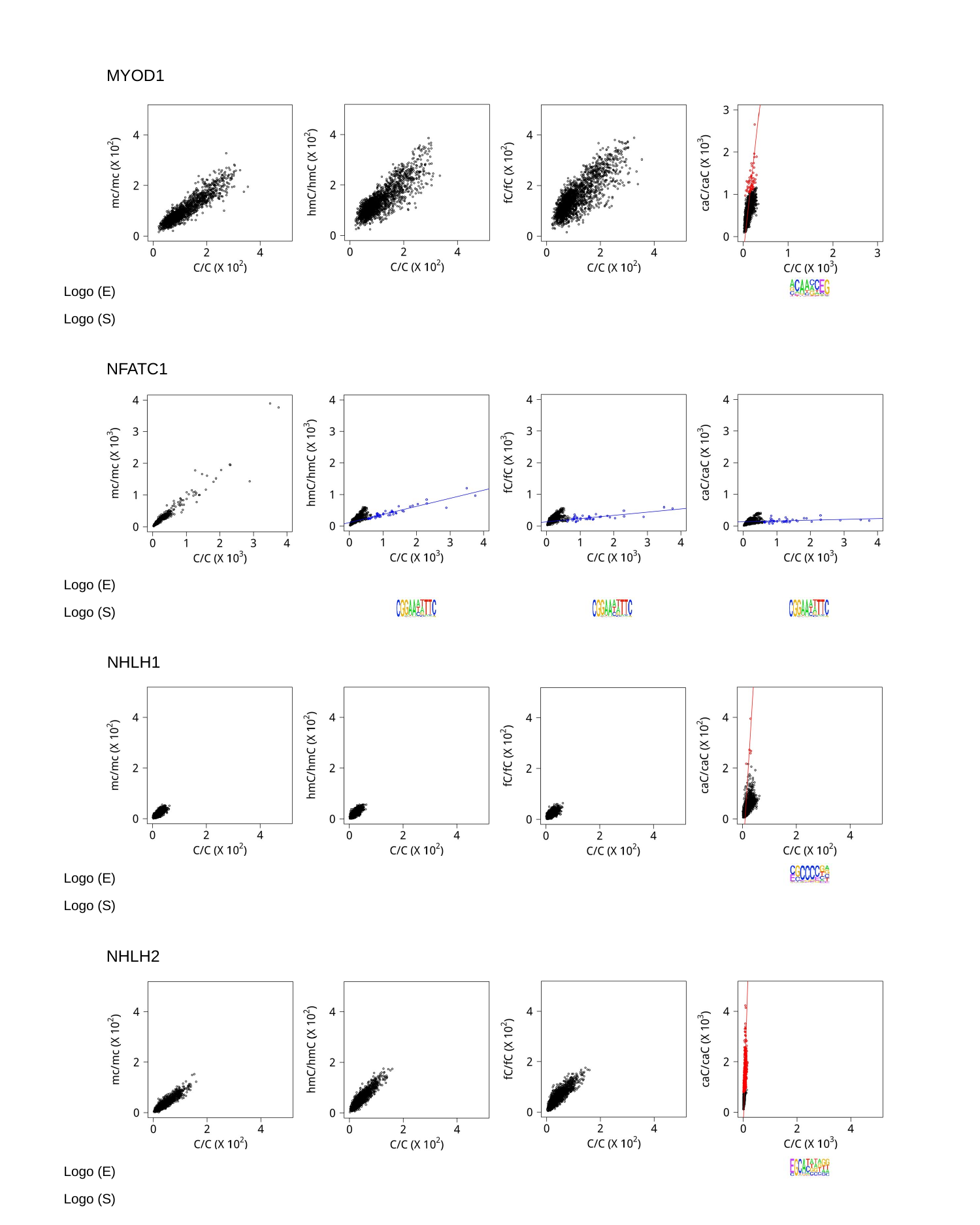

MYOD1
Logo (E)
Logo (S)
NFATC1
Logo (E)
Logo (S)
NHLH1
Logo (E)
Logo (S)
NHLH2
Logo (E)
Logo (S)

## Slide 8
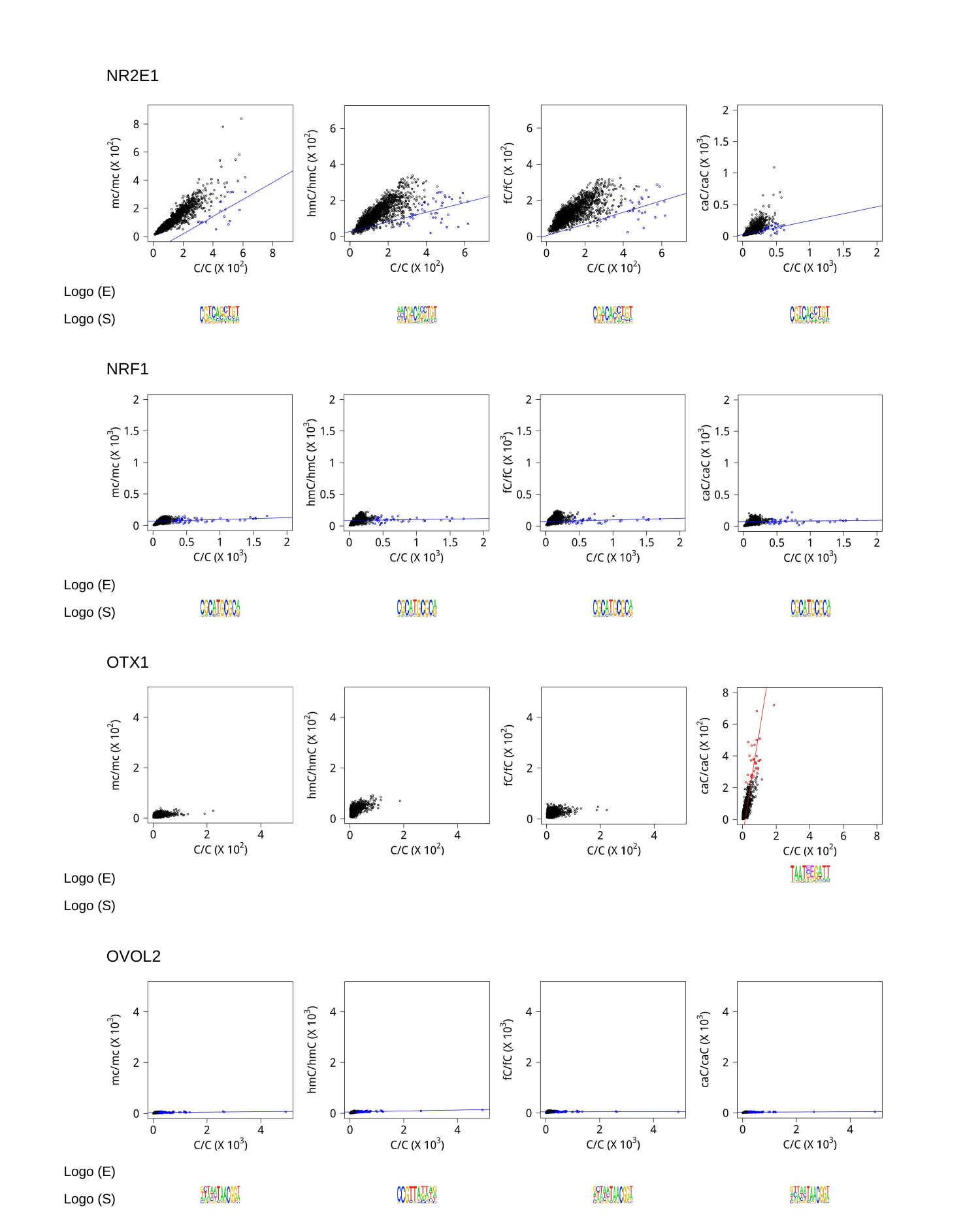

NR2E1
Logo (E)
Logo (S)
NRF1
Logo (E)
Logo (S)
OTX1
Logo (E)
Logo (S)
OVOL2
Logo (E)
Logo (S)

## Slide 9
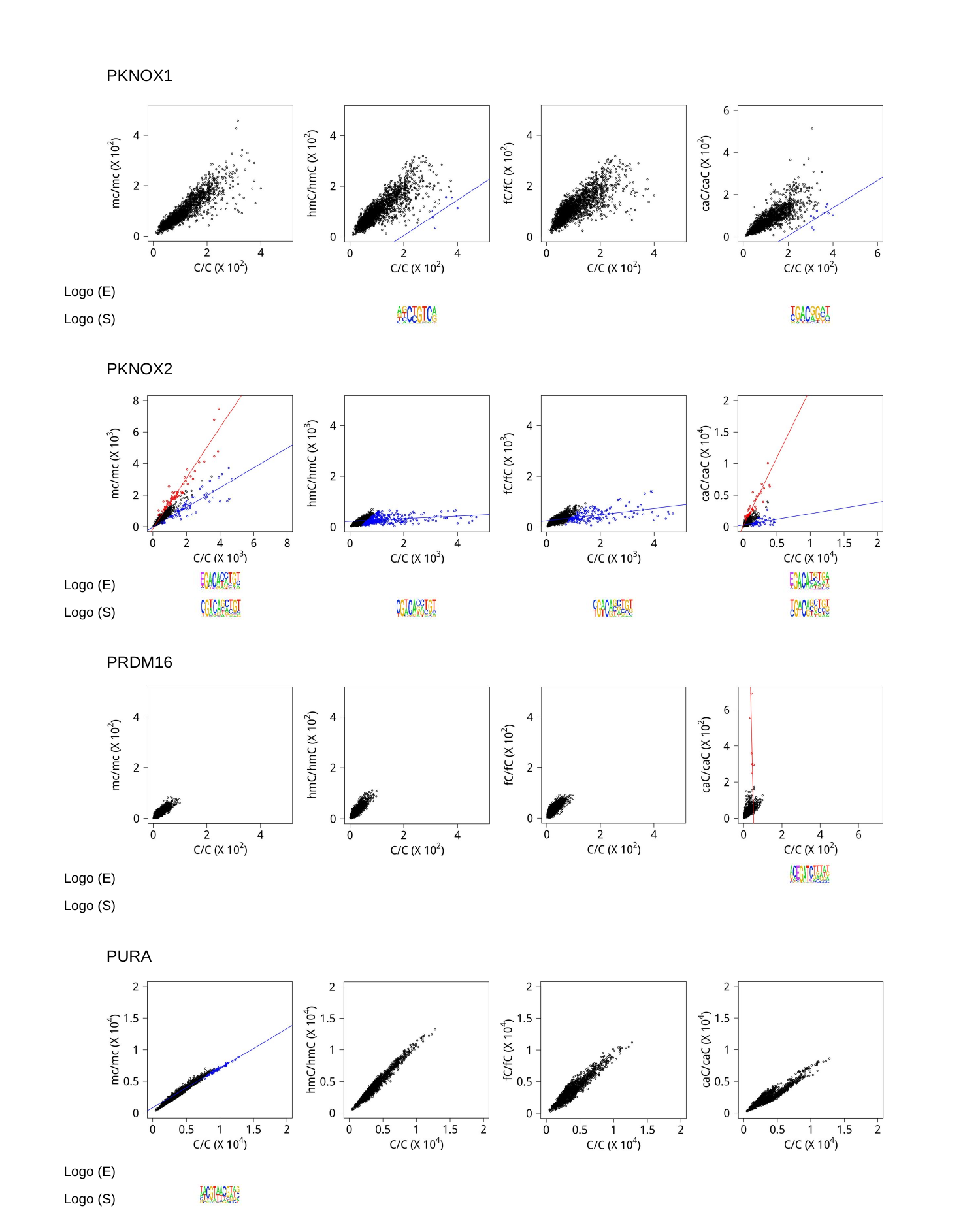

PKNOX1
Logo (E)
Logo (S)
PKNOX2
Logo (E)
Logo (S)
PRDM16
Logo (E)
Logo (S)
PURA
Logo (E)
Logo (S)

## Slide 10
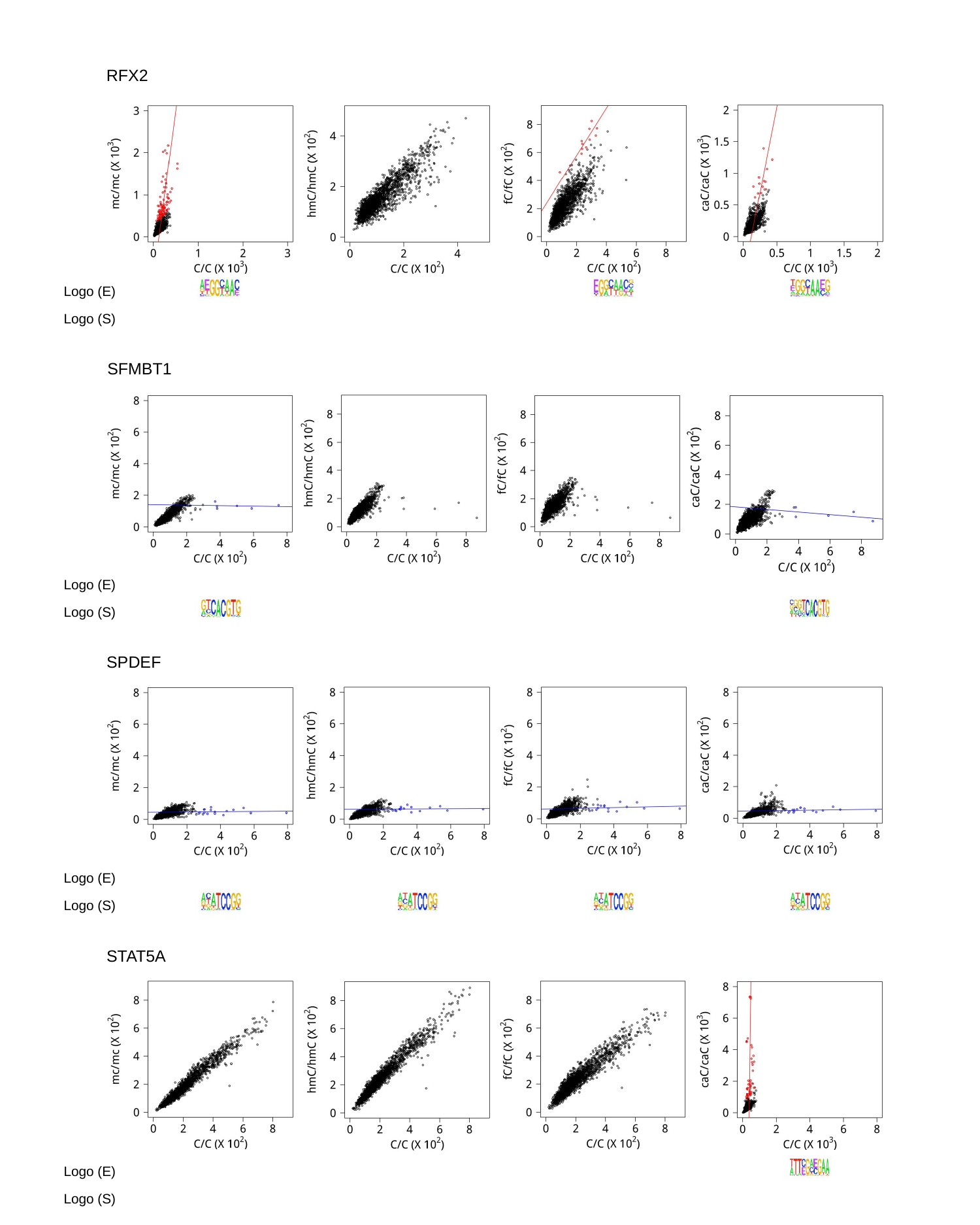

RFX2
Logo (E)
Logo (S)
SFMBT1
Logo (E)
Logo (S)
SPDEF
Logo (E)
Logo (S)
STAT5A
Logo (E)
Logo (S)

## Slide 11
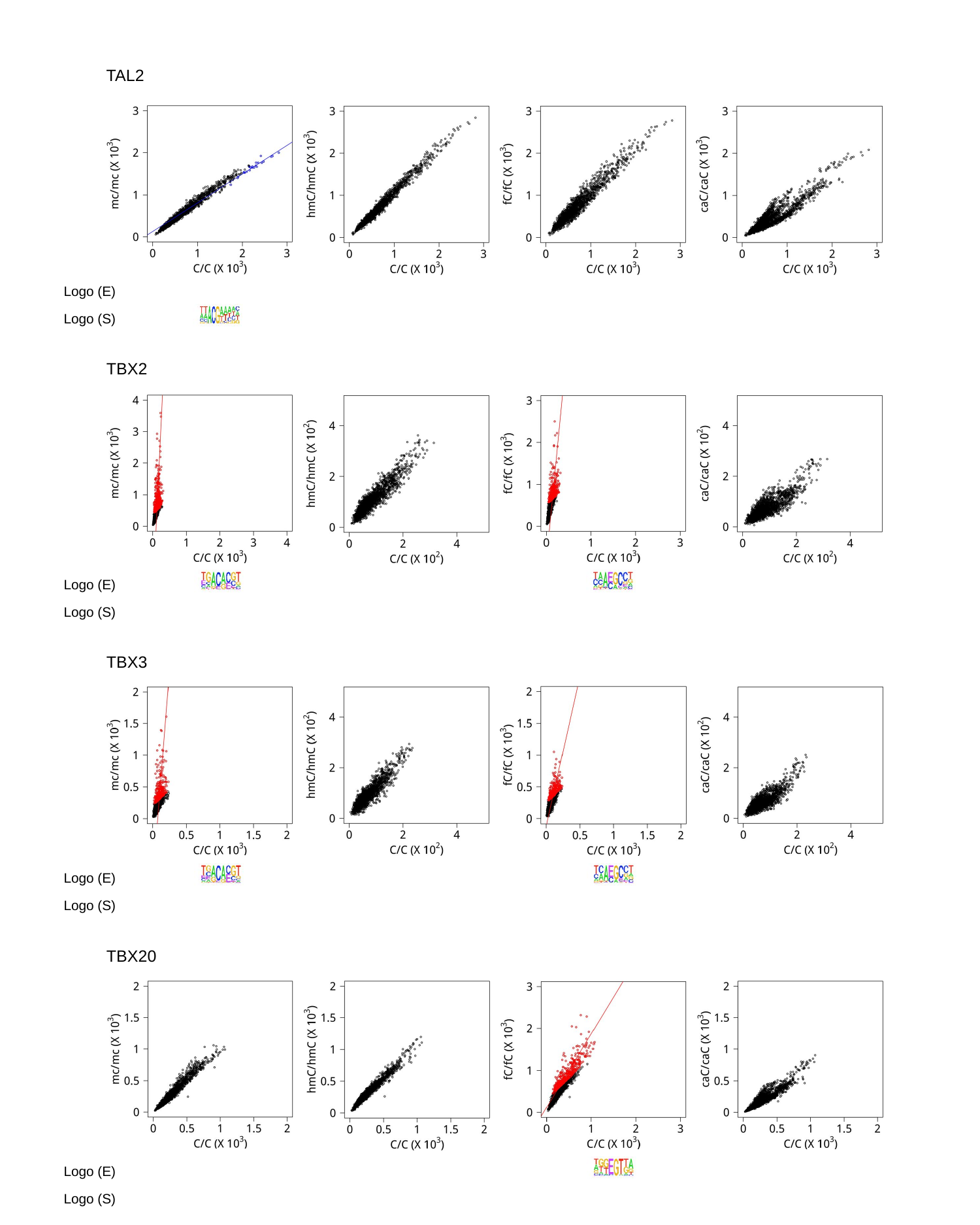

TAL2
Logo (E)
Logo (S)
TBX2
Logo (E)
Logo (S)
TBX3
Logo (E)
Logo (S)
TBX20
Logo (E)
Logo (S)

## Slide 12
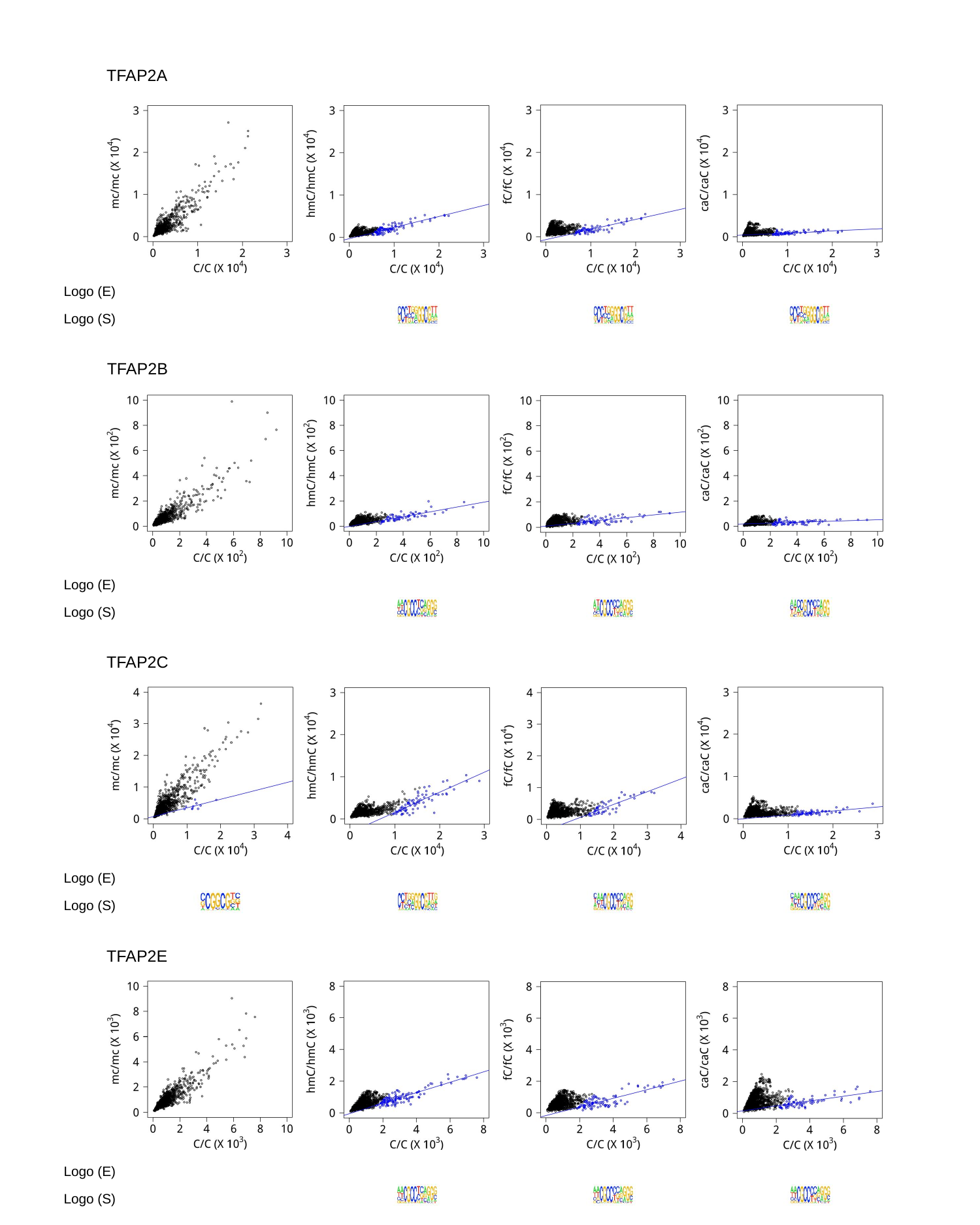

TFAP2A
Logo (E)
Logo (S)
TFAP2B
Logo (E)
Logo (S)
TFAP2C
Logo (E)
Logo (S)
TFAP2E
Logo (E)
Logo (S)

## Slide 13
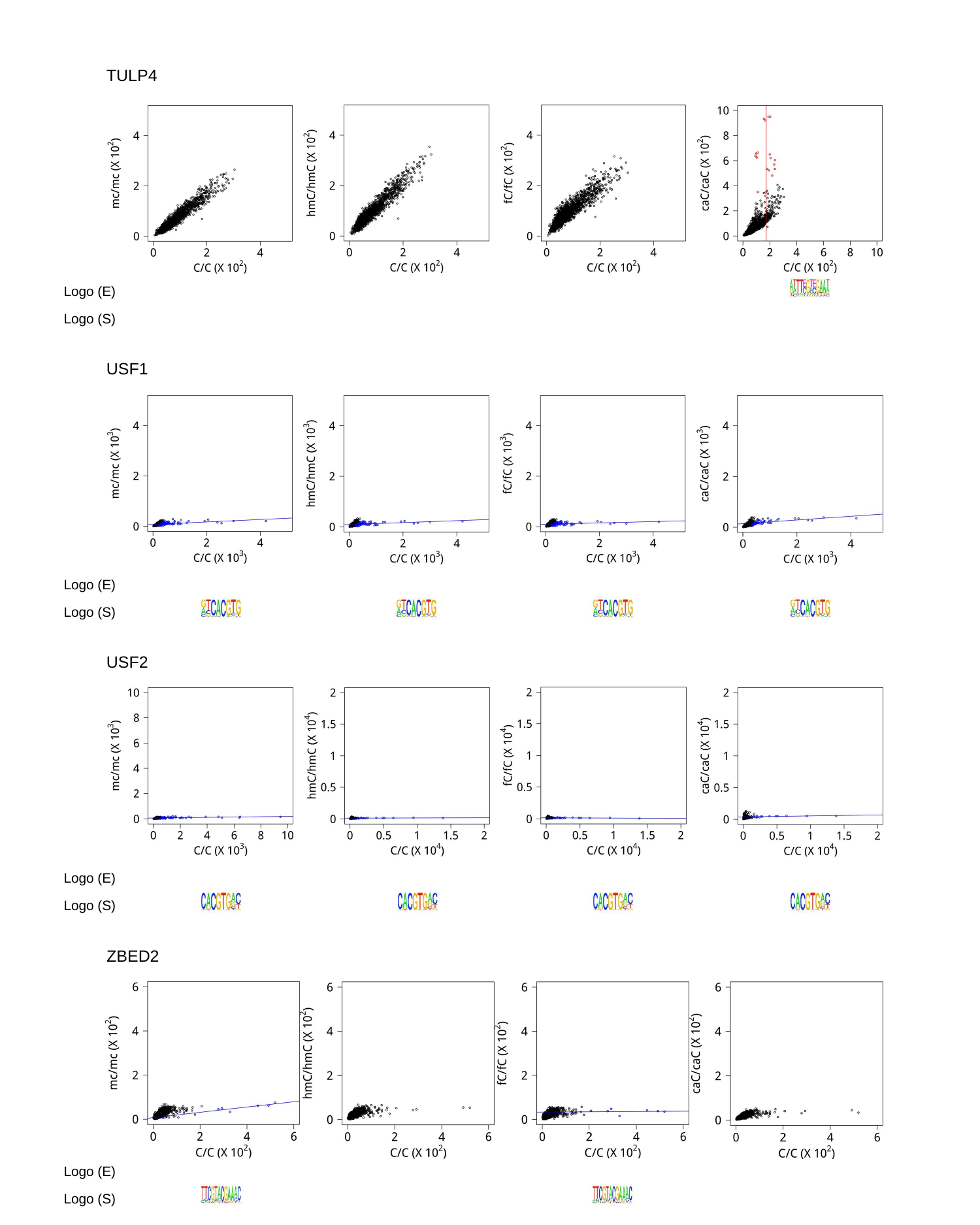

TULP4
Logo (E)
Logo (S)
USF1
Logo (E)
Logo (S)
USF2
Logo (E)
Logo (S)
ZBED2
Logo (E)
Logo (S)

## Slide 14
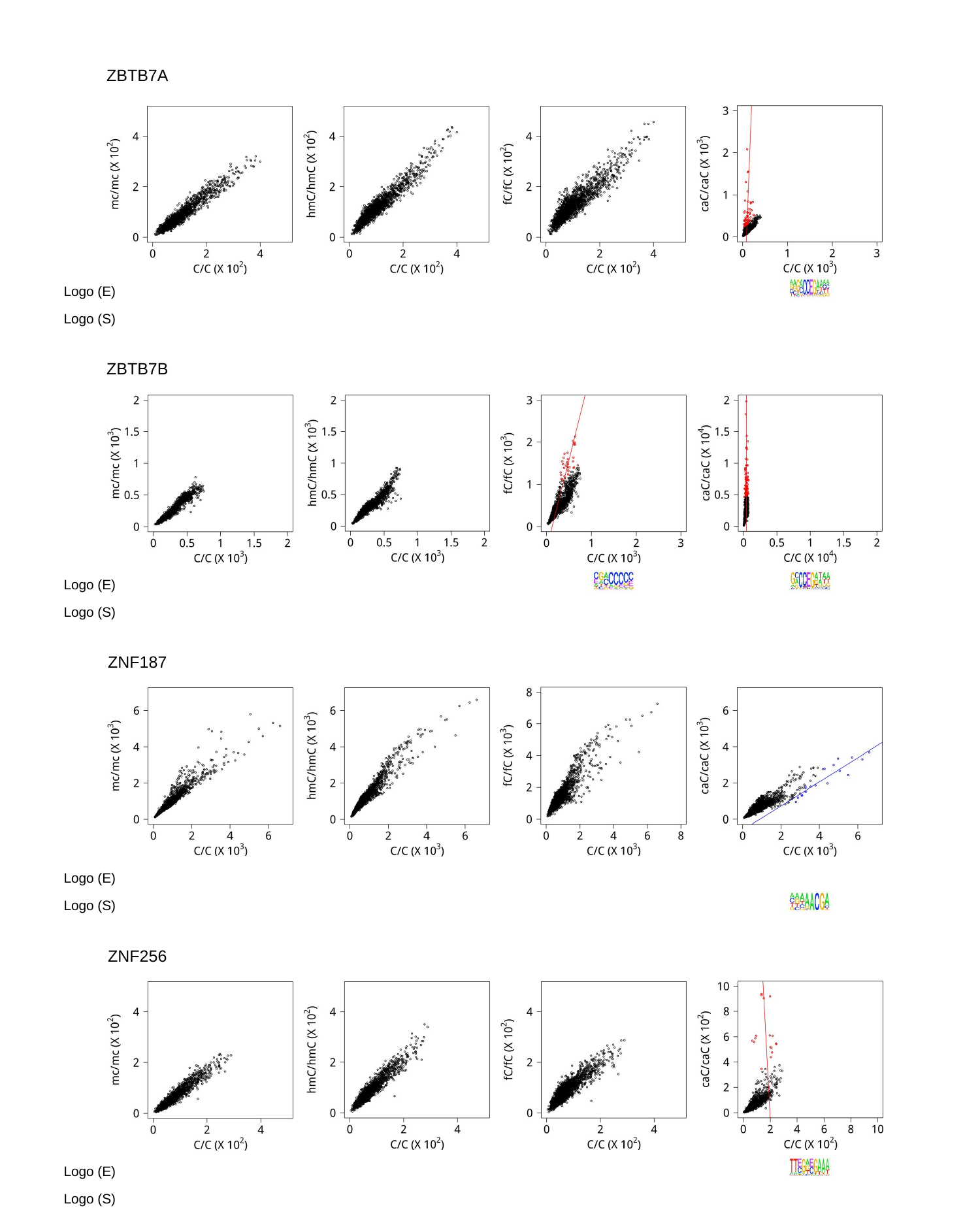

ZBTB7A
Logo (E)
Logo (S)
ZBTB7B
Logo (E)
Logo (S)
ZNF187
Logo (E)
Logo (S)
ZNF256
Logo (E)
Logo (S)

## Slide 15
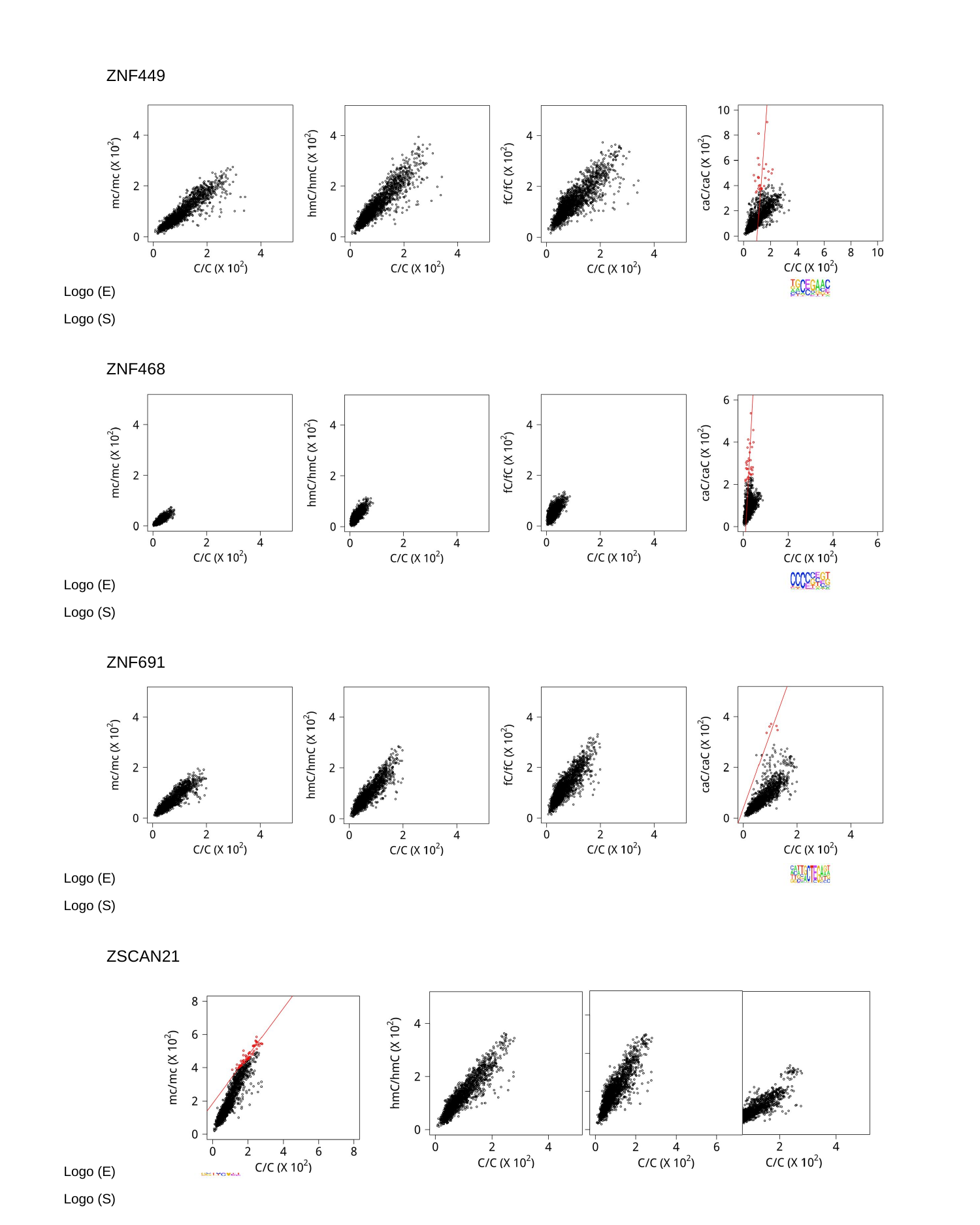

ZNF449
Logo (E)
Logo (S)
ZNF468
Logo (E)
Logo (S)
ZNF691
Logo (E)
Logo (S)
ZSCAN21
Logo (E)
Logo (S)

## Slide 16
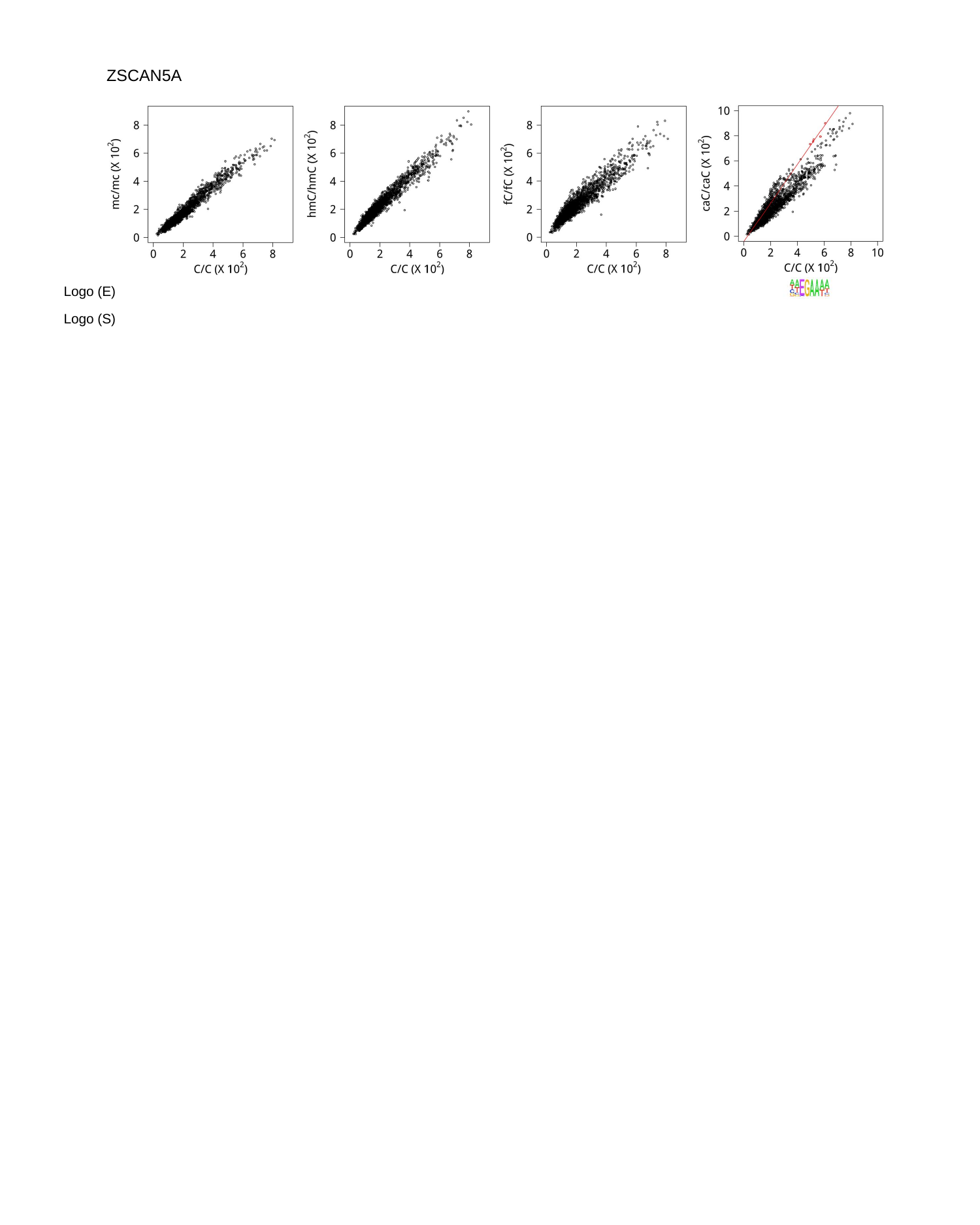

ZSCAN5A
Logo (E)
Logo (S)
