# Supplementary material for: An all-to-all approach to the identification of sequence-specific readers for epigenetic DNA modifications on cytosine
Source: Nat Commun. 2021 Feb 4;12:795. doi: 10.1038/s41467-021-20950-w (PMC7862700; doi:10.1038/s41467-021-20950-w)
Supplement: Supplementary file 11 — Supplementary Data 8 [file 41467_2021_20950_MOESM11_ESM.pptx]

## Slide 1
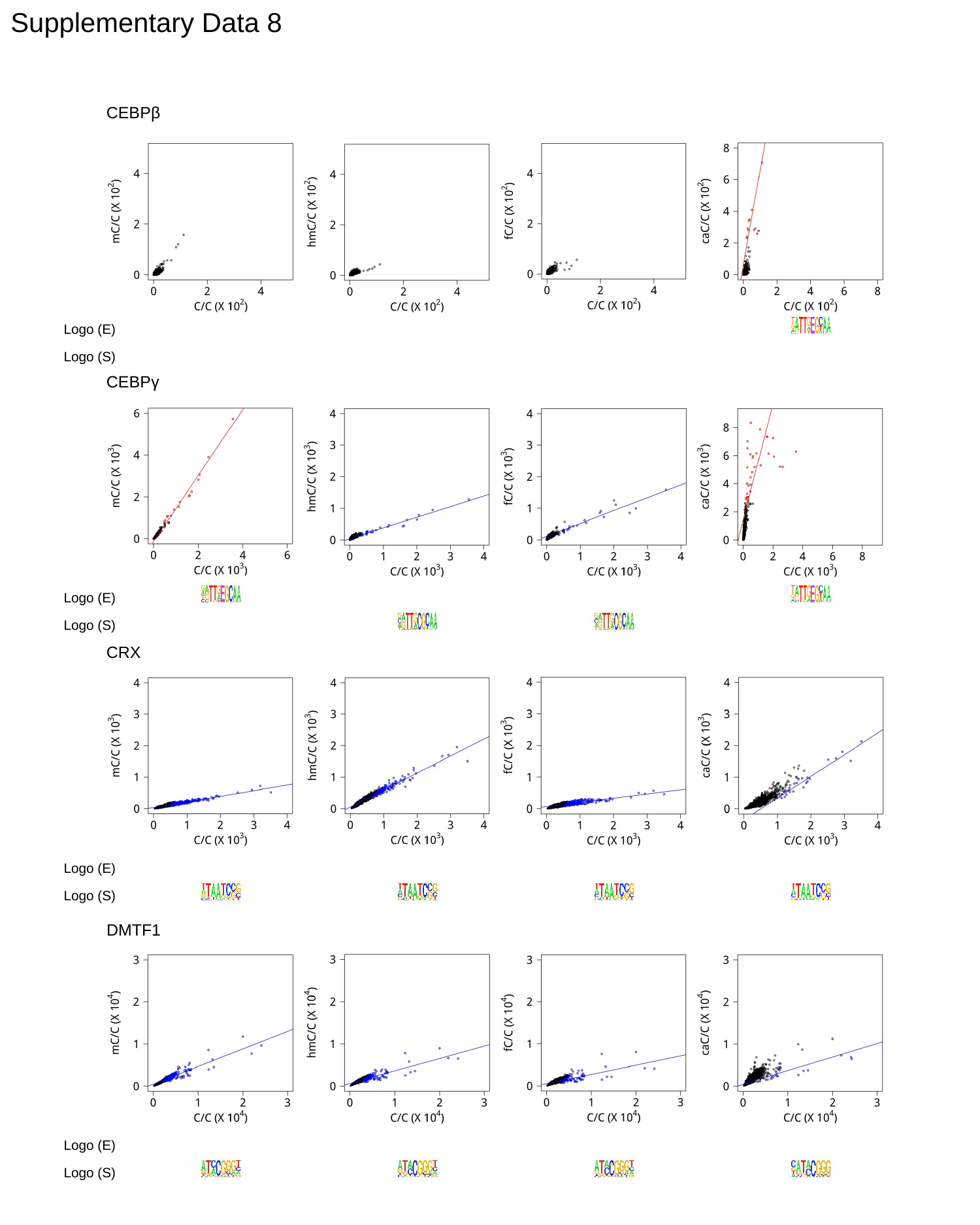

Supplementary Data 8
CEBPβ
Logo (E)
Logo (S)
CEBPγ
Logo (E)
Logo (S)
CRX
Logo (E)
Logo (S)
DMTF1
Logo (E)
Logo (S)

## Slide 2
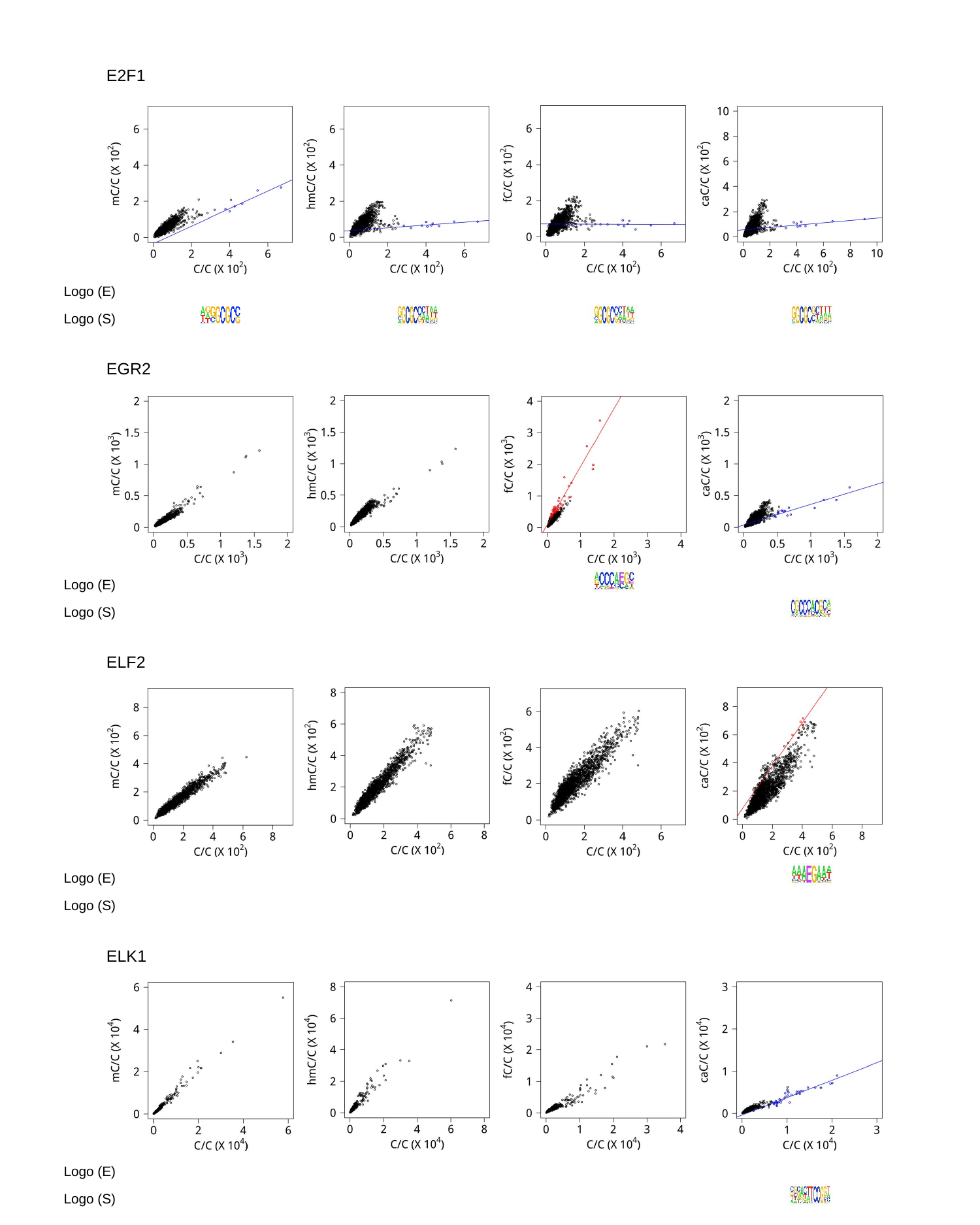

E2F1
Logo (E)
Logo (S)
EGR2
Logo (E)
Logo (S)
ELF2
Logo (E)
Logo (S)
ELK1
Logo (E)
Logo (S)

## Slide 3
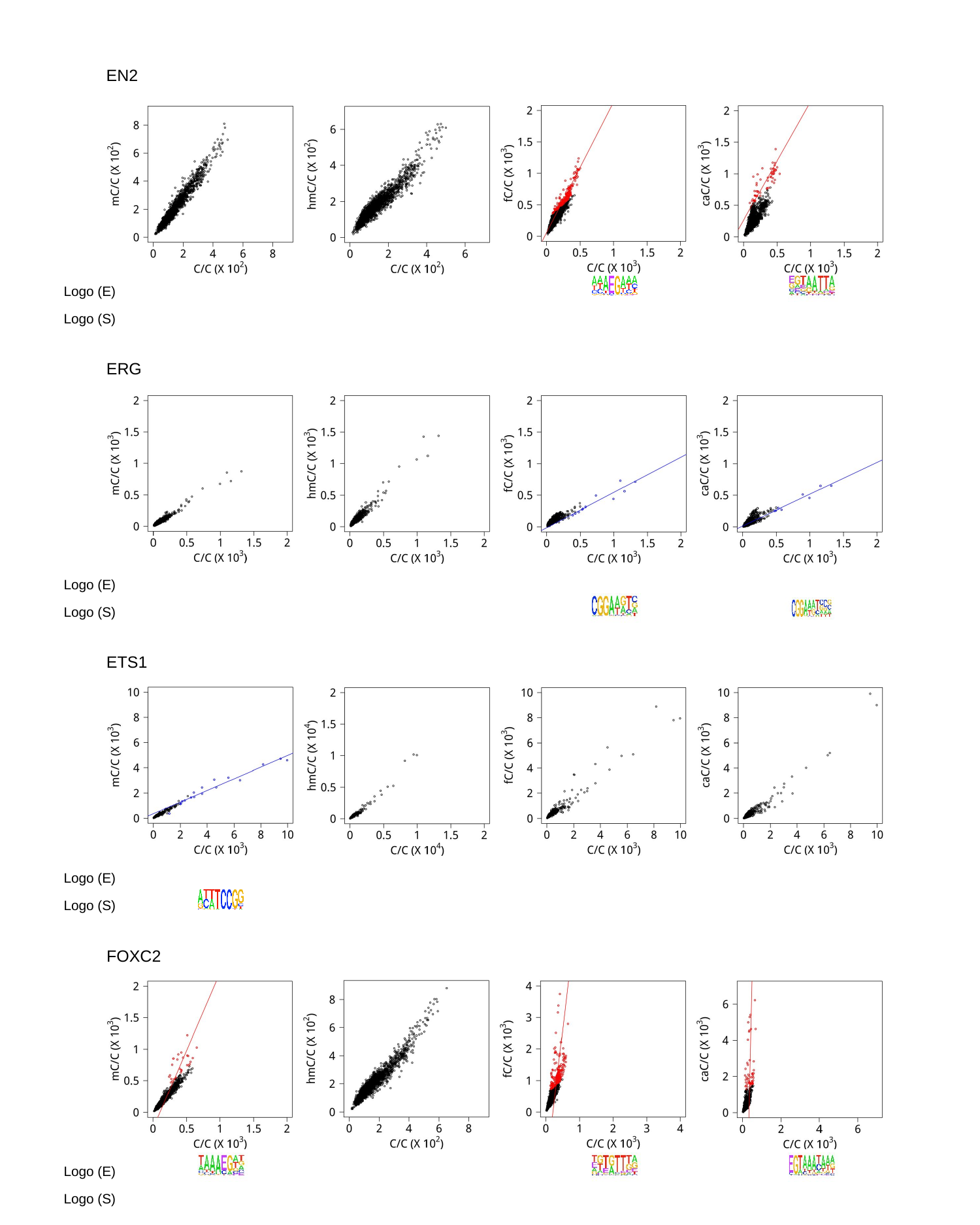

EN2
Logo (E)
Logo (S)
ERG
Logo (E)
Logo (S)
ETS1
Logo (E)
Logo (S)
FOXC2
Logo (E)
Logo (S)

## Slide 4
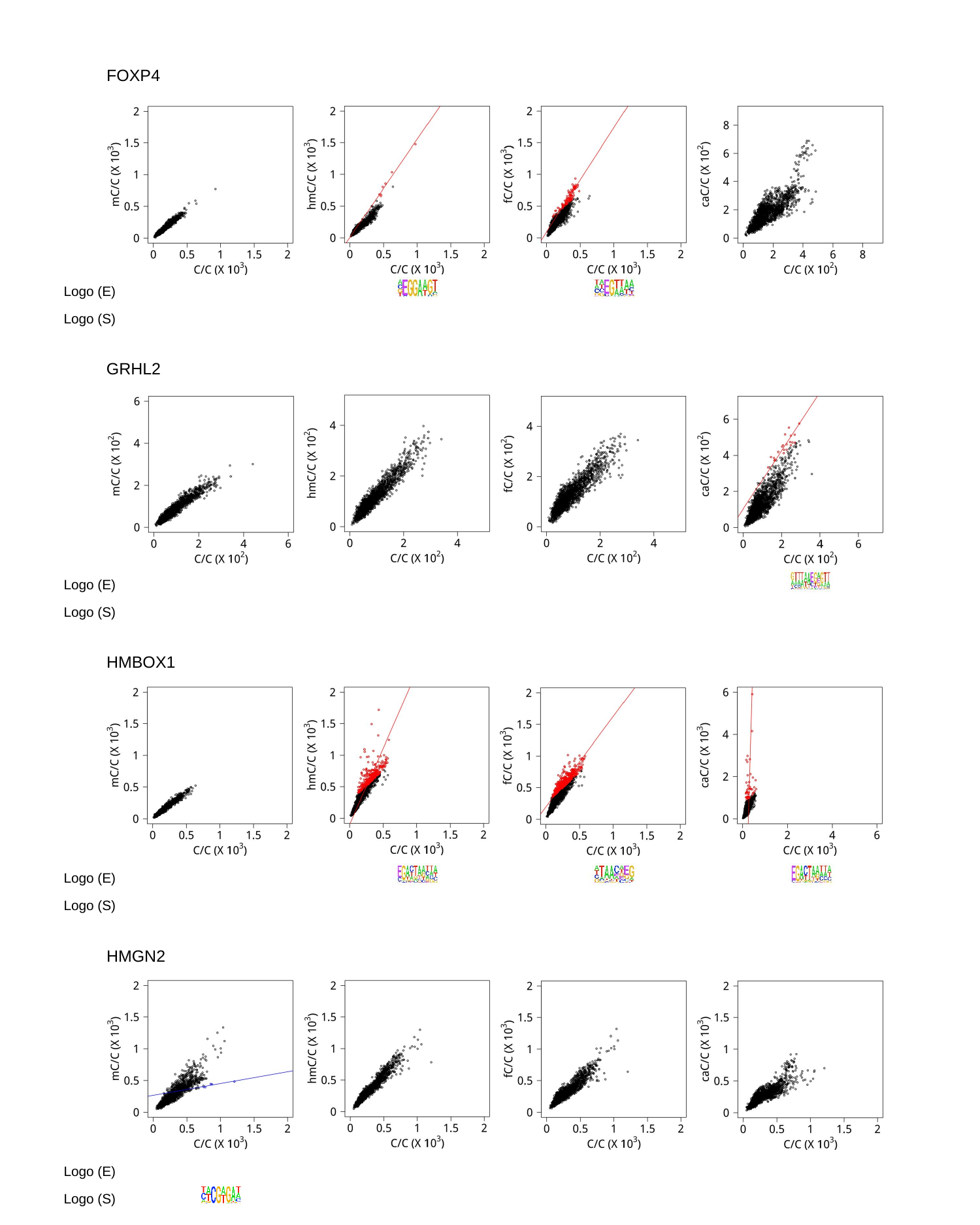

FOXP4
Logo (E)
Logo (S)
GRHL2
Logo (E)
Logo (S)
HMBOX1
Logo (E)
Logo (S)
HMGN2
Logo (E)
Logo (S)

## Slide 5
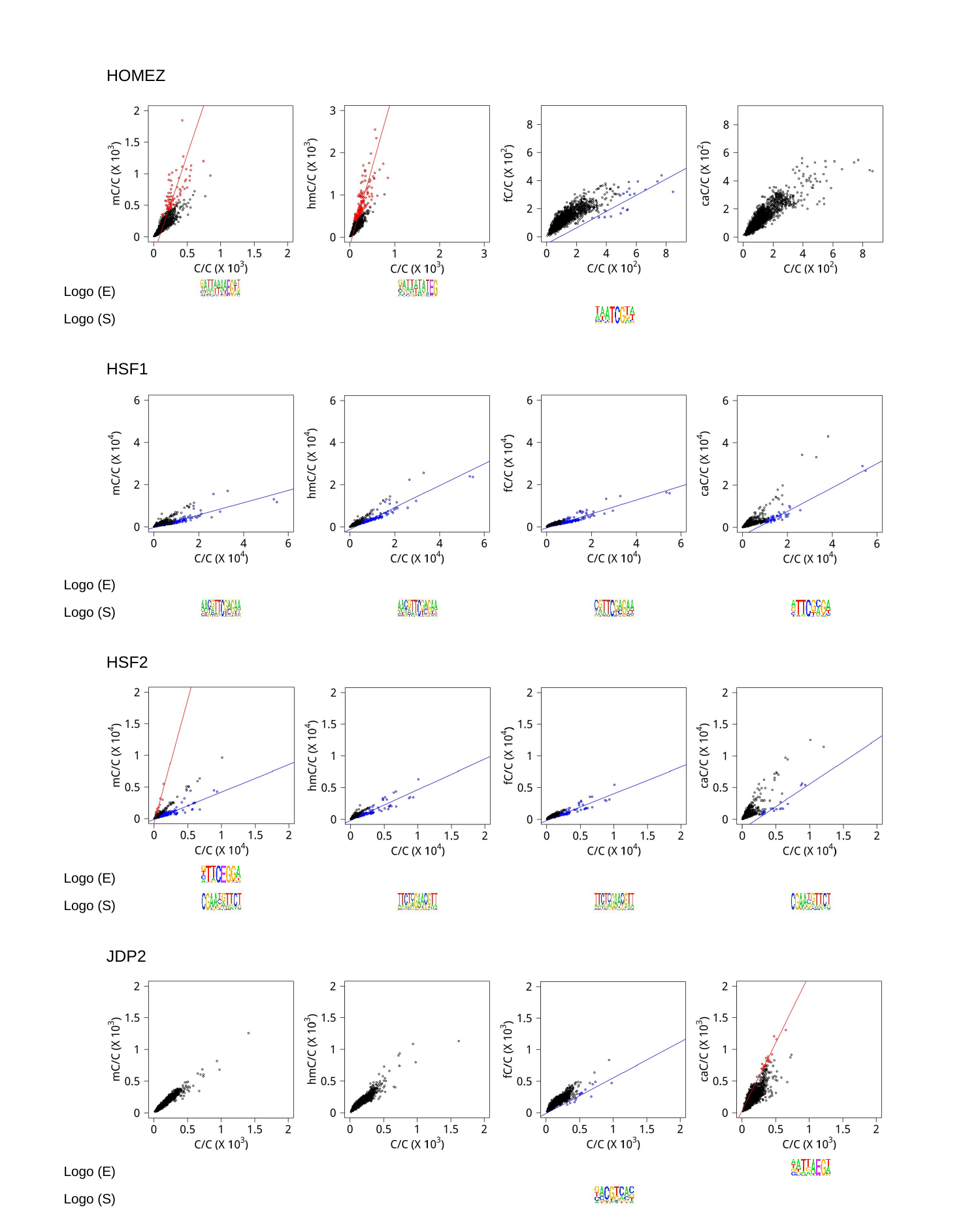

HOMEZ
Logo (E)
Logo (S)
HSF1
Logo (E)
Logo (S)
HSF2
Logo (E)
Logo (S)
JDP2
Logo (E)
Logo (S)

## Slide 6
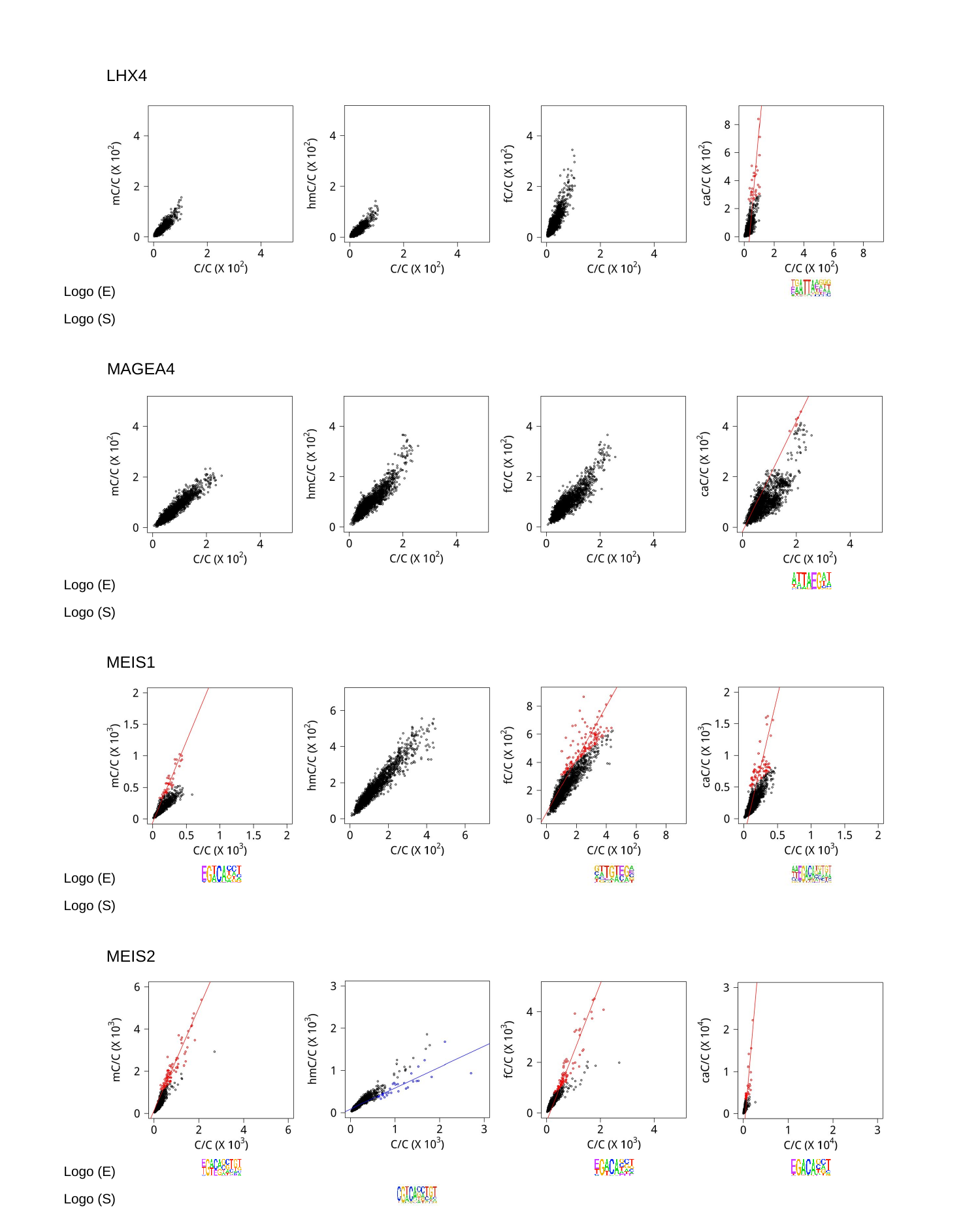

LHX4
Logo (E)
Logo (S)
MAGEA4
Logo (E)
Logo (S)
MEIS1
Logo (E)
Logo (S)
MEIS2
Logo (E)
Logo (S)

## Slide 7
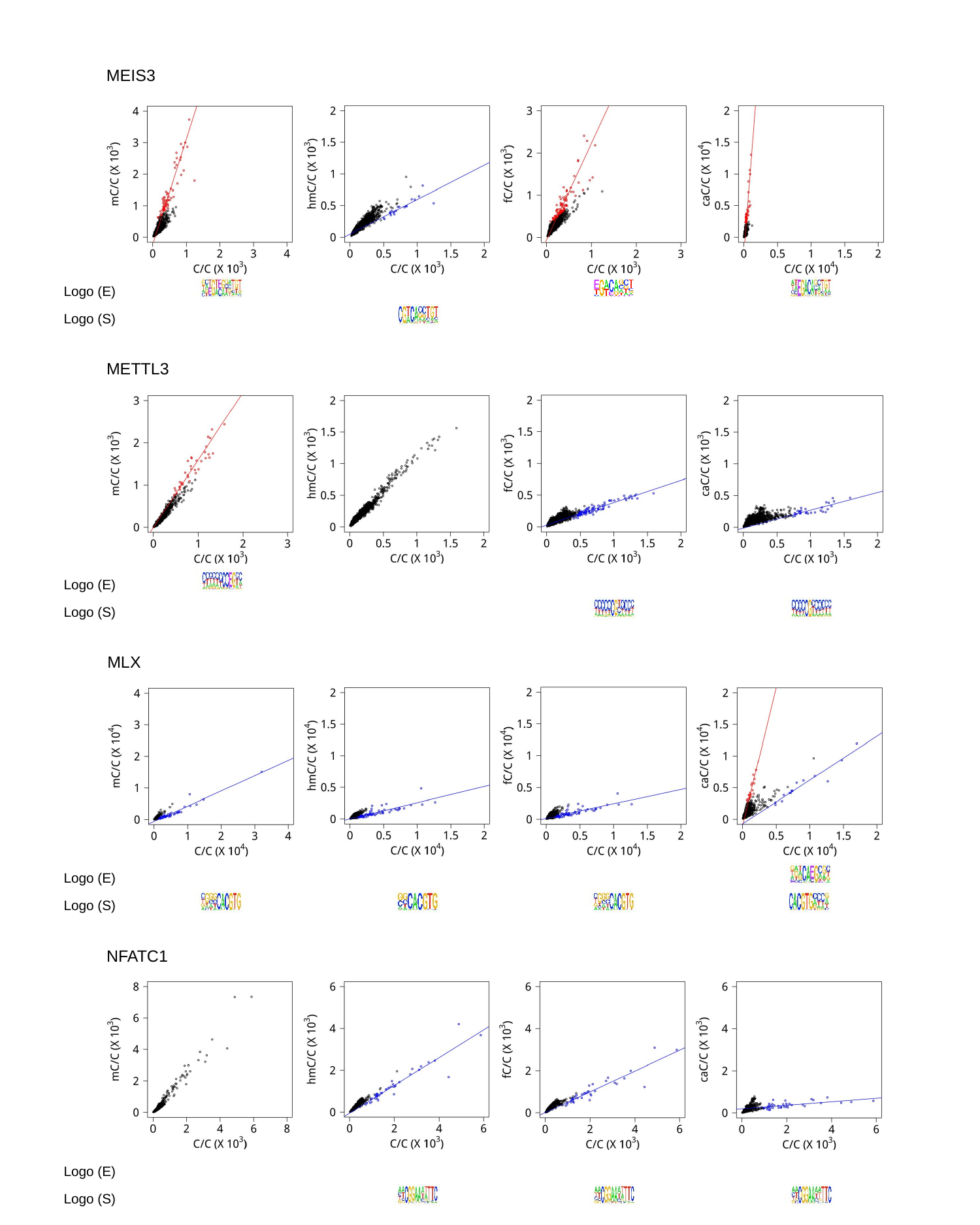

MEIS3
Logo (E)
Logo (S)
METTL3
Logo (E)
Logo (S)
MLX
Logo (E)
Logo (S)
NFATC1
Logo (E)
Logo (S)

## Slide 8
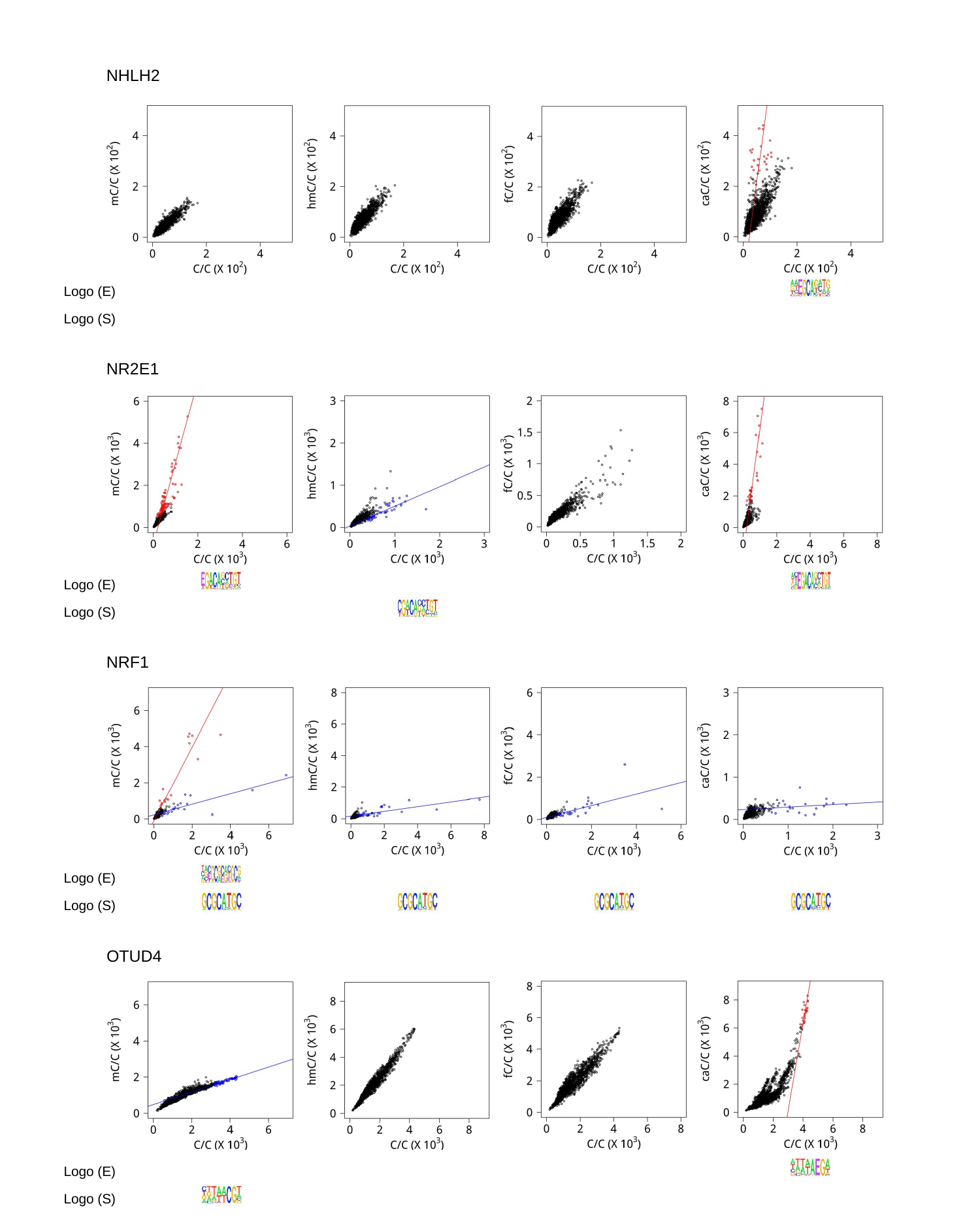

NHLH2
Logo (E)
Logo (S)
NR2E1
Logo (E)
Logo (S)
NRF1
Logo (E)
Logo (S)
OTUD4
Logo (E)
Logo (S)

## Slide 9
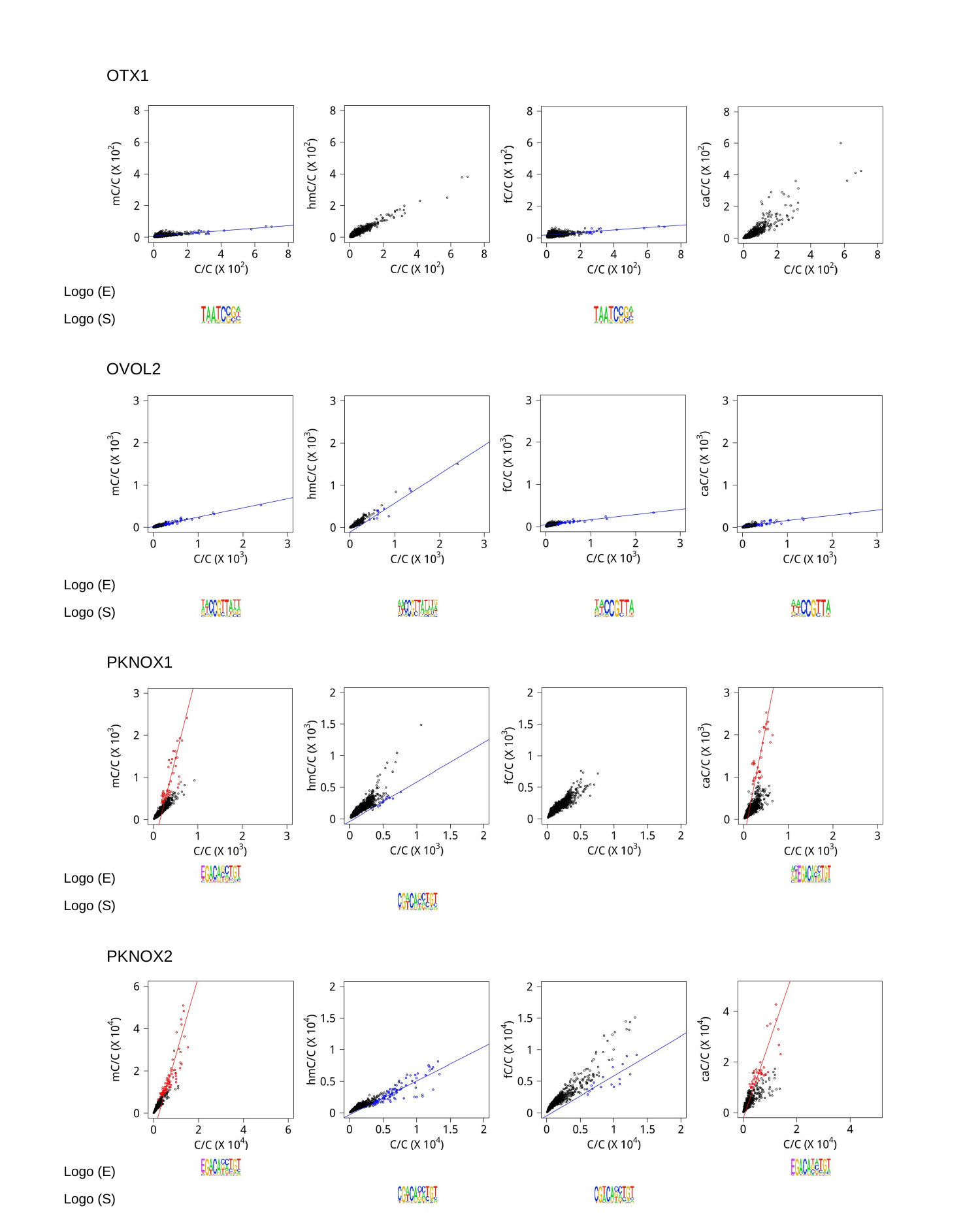

OTX1
Logo (E)
Logo (S)
OVOL2
Logo (E)
Logo (S)
PKNOX1
Logo (E)
Logo (S)
PKNOX2
Logo (E)
Logo (S)

## Slide 10
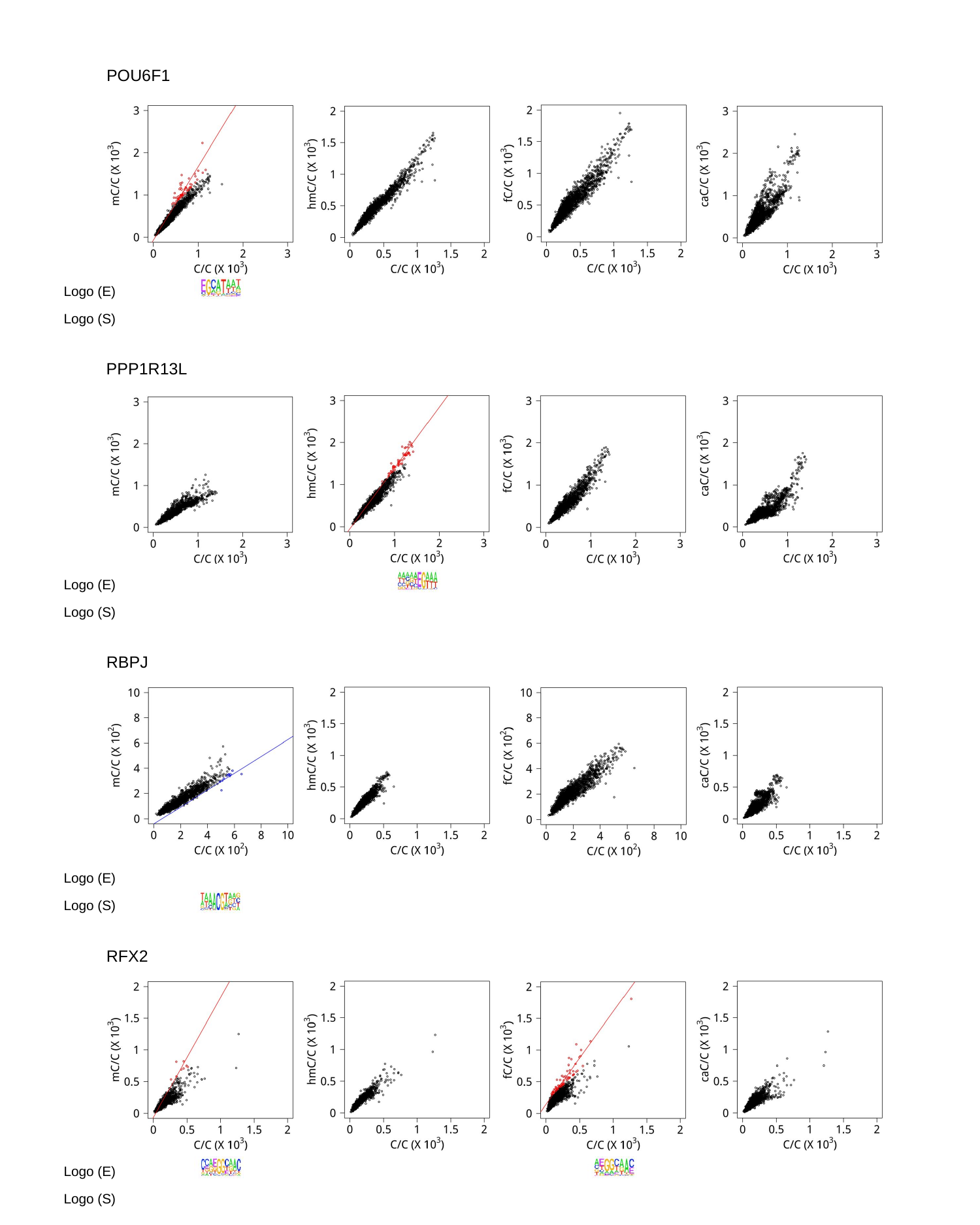

POU6F1
Logo (E)
Logo (S)
PPP1R13L
Logo (E)
Logo (S)
RBPJ
Logo (E)
Logo (S)
RFX2
Logo (E)
Logo (S)

## Slide 11
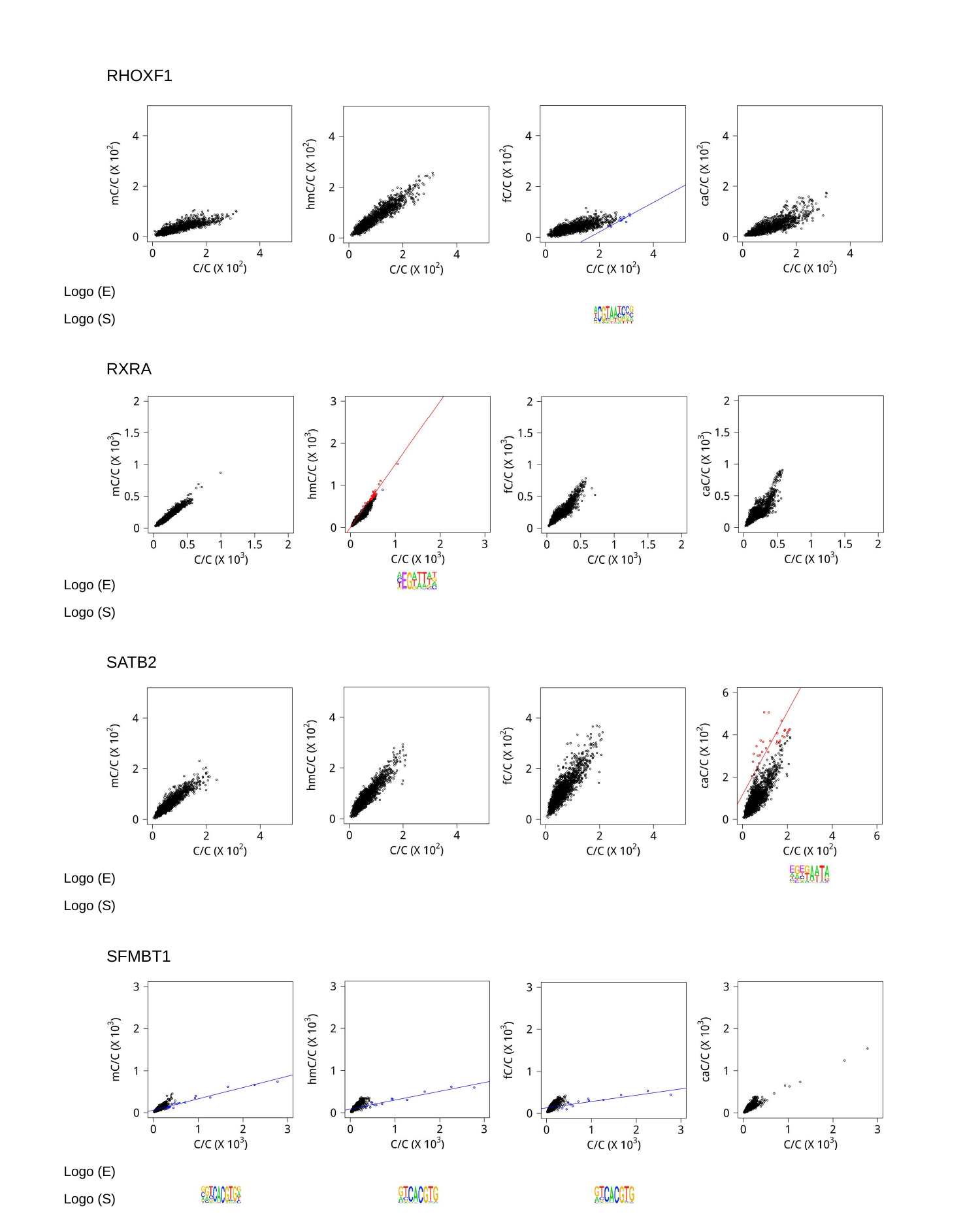

RHOXF1
Logo (E)
Logo (S)
RXRA
Logo (E)
Logo (S)
SATB2
Logo (E)
Logo (S)
SFMBT1
Logo (E)
Logo (S)

## Slide 12
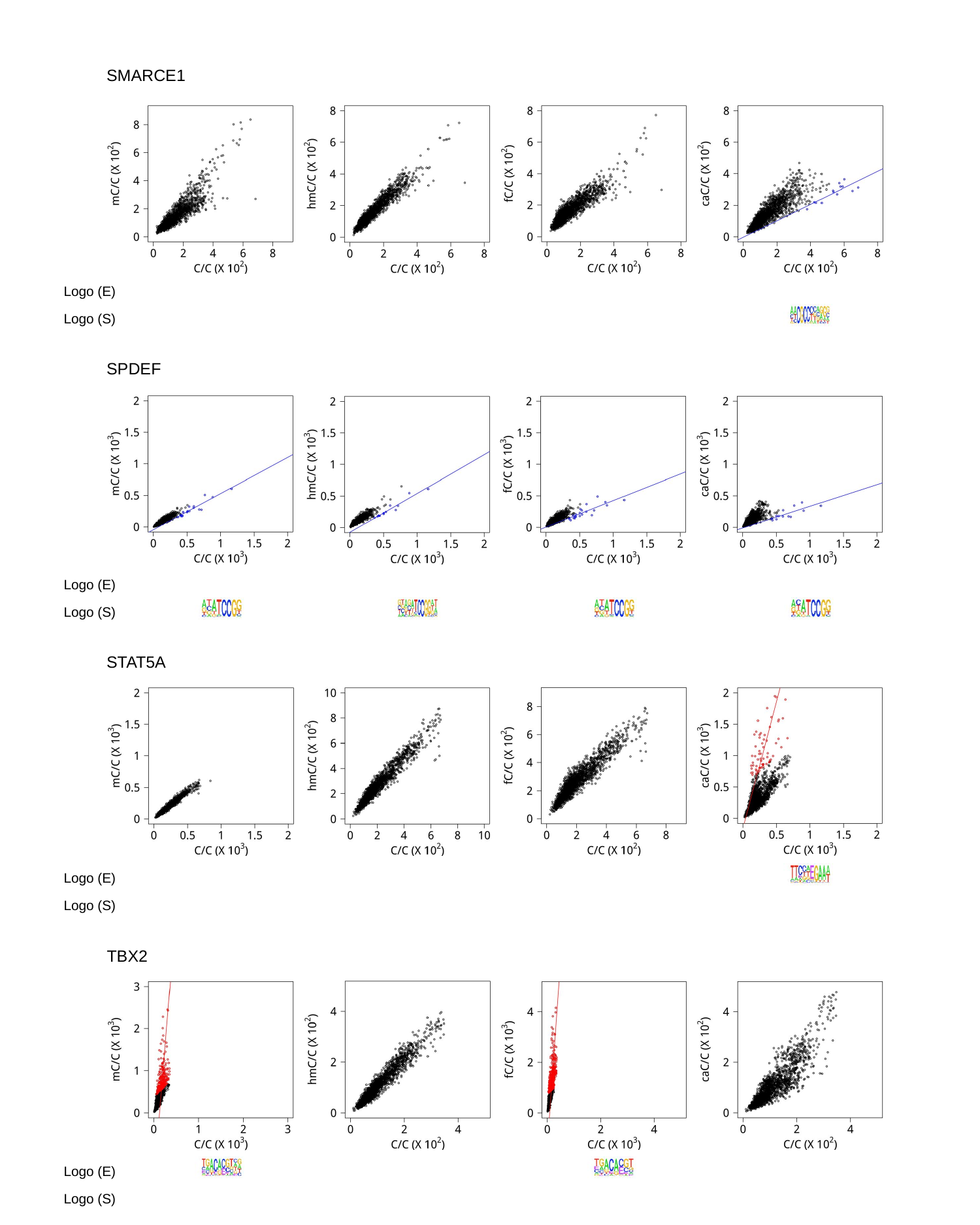

SMARCE1
Logo (E)
Logo (S)
SPDEF
Logo (E)
Logo (S)
STAT5A
Logo (E)
Logo (S)
TBX2
Logo (E)
Logo (S)

## Slide 13
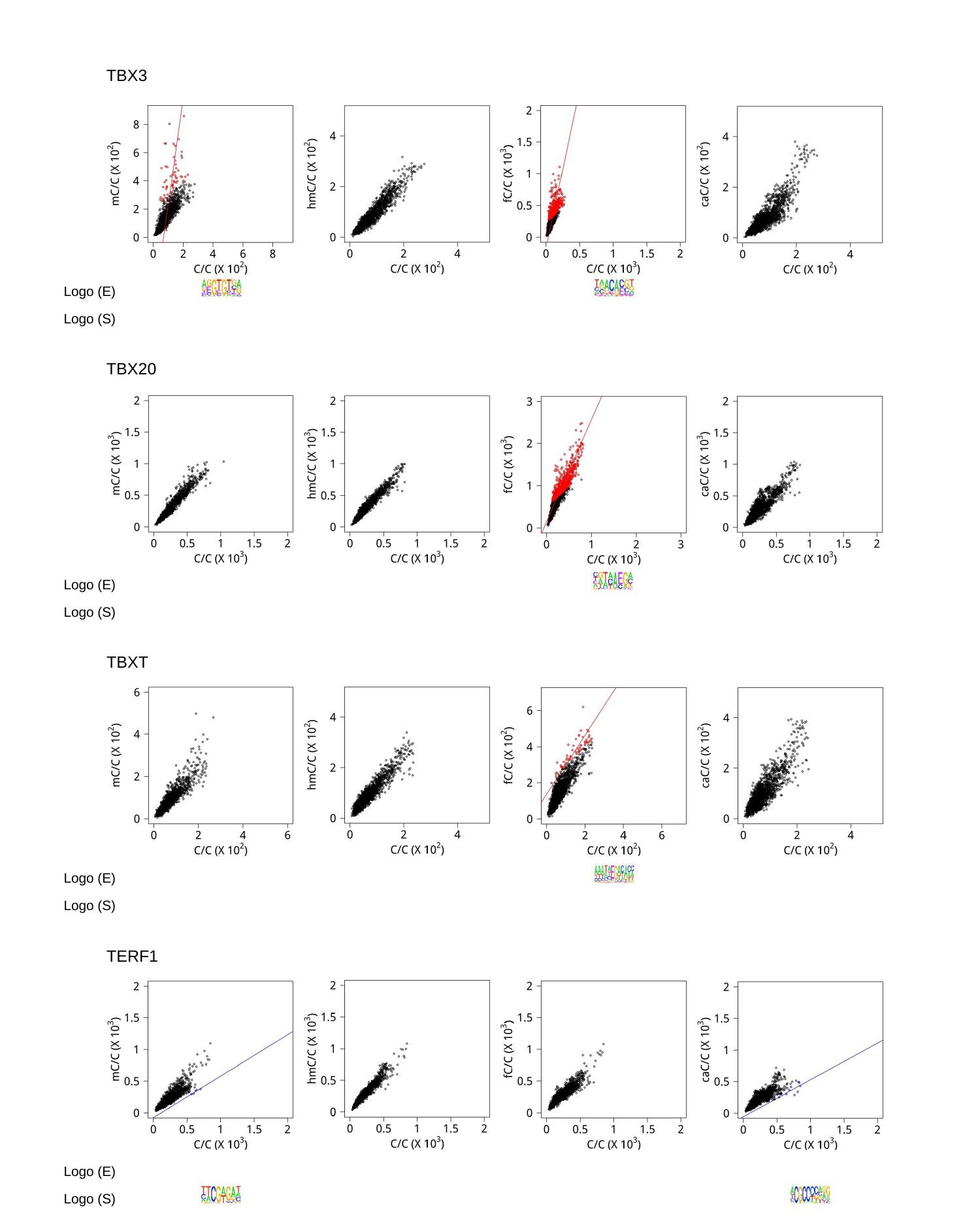

TBX3
Logo (E)
Logo (S)
TBX20
Logo (E)
Logo (S)
TBXT
Logo (E)
Logo (S)
TERF1
Logo (E)
Logo (S)

## Slide 14
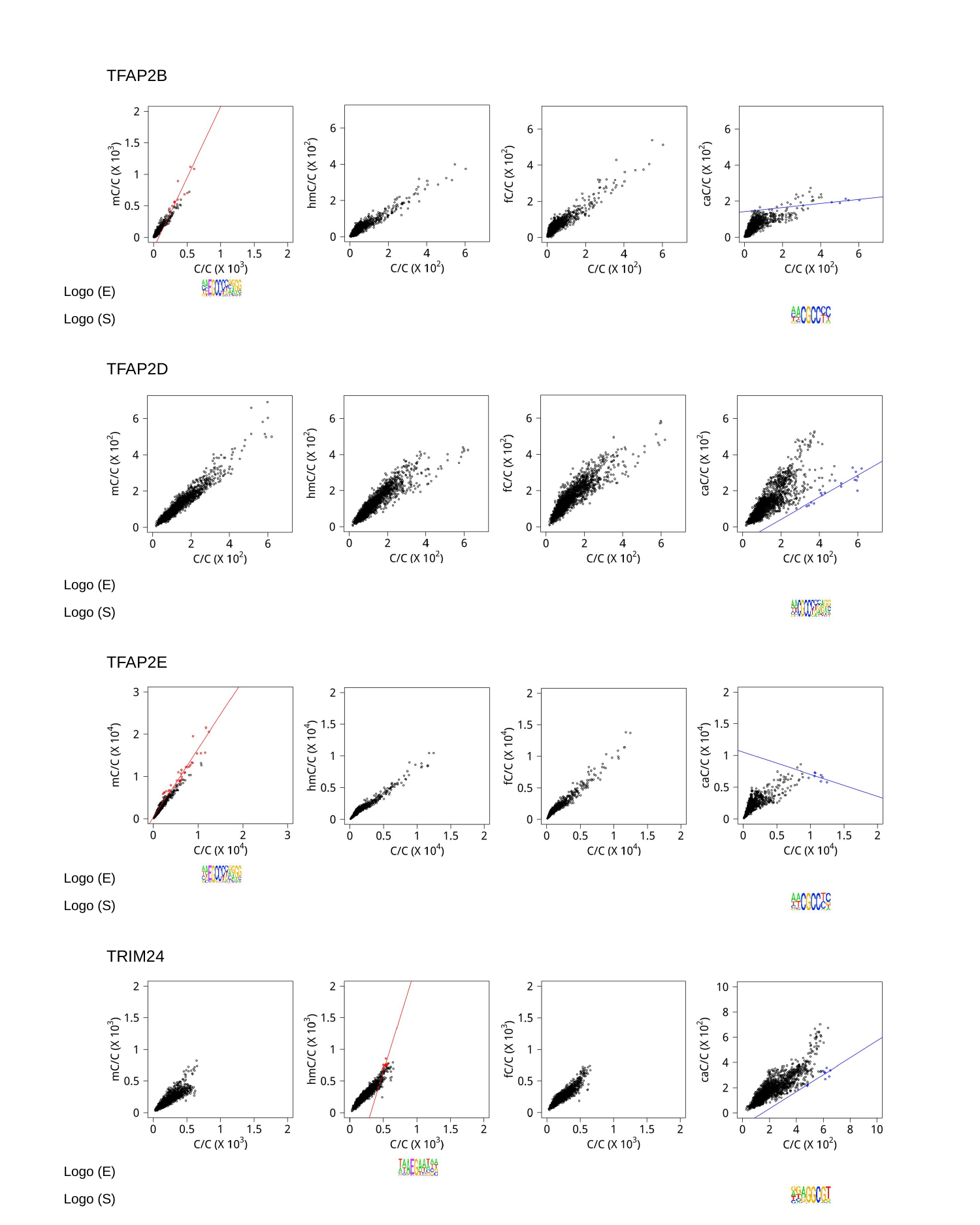

TFAP2B
Logo (E)
Logo (S)
TFAP2D
Logo (E)
Logo (S)
TFAP2E
Logo (E)
Logo (S)
TRIM24
Logo (E)
Logo (S)

## Slide 15
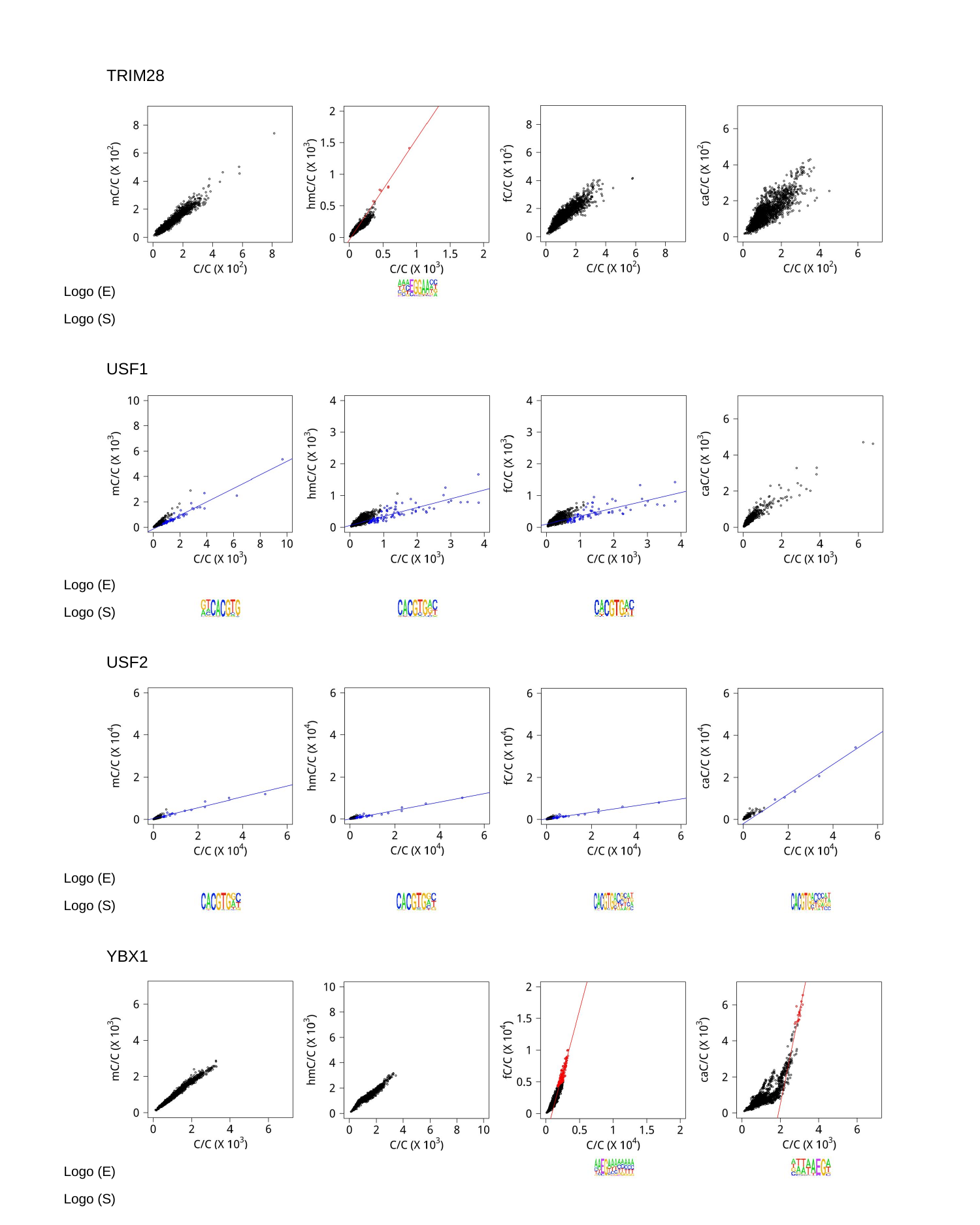

TRIM28
Logo (E)
Logo (S)
USF1
Logo (E)
Logo (S)
USF2
Logo (E)
Logo (S)
YBX1
Logo (E)
Logo (S)

## Slide 16
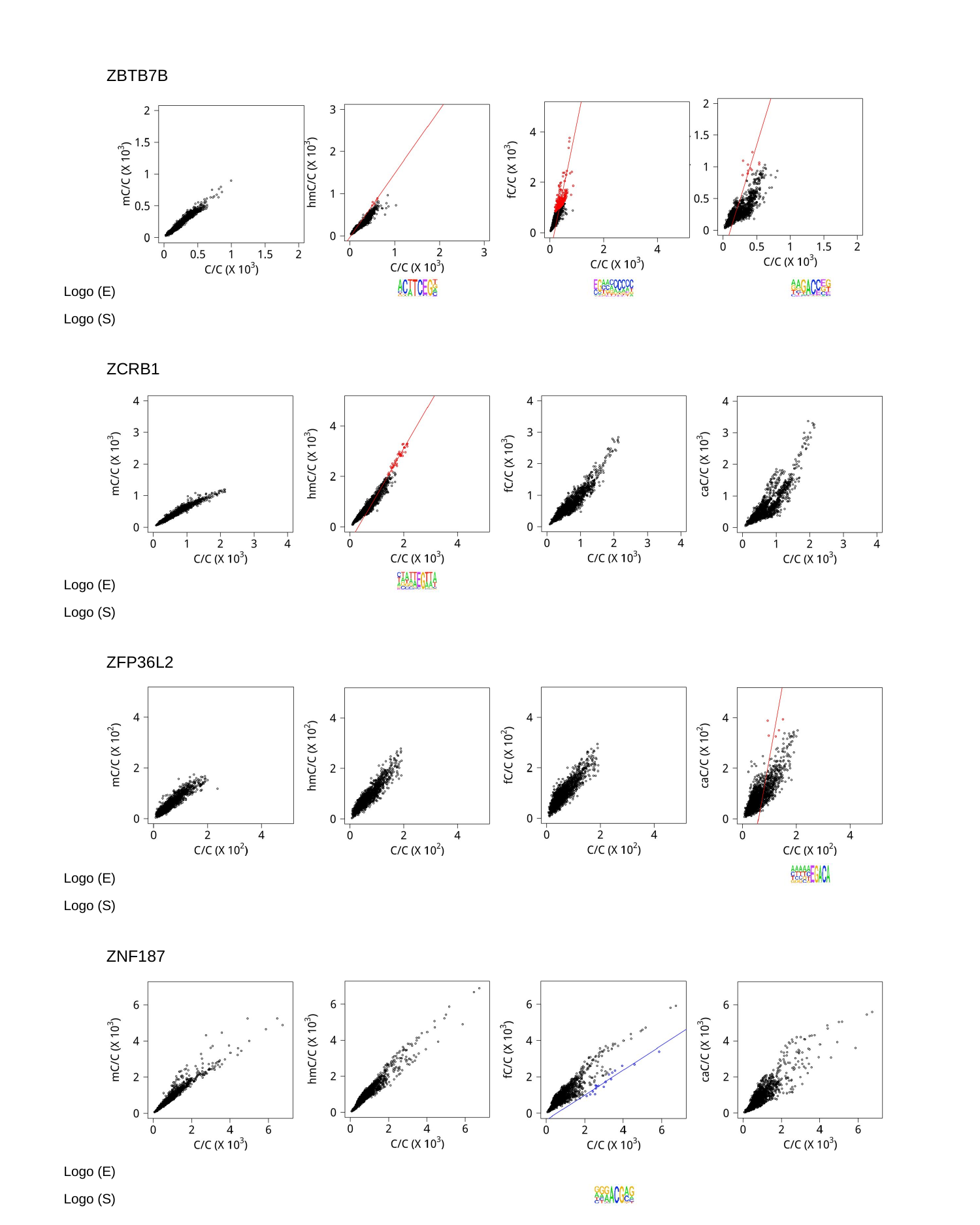

ZBTB7B
Logo (E)
Logo (S)
ZCRB1
Logo (E)
Logo (S)
ZFP36L2
Logo (E)
Logo (S)
ZNF187
Logo (E)
Logo (S)

## Slide 17
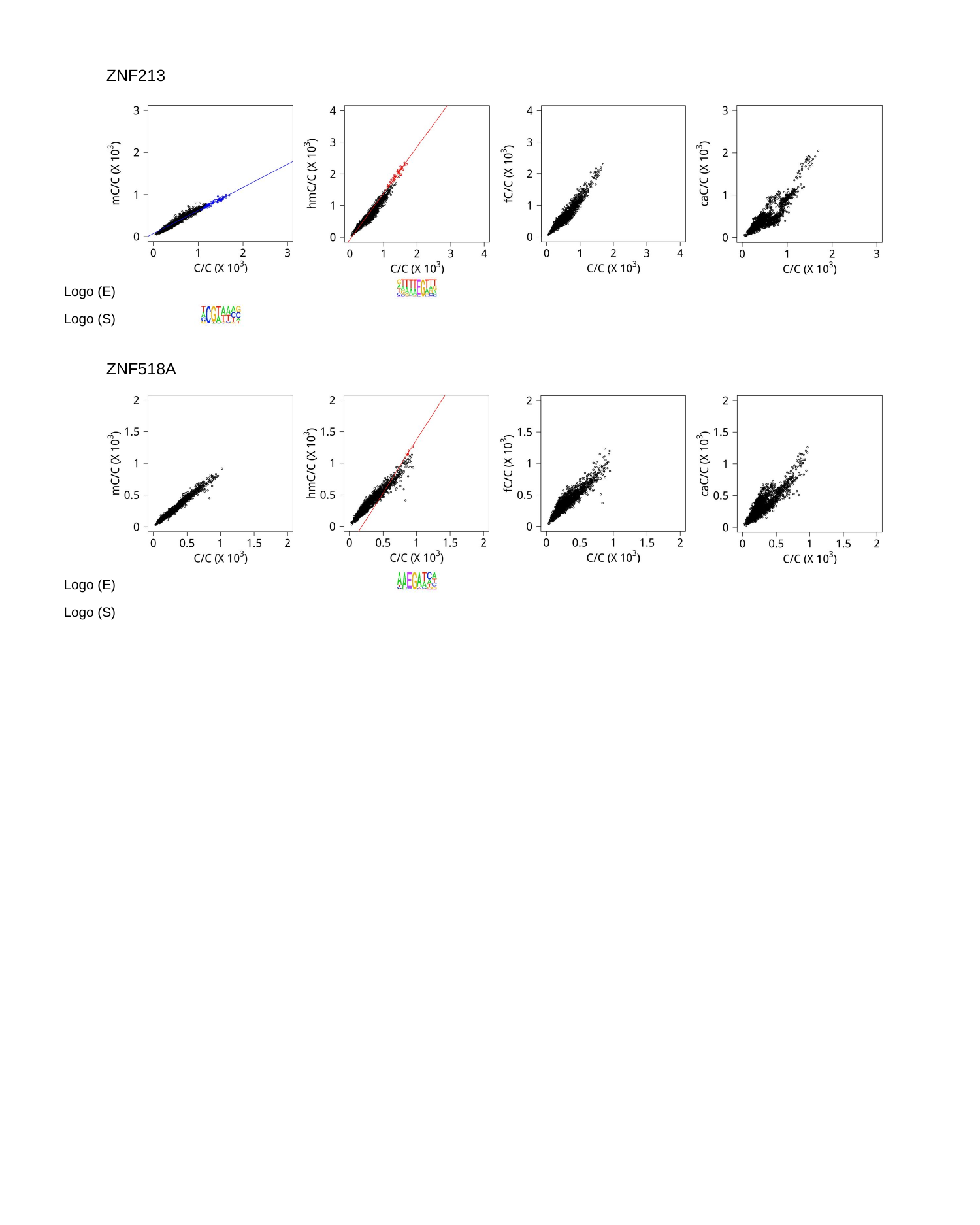

ZNF213
Logo (E)
Logo (S)
ZNF518A
Logo (E)
Logo (S)
