# Supplementary material for: An all-to-all approach to the identification of sequence-specific readers for epigenetic DNA modifications on cytosine
Source: Nat Commun. 2021 Feb 4;12:795. doi: 10.1038/s41467-021-20950-w (PMC7862700; doi:10.1038/s41467-021-20950-w)
Supplement: Supplementary file 12 — Reporting Summary [file 41467_2021_20950_MOESM12_ESM.pdf]

nature research

Corresponding author(s): Heng Zhu; Jiang Qian

Last updated by author(s): Dec 5, 2020

Reporting Summary

Nature Research wishes to improve the reproducibility of the work that we publish. This form provides structure for consistency and transparency in reporting. For further information on Nature Research policies, see our Editorial Policies and the Editorial Policy Checklist.

Statistics

For all statistical analyses, confirm that the following items are present in the figure legend, table legend, main text, or Methods section.

n/a	Confirmed	
☐	☒	The exact sample size (*n*) for each experimental group/condition, given as a discrete number and unit of measurement
☒	☐	A statement on whether measurements were taken from distinct samples or whether the same sample was measured repeatedly
☐	☒	The statistical test(s) used AND whether they are one- or two-sided<br>*Only common tests should be described solely by name; describe more complex techniques in the Methods section.*
☒	☐	A description of all covariates tested
☐	☒	A description of any assumptions or corrections, such as tests of normality and adjustment for multiple comparisons
☐	☒	A full description of the statistical parameters including central tendency (e.g. means) or other basic estimates (e.g. regression coefficient) AND variation (e.g. standard deviation) or associated estimates of uncertainty (e.g. confidence intervals)
☐	☒	For null hypothesis testing, the test statistic (e.g. *F*, *t*, *r*) with confidence intervals, effect sizes, degrees of freedom and *P* value noted<br>*Give P values as exact values whenever suitable.*
☒	☐	For Bayesian analysis, information on the choice of priors and Markov chain Monte Carlo settings
☒	☐	For hierarchical and complex designs, identification of the appropriate level for tests and full reporting of outcomes
☐	☒	Estimates of effect sizes (e.g. Cohen's *d*, Pearson's *r*), indicating how they were calculated

Our web collection on statistics for biologists contains articles on many of the points above.

Software and code

Policy information about availability of computer code

Data collection	Data of TF-DNA binding kinetics and affinity measurement were collected by FortéBio's Data Acquisition 7.1, and analyzed using the FortéBio's Data Analysis 7.1. Images of EMSA assay were collected and analyzed with LI-COR Image Studio Lite Ver 5.2
Data analysis	The software used in this manuscript include MACS2(v2.1.1), IDR(v2.0.2), MEME(v4.11.4) , Tomtom (v5.0.5) , R(v3.5.2) and liftOver. The liftOver has no version number. Here is the download link for liftOver : https://genome-store.ucsc.edu/. The R packages used include Biostrings_2.50.2, seqinr_3.4-5, GenomicRanges_1.34.0, ggseqlogo_0.1. The known motif database is CIS-BP 2.00. All of the computer programs and scripts used are publicly available at https://github.com/HitTracy/DAPPL and the DOI (https://doi.org/10.5281/zenodo.4308235).

For manuscripts utilizing custom algorithms or software that are central to the research but not yet described in published literature, software must be made available to editors and reviewers. We strongly encourage code deposition in a community repository (e.g. GitHub). See the Nature Research guidelines for submitting code & software for further information.

Data

Policy information about availability of data

All manuscripts must include a data availability statement. This statement should provide the following information, where applicable:

- Accession codes, unique identifiers, or web links for publicly available datasets
- A list of figures that have associated raw data
- A description of any restrictions on data availability

All the raw and processed Sequence data that support the findings of this study have been deposited in the NCBI Gene Expression Omnibus (GEO) database under the accession codes [GSE160457; (https://www.ncbi.nlm.nih.gov/geo/query/acc.cgi?acc=GSE160457)]. A source data file is provided with the manuscript.

Field-specific reporting

Please select the one below that is the best fit for your research. If you are not sure, read the appropriate sections before making your selection.

☒ Life sciences ☐ Behavioural & social sciences ☐ Ecological, evolutionary & environmental sciences

For a reference copy of the document with all sections, see nature.com/documents/nr-reporting-summary-flat.pdf

Life sciences study design

All studies must disclose on these points even when the disclosure is negative.

Sample size	The DAPPL experiments using ETS members included all the 28 ETS subfamily TFs in our human ORF clones collection to benchmark the new approach. As the DAPPL experiments to identify the readers of epigenetic modifications, based on our TF clones collection, all of the 1,612 TF associated clones represent 1,235 unique TF or co-factors genes involved into DAPPL. For DNA probe library, the oligo sequence were randomly synthesized. The sequencing depth of DAPPL and 5hmC-ChIP libraries was set to ensure that at least hundreds of thousands unique reads are available for each TF. If a TF prefer binding to DNA oligos carrying any types of epigenetic cytosine modifications, the corresponding non-random pattern of binding oligos could produce a specific binding motifs with an extremely small p value.
Data exclusions	No data were excluded from the analysis.
Replication	All attempts at replication were successful. The DAPPL experiments using ETS members have triplicate technical replicates, for binding affinity and kinetics, at least three replicates were available by OCTET using series of diluted samples. The DAPPL experiments for identifying the readers of epigenetic modifications were performed using five random DNA libraries, one third of TFs have at least two clones located at different 96-well plates in our collection and technique repeatedly involved into all steps in DAPPL assay. Two replicates are available for the in vitro validation EMSA assay and 5hmC-ChIP.
Randomization	All TF or co-factors were randomly divided into eight groups for the DAPPL assay. For the mixture of DNA libraries, all the five libraries, including four modified libraries and one unmodified library as control, were designed as random and combined with same ratio.
Blinding	Blinding was not relevant for this study.

Reporting for specific materials, systems and methods

We require information from authors about some types of materials, experimental systems and methods used in many studies. Here, indicate whether each material, system or method listed is relevant to your study. If you are not sure if a list item applies to your research, read the appropriate section before selecting a response.

Materials & experimental systems

n/a	Involved in the study	
☒	☐	Antibodies
☐	☒	Eukaryotic cell lines
☒	☐	Palaeontology and archaeology
☒	☐	Animals and other organisms
☒	☐	Human research participants
☒	☐	Clinical data
☒	☐	Dual use research of concern

Methods

n/a	Involved in the study	
☐	☒	ChIP-seq
☒	☐	Flow cytometry
☒	☐	MRI-based neuroimaging

Eukaryotic cell lines

Policy information about cell lines

Cell line source(s)	Human ES H1 cells were purchased from WiCell (WAe001-A)
Authentication	The cell line was not authenticated.
Mycoplasma contamination	H1 cell were not tested for mycoplasma contamination
Commonly misidentified lines (See ICLAC register)	No commonly misidentified cell lines were used.

ChIP-seq

Data deposition

☒ Confirm that both raw and final processed data have been deposited in a public database such as GEO.

☒ Confirm that you have deposited or provided access to graph files (e.g. BED files) for the called peaks.

Data access links<br>*May remain private before publication.*	All the raw and processed Sequence data that support the findings of this study have been deposited in the database under the accession codes [GSE160457; (https://www.ncbi.nlm.nih.gov/geo/query/acc.cgi?acc=GSE160457)]. A source data file is provided with the manuscript.
Files in database submission	Raw Data:<br>H1_hESC_hmCG_Rep1_peaks.xls<br>H1_hESC_hmCG_Rep2_peaks.xls<br>Processed Data:<br>H1_genome_hmC-seq_Rep1_fraction_001.fastq.gz<br>H1_genome_hmC-seq_Rep1_fraction_002.fastq.gz<br>H1_genome_hmC-seq_Rep2_fraction_001.fastq.gz<br>H1_genome_hmC-seq_Rep2_fraction_002.fastq.gz
Genome browser session<br>(e.g. UCSC)	UCSC

Methodology

Replicates	Two technical replicates are available for 5hmC-ChIP experiment.
Sequencing depth	Total number of reads: > 30 million<br>Uniquely mapped > 15 million<br>Length of the reads: 150<br>Pair-end
Antibodies	No antibody applied in this study. Here, the ChIP-seq is referred to the hmC-seq, in which the hmC-specific fragments were chemical labelled and enriched using a previously described method (PMID: 29790956).
Peak calling parameters	Poisson-based peak identification algorithm (MACS) using unique non-duplicate reads (effective genome size = 1.87 × 109, tag size = 38, bandwidth = 200, P value cutoff = 1.00 × 10−5; ranges for calculating regional lambda: peak region = 200 and 1,000; mfold values for modeling peaks were adjusted per pairwise comparison).
Data quality	P value cutoff = 1.00 × 10−5
Software	MACS2(v2.1.1), IDR(v2.0.2), MEME(v4.11.4) , Tomtom (v5.0.5), liftOver (https://genome-store.ucsc.edu/), R(v3.5.2)
